# Water Deficit, Nitrogen Availability, and Their Combination Differently Affect Floral Scent Emission in Three Brassicaceae Species

**DOI:** 10.1007/s10886-022-01393-z

**Published:** 2022-12-16

**Authors:** Rebecca J. Höfer, Manfred Ayasse, Jonas Kuppler

**Affiliations:** Institute of Evolutionary Ecology and Conservation Genomics, Ulm University, Ulm, Germany

**Keywords:** Brassicaceae, Climate change, Water deficit, Floral scent, Nitrogen

## Abstract

Floral scent plays a central role in plant-pollinator interactions, as flower visitors can discriminate between scent differences to recognize and forage on rewarding flowers. Changes in scent compositions might therefore lead to recognition mismatches between host plants and flower visitors. An understanding of the phenotypic plasticity of floral scent, especially in crop species, is becoming important because of climate change, e.g., increasing drought periods, and other anthropogenic influences, e.g., nitrogen (N) deposition. We have investigated the effects of the combination of progressive water deficits (dry-down) and N supplementation on floral scent emission in three Brassicaceae species (cultivated vs. wild). Individuals were randomly assigned to one of four treatments: (1) well-watered without N supplementation; (2) well-watered with N supplementation; (3) dry-down without N supplementation; (4) dry-down with N supplementation. We collected scent on day 0, 2, 7, and 14 after the commencement of the watering treatment. All samples were analyzed using gas chromatography coupled with mass spectrometry. We found that the highly cultivated *Brassica napus* had the lowest overall emission rate; its scent composition was affected by the interaction of watering treatment and N supplementation. Scent bouquets of the cultivated *Sinapis alba* also changed under these treatments. Scent bouquets of the common weed *Sinapis arvensis* were affected by watering treatment, but not by time and N supplementation. Furthermore, the influence of treatments on the emission rate of single compounds was highly compound-specific. Nonetheless, our study revealed that especially terpenes were negatively affected by drought-stress.

**Supplementary Information:**

The online version contains supplementary material available at 10.1007/s10886-022-01393-z.

## Introduction

More than 85% of flowering plant species depend on insect-pollination (Ollerton et al. 2011) and emit a broad spectrum of floral volatile organic compounds (VOCs) to attract pollinators (Pellmyr and Thien 1986; Armbruster 1997; Schiestl 2010). Consequently, floral scent plays a central role in plant-pollinator interactions (Raguso 2008; Wright and Schiestl 2009; Dötterl and Vereecken 2010). Floral visitors can differentiate between intra- and interspecific quantitative and qualitative differences in floral scents and thereby recognize rewarding flowers for the initiation and maintenance of foraging on flowers (Andersson 2003; Andersson and Dobson 2003; Wright et al. 2005). Floral scent emission and composition are not static and can (1) differ between floral parts (spatial variation) to inform the flower visitor about the location of the reward (Raguso 2004; Dötterl and Jürgens 2005; Burdon et al. 2015), (2) vary throughout floral development or with a circadian rhythm (temporal variation) to match the pollinators’ activity (Dötterl et al. 2012; Borges et al. 2013; Jürgens et al. 2014), and (3) change with environmental conditions, such as temperature and the availability of soil nutrients or soil water (Majetic et al. 2009; Yuan et al. 2009; Farré-Armengol et al. 2014; Burkle and Runyon 2016). Modifications of the rate and abundance of VOC emissions and changes in the ratios of scent compounds can confuse pollinators (Gérard et al. 2020), leading to functional and recognition mismatches between host plants and flower visitors (Miller-Struttmann et al. 2015; Descamps et al. 2020; Gérard et al. 2020) and to reduced visitation rates (Descamps et al. 2021; Kuppler et al. 2021; Kuppler and Kotowska 2021). However, we are only now beginning to understand the causes of the variation in floral VOCs and the roles that are played by environmental conditions (Raguso et al. 2015). Knowledge of phenotypic plasticity in response to a changing environment has become even more important when exploring changing climate, e.g., decreasing precipitation or increasing drought periods (IPCC 2014), and other anthropogenic changes, e.g., increasing nitrogen (N) deposition in agriculture.

Water deficit can affect floral scent emission (Burkle and Runyon 2016; Glenny et al. 2018; Campbell et al. 2019), with the magnitude of the effect depending on the starting point of the water deficit, its duration, and its intensity. If water deficit starts before flowering and lasts for several weeks, plants seem to be able to adjust their physiology to the new conditions and compensate for reduced water availability by reducing growth to maintain floral traits such as scent emission for attracting pollinators (Höfer et al. 2021; Kuppler and Kotowska 2021). Pulsed water deficit at the beginning of flowering, when water-stressed plants are watered to the point of saturation once they show signs of wilting, results in higher total scent emission and a changed composition of floral volatiles (Burkle and Runyon 2016; Glenny et al. 2018; Rering et al. 2020). Water deficit plants have been demonstrated to emit more (*Z*)-3-hexenol, 6-methyl-5-hepten-2-one, benzaldehyde, α- and β-pinene, (*E*)-β-ocimene, and (*E,E*)-α-farnesene and less 1,3-octadiene and benzyl alcohol, depending on the species and study (Burkle and Runyon 2016; Glenny et al. 2018; Campbell et al. 2019; Rering et al. 2020). Such changes might be the result of specific biochemical pathways being up- or down-regulated. However, most of the above studies have focused on a comparison of floral scent emissions under dry vs. watered conditions, rather than examining emissions in response to the stress caused by progressively increasing water deficit (Campbell et al. 2019).

Nitrogen (N) availability has also been shown to have significant effects on floral scent emission (Majetic et al. 2017). Methyl salicylate, acetic acid, and several products of the lipoxygenase pathway increase under N supplementation (Veromann et al. 2013). Moreover, benzenoid/phenylpropanoid volatiles synthesized from the N-containing amino acid phenylalanine through the shikimic acid pathway are expected to increase with N supplementation (Dudareva and Pichersky 2000; Majetic et al. 2008; Majetic et al. 2017). Furthermore, other floral volatiles such as terpenoids or fatty acid derivatives should not be affected by increasing N availability, as they are not derived from N-containing compounds (Dudareva and Pichersky 2000; Majetic et al. 2008).

Facing extended and more frequent droughts due to climate change, as well as increased N application in agricultural landscapes, it is important to investigate if and how reduced soil water- and soil N availability might interact with one another and impact floral scent. In general, N availability under water deficit increases membrane stability and maintains turgor pressure (Saneoka et al. 2004), reduces oxidative damage, and enhances photosynthesis by increasing stomatal conductance (Waraich et al. 2011a, b). Enhanced photosynthesis might then compensate for the carbon cost of scent production (Farré-Armengol et al. 2014) and maintain scent emission under water deficit. In maize (*Zea mays* L.), increased N availability increased stem diameter and improved breaking resistance (Ye et al. 2016), which could help plants to be more resistant to water deficit as well. Further, an adequate N supply can enhance the tolerance of plants to water deficits and can increase the efficiency of water use (Quemada and Gabriel 2016; Akter and Klečka 2020). Combined treatments of water deficit and nutrient availability have revealed that the scent emission rate of flowering plants is higher when nutrients are abundant. However, such treatments seem to not affect floral scent composition (Luizzi et al. 2021). Nevertheless, the combined effect of water deficit and N availability on floral traits (e.g. floral scent) needs further investigation, especially for flowering crop species, with regard to the possible mitigation effect of N supplementation on drought stress.

For our study, we selected three species from the plant family Brassicaceae to investigate the effects of combined environmental factors on floral scent, an important floral trait for pollinator attraction. These species are annual plants and easy to rear in greenhouses and therefore, are well-suited as model organisms. Further, the Brassicaceae family is of particular agricultural importance. It consists of various genera that have a variety of economic and agronomic uses, with *Brassica* being the most important genus of the family as many of our crop plant species are included in this genus providing edible buds, leaves, and flowers (*Brassica oleracea*, including many cultivars) (Hasanuzzaman 2020). The genus *Sinapis* includes species that are used to produce condiments, and thus have high agronomic value (Rakow 2004). Although many flowering crop species can self-pollinate, they benefit from insect pollination, for instance soybean (*Glycine max* L. Merril) and oilseed rape (*Brassica napus* L., Brassicaceae), as insect pollination increases yield by more than half (Chiari et al. 2005; Araneda Durán et al. 2010). *Brassica napus* emits a complex mixture of floral volatiles (Bartlet et al. 1993; Schiestl 2010) and is a dominant flowering crop cultivated for its oil-rich seeds, which are the third most important source of edible oil (van der Velde et al. 2009; El-Beltagi and Mohamed 2010; Hasanuzzaman 2020). However, a gap exists in our knowledge of the way that stress from water deficit affects floral scent emission in crop species (Reinhardt 2015), with possible consequences for pollinator attraction. Therefore, we need to understand the role of floral volatiles in crop production and any effects of abiotic stressors such as water or nutrient deficiency on floral scent emission. For *B. napus* it has been shown that total emission of volatiles is higher in plants under short-term water deficit than in control plants. The identified compounds are mostly similar between the watering treatments, although the ratio between the compounds differs between drought-stressed and control plants (Reinhardt 2015). In the related species *Sinapis arvensis* L., a common weed species, scent emission, and composition do not seem to be affected by water deficit persisting for several weeks (Höfer et al. 2021). Nonetheless, we are lacking studies that investigate how floral scent emission changes over time under short (several days) progressive water deficit, combined with altered nutrient availability.

In this study, we have investigated the effects of the combination of the progressive increase of water deficit (dry-down) and N availability on floral scent emission. We selected three Brassicaceae species, two cultivated species (*Brassica napus* and *Sinapis alba*), and one common wild species (*Sinapis arvensis*) to compare how cultivation may play a role in the response to changes in environmental factors. We asked the following two questions. (**1**) Is the scent emission rate and composition affected by time (due to possible increased water deficit), watering-treatment, and N supplementation per-se? Increasing water deficit might cause scent emissions to become highly plastic or they might be stably maintained. Moreover, the stress response might differ between cultivated and wild species as they possibly differ in their resource investment in reproductive traits. (**2**) How do important scent compounds respond to treatments over time, and which classes of floral volatiles are mainly affected? Compounds directly related to N metabolism may be stronger affected by N availability than compounds derived from different pathways.

### Methods and Materials

#### Plant Organisms

We selected three species from the cabbage family Brassicaceae: (1) *Sinapis arvensis* L., wild mustard (purchased from Templiner Kräutergarten, Templin, Germany); (2) *Sinapis alba* L., white or yellow mustard (cultivar ‘Litember’, purchased from Kiepenkerl, Aurich, Germany); (3) *Brassica napus* L., oilseed rape (summer rape cultivar ‘Liforum’, purchased from Insektensaatgut, Lünen, Germany). All three species have bright yellow tetramerous flowers, with petals of equal size and shape (Warwick et al. 2000; Gulden et al. 2008; Franzke et al. 2011). As generalist species, they attract a broad range of insect species such as bees and syrphid flies (Kunin 1993; Kobayashi et al. 2012), with honey bees being one of the main pollinators of *B. napus* (Kobayashi et al. 2012). *Sinapis arvensis* is an annual self-incompatible common weed species growing in disturbed habitats. *Sinapis alba* is an annual species that is cultivated worldwide for its seeds to make the condiment mustard (Ekanayake et al. 2016). It is also grown as a cover crop, as green manure, and as a fodder plant (Bodson 2019). *Brassica napus* is the most important oil crop of the family, is a highly cultivated crop species, and is grown for its oil-rich seeds. We investigated these species to determine whether related species that differ in their grade of cultivation (low/zero: *S. arvensis* to high: *B. napus*) showed greater or lower phenotypic plasticity when faced with different abiotic stresses, i.e., reduced water availability and/or a lack of nutrients.

#### Treatments

The seeds were treated with aqueous gibberellic acid solution (1000 ppm; Sigma, St. Louis, MO, United States) and left on wet filter paper in a closed Petri dish in darkness at room temperature until they germinated. Thereafter, the seeds were transferred into 1.3-liter pots containing a soil mixture of low nutrients of 1:1 loamy topsoil:sand. Topsoil was obtained from an area in the Botanical Garden of Ulm University. Once the cotyledons had emerged (~ 3 days), we transplanted the seeds individually into 1.3-liter pots containing the same soil mixture. Two weeks after transplantation, all individuals were fertilized with 0.415 g ( ≙ 0.05 g N) granulated fertilizer (Blaukorn Entec spezial NPK-Dünger 12 + 12 + 12(+ 2 + 8), Compo GmbH, Münster, Germany), to ensure that the growth of the plants was not limited by the lack of nutrients other than N.

We reared 60 individuals per species in a greenhouse in the Botanical Garden of Ulm. From each species, all individuals were randomly assigned to one of four treatments (15 individuals per species per treatment): (1) well-watered without N supplementation (N- Watered); (2) well-watered with N supplementation (N + Watered); (3) dry-down without N supplementation (N- Drought); (4) dry-down with N supplementation (N + Drought). Aqueous N fertilizer (30% N solution, UAN: 15% urea, 8% ammonium (NH_4_^+^), 7% nitrate (NO_3_^−^); Raiffeisen Rhein-Ahr-Eifel Handelsgesellschaft mbH, Germany) dissolved in water (128 µl N fertilizer in 50 ml water) and was carefully applied to the soil of plants in the N supplementation treatment at three weeks after sowing by which time the plants had reached 10 cm in height. This treatment was repeated after four days. Hence, each plant received a total of 256 µl of aqueous N fertilizer ( ≙ 0.1 g N) per plant, together with granulated fertilizer, meaning that the application of N was equivalent to ~ 50 kg/ha (Hoover et al. 2012). Watering started once at least five flowers per plant had emerged. On day 0 of the experiment, plants of all treatments were watered to the extent that the soil was saturated. The plants that were to experience water deficit were allowed to dry progressively and were watered with 30 ml water only when they showed signs of wilting. Plants in the watered treatment were watered daily to saturation point. Here, we defined drought as the absence of precipitation (Slette et al. 2019) and used it as a synonym for reduction in water availability.

In total, 91 individuals survived until flowering and were used in the experiment: *B. napus*: N- Watered *n* = 7, N- Drought *n* = 7, N + Watered *n* = 11, N + Drought *n* = 11; *S. alba*: N- Watered *n* = 3, N- Drought *n* = 3, N + Watered *n* = 10, N + Drought *n* = 10; *S. arvensis*: N- Watered *n* = 6, N- Drought *n* = 6, N + Watered *n* = 9, N + Drought *n* = 8 (see Notes S1 in supplements for further information for survival and mortality rate).

#### Stress Measurement

To ascertain the soil water status, we measured soil humidity by using a self-made soil humidity sensor with the Arduino system (Iduino ME110, Arduino software version 1.8.8, board: Genuino Uno), calibrated with a volumetric water content sensor (HydroSense II with 12 cm rod, accuracy ± 3%, Campbell Scientific, Ltd., Bremen, Germany). The soil of the dry-down treatment became very dry, to the point it was impossible to insert the sensor into the hard soil. Therefore, the mean soil humidity of the dry-down plants was much lower as the lower measuring points are missing.

To confirm that plants experienced drought stress, we measured the pre-dawn and mid-day stem water potential (SWP) on day 7 in one leaf per plant by using a Scholander pressure chamber (Model 1505D, PMS Instrument Company, Albany, Oregon, USA) (McCutchan and Shackel 1992; Naor 2000): the more negative the measured values, the higher the stress. *B. napus*: N- Watered *n* = 7, N + Watered *n* = 11, N- Drought *n* = 7, N + Drought *n* = 11; *S. alba*: N- Watered *n* = 3, N + Watered *n* = 10, N- Drought *n* = 3, N + Drought *n* = 11; *S. arvensis*: N- Watered *n* = 1, N + Watered *n* = 3, N- Drought *n* = 1, N + Drought *n* = 1.

#### Scent Collection and Analysis

To test the effects of watering treatment and N supplementation on the amount and quality of scent emission, we collected scent of each plant by using dynamic headspace sampling at four time points after watering treatment had started: on day 0 (all plants were well-watered) and after 2 days, 7 days, and 14 days. Plants without N supplementation ceased to flower earlier compared with plants with N supplementation. This is why the sample sizes dropped after day 7 for *S. alba* and *B. napus* plants, as they failed to produce new flowers. For *B. napus*, no plant individuals in the water deficit treatment, irrespective of N supplementation, produced flowers until day 14, which is why we could not collect scent data for these treatments. Plants were watered after scent collection (dry-down plants only if needed). All flowers per plant were enclosed within an oven bag (Toppits® Bratschlauch, polyester, 15 × 15 × 30 cm; Toppits, Minden, Germany). The emitted volatiles were pumped out for 120 min and were trapped on 1.5 mg Tenax (mesh 60–80; Supelco, Bellefonte, PA, USA) and 1.5 mg Carbotrap B (mesh 20–40; Supelco) in a quartz vial (length 20 mm, inner diameter 2 mm) by using a membrane pump (G12/01 EB; ASF Rietschle-Thomas, Puchheim, Germany) at a flow rate of 200 ml min^− 1^. Samples were collected between 08:00 and 14:00 h. Scent samples were analyzed using an automatic thermal desorption system (TDU, Gerstel, Mühlheim a. d. Ruhr, Germany) and a cold-injection apparatus (CIS 4 C, Gerstel) coupled with a GC-MS (7890B GC – 5977 A MSD, Agilent Technologies, Germany). The GC-MS was equipped with a DB-5MS silica column (30 m long, inner diameter 0.25 mm, film thickness 0.25 μm), and the column flow (carrier gas: helium) was set to 1.5 ml min^− 1^. The GC oven temperature was set initially at 40 °C, was then increased by 6 °C per min to 250 °C, and was subsequently held constant for 1 min. The MS interface was set at 250 °C. Mass spectra were taken at 70 eV (in EI mode) from m/z 30 to 350. The GC/MS data were analyzed using the GCMSsolution package (Version 2.72, Shimadzu Corporation, Kyoto, Japan). For compound identification we used three criteria: a = using authentic standards, b = RI and mass spectra consistent with values in Adams (2007), NIST Webbook or Pherobase, and c = high MS library match (> 91%; if no standard was available and RI does not match published values). The compounds that we found in the flowers were compared with those found in blank controls (empty oven bags and samples of green leaves) and were checked using ‘The Pherobase’ to determine those compounds that were emitted in particular by flowers. Scent compounds selected for further analysis were found in at least two samples. The classification of scent compounds was assessed using the freely available PubChem database and Junker et al. 2018. The amount of each compound emitted per flower per hr for each sample (ng/h/flower) was estimated by comparing peak areas with the area of the liquid injected standard (Octadecane, C18) peak and by dividing that amount by the number of hrs (2 h) during which volatiles were collected and by the number of flowers enclosed in the oven bag. We found that all three species significantly differ in their scent bouquets at day 0 without N supplementation (see Notes S2 in supplements for further information).

#### Statistical Analyses

All data were analyzed (except for PERMANOVA) and plotted using R (version 4.1.0, R Core Team 2021). To find differences in soil humidity between treatments and time points, we performed linear models by using the *lm*()-function from the stats-package 4.1.0 (R Core Team 2021) for each species with humidity as the dependent factor and with watering treatment and N supplementation, timepoints and their interactions as fixed factors. PlantID as random factor was removed because of singular fit. The same was carried out for stem water potential (SWP) with SWP as the dependent factor and the watering treatment and N supplementation, day time, and their interactions as fixed factors. PlantID as random factor was also removed because of singular fit.

We had to exclude day 14 for *B. napus* from statistical analyses, as only 4 plants produced flowers until this day. The effects of time and treatments on the total scent emission for each species were tested by fitting linear mixed-effects models (LMM) by using the *lmer*()-function from the *lme4*-package 1.1–27 (Bates et al. 2015) for *S. alba* and *S. arvensis*. For *B*. *napus*, we fitted a linear model by using the *lm*()-function because of singular fit. Total scent emission per flower in ng/h/flower was set as the dependent variable and was log1p-transformed to achieve a normal distribution; watering treatment and N supplementation, and timepoints, as well as their interactions were set as fixed factors. To reveal differences between species, we fitted LMM with scent emission (log1p-transformed) as the dependent variable, species as the fixed factor, and plantID as the random factor. All models were fitted with restricted maximum likelihood (REML) and a maximum of 100,000 iterations. Significance was ascertained using Type II Wald chi-square tests implement in the *Anova*() function of the car-package 3.0–10 (Fox and Weisberg 2018). For the pair-wise comparison of differences in means between the levels of fixed factors, a Post hoc test with the “Tukey” adjustment method was performed using the *lsmeans*()-function from the *emmeans*-package 1.7.1-1 (Lenth 2021).

To test the response of the scent composition of each species to watering treatment and to N supplementation over time, we performed permutational analyses (PERMANOVA) using relative amount data for each compound (amount of single compound in ng/h/flower, divided by total scent amount of the sample), with the Bray-Curtis similarity distance matrix separately for each species among treatments and time points. For *S. alba*, unique permutations were all near 9999. However, the pairwise comparison of watering treatments within each time point revealed a weak result, as unique perm value was only at 252. Nevertheless, we still included day 14 in the analysis for *S. alba* as the results were no different from the model without day 14.

We further performed permutational tests for homogeneity of multivariate dispersion (PERMDISP) with 9999 permutations and Bray-Curtis similarity distance matrix to test for differences in scent variability (dispersion) within the treatments. For all permutational analyses, we used (a) sums of squares type III (partial), (b) a permutation of residuals under a reduced model, and (c) 9999 permutations. Model robustness was assessed using the ‘unique permutation’ values, and assumptions were met.

To extract particular scent compounds and classes of compounds that explained the main differences of the scent bouquets between treatments of the plant species, we performed SIMPER-analysis separately for each species (cumulated contribution to dissimilarity for compounds: 33%, and the first three compound classes with the highest contribution per treatment; see Tables S5 and S6). To check the way that their emission was affected by time, N supplementation, and water deficit, and by their interacting effects, we fitted linear models for each species and compound with scent emission as the dependent variable and the former as fixed factors. PlantID as random factor was removed because of singular fit. Scent emission was square-root-transformed to achieve a normal distribution. The *Anova*()-function was used to test for significance. Finally, we investigated the way that total emissions by compound classes were affected by time, N supplementation, water deficit, and by their interacting effects within each plant species. We fitted linear mixed-effects models (LMM) by using the *lmer*()-function for each species and class of compounds, with the relative emission of compound classes as the dependent variable; scent emission was log1p-transformed to achieve a normal distribution with time, N supplementation, and watering being set as fixed factors and plantID as a random factor. All models were fitted with restricted maximum likelihood (REML) and a maximum of 100,000 iterations. The *Anova*()-function was used to test for significance. The assumptions of all models were assessed using the *DHARMa*-package 0.4.1 and were met (Hartig 2020).

PERMANOVA, PERMDISP, and SIMPER were run by using the software PRIMER 6 (version 6.1.15; PRIMER-E Ltd 2012) in combination with add-on PERMANOVA+ (version 1.0.5; PRIMER-E Ltd 2012). We used non-metric multidimensional scaling (NMDS) for ordination to depict differences in the scent bouquet between the species graphically. The *metaMDS*()-function from the *vegan*-package was employed to run the NMDS, with the Bray-Curtis distance matrix and 9999 permutations. See Supplementary Information for Data, Readme and R-Code.

## Results

### Soil Humidity and Stem Water Potential

**Fig. 1:**
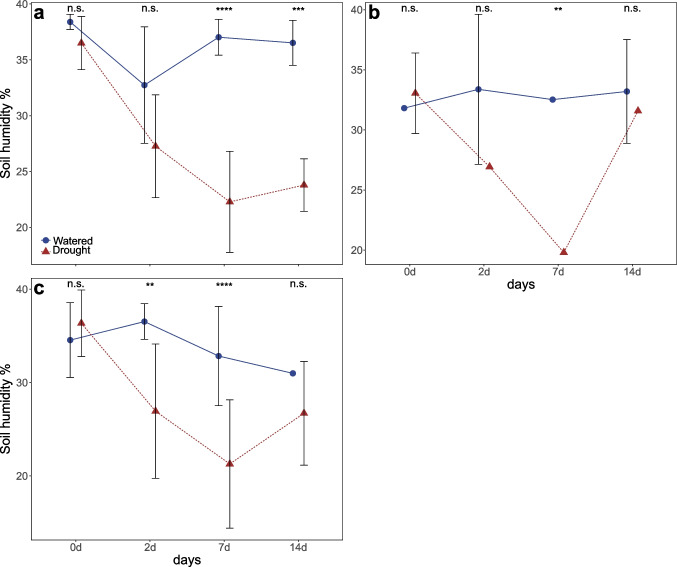
Soil humidity in % for the plant species at four different time points. Blue points show mean humidity for watered treatment. Red triangles show mean humidity for dry-down treatment. (**a**) *B. napus*: day 0: *n* = 9 (Watered *n* = 4, Drought *n* = 5); day 2: *n* = 7 (Watered *n* = 3, Drought *n* = 4), day 7: *n* = 16 (Watered *n* = 8, Drought *n* = 8), day 14: *n* = 10 (Watered *n* = 8, Drought *n* = 2). (**b**) *S. alba*: day 0: *n* = 18 (Watered *n* = 9, Drought *n* = 9), day 2: *n* = 12 (Watered *n* = 6, Drought *n* = 6), day 7: *n* = 15 (Watered *n* = 10, N- Drought *n* = 5), day 14: *n* = 6 (Watered *n* = 4, Drought *n* = 2). (**c**) *S. arvensis*: day 0: *n* = 20 (Watered *n* = 11, Drought *n* = 9), day 2: *n* = 22 (Watered *n* = 12, N- Drought *n* = 10), day 7: *n* = 28 (Watered *n* = 13, Drought *n* = 15), day 14: *n* = 15 (Watered *n* = 7, Drought *n* = 8). Error bars show standard deviation. * and n.s. indicate significant differences between watering treatments within each time point. n.s. = not significant, **P < 0.01, ***P < 0.001, ****P < 0.0001

For *B. napus*, the soil humidity of the watered plants was consistent over time; the soil humidity of the dry-down plants decreased progressively over time and was significantly lower compared with watered plants at days 7 and 14 (Post-hoc Tukey: day 7: *P* < 0.0001, day 14: *P* = 0.0003; Fig. 1a). For *S. alba* plants, the soil humidity of the watered plants was consistent over time; the soil humidity of the dry-down plants decreased progressively over time but increased at day 14. Soil humidity of dry-down plants was significantly lower compared with watered plants only at day 7 (Post-hoc Tukey: day 7: *P* = 0.006; Fig. 1b). For *S. arvensis*, the soil humidity of the watered plants was consistent over time; the soil humidity of the dry-down plants decreased progressively over time, and was significantly lower compared with watered plants at days 2 and 7 (Post-hoc Tukey: day 2: *P* = 0.001, day 7: *P* < 0.0001; Fig. 1c).

The SWP as an indicator of plants’ drought stress showed that in the water-deficient *B. napus* plants SWP was more than twice as negative compared with the watered plants (LM: drought: *F*_1,19_ = 32.43, *P* < 0.0001, Fig. S1A); N supplementation had no effect on SWP, and we found no differences between the daytimes. For *S. alba*, the SWPs of watered and water-deficient plants did not differ at pre-dawn. However, when measured around midday, the SWP of water-deficient plants was more than twice as negative as that for the watered plants; water-deficient plants had a more negative SWP measured at midday, compared with predawn (Post-hoc Tukey: *P* = 0.018). Plants with N supplementation tended to be less stressed than plants without N supplementation (LM: drought: *F*_1,22_ = 22.70, *P* < 0.0001, N supplementation: *F*_1,22_ = 3.21, *P* = 0.087, daytime: *F*_1,22_ = 9.20, *P* = 0.006, Fig. S1B). The SWP of *S. arvensis* plants did not differ between the watered and water-deficient plants; moreover, N supplementation and daytime also did not influence the SWP (Fig. S1C).

### Flowers

The total flower production (as the sum of flowers at each time point) was three times lower in *B. napus* compared with both *Sinapis* species, whereas the latter did not differ from each other. *Sinapis alba* plants in the watered treatment, as well as plants with N supplementation had significantly more flowers than water-deficient plants or plants without N supplementation. However, we found no interacting effect of both treatments. For the other species, treatments did not affect the flower production. The fresh weight per flower did not differ between species; however, fresh weight for *S. arvensis* flowers was two times higher in the watered treatment; N supplementation had no effect. For the other species, treatments did not affect flowers’ fresh weight. See supplementary information for details on flower production during flowering period and flower weight (Notes S3).

### Total Scent Emission

**Fig. 2:**
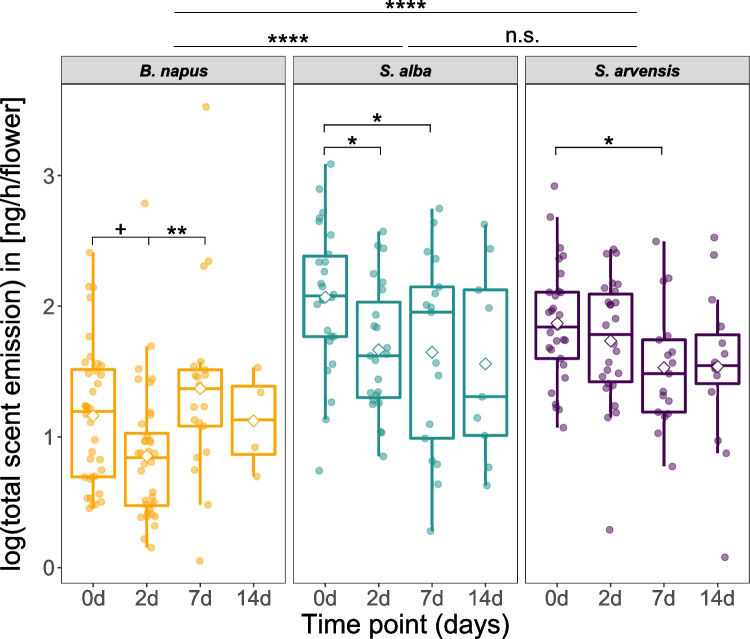
Total amount of scent emission in ng per hr per flower for the plant species at four different time points. As treatment has no significant effect, it was omitted from the graph. *B. napus*: day 0: *n* = 36, day 2: *n* = 36, day 7: *n* = 21, day 14: *n* = 4. Reduced sample numbers at day 7 and 14 are due to lack of flowers in the N- Drought, N + Drought and N- Control treatment. *S. alba*: day 0: *n* = 26, day 2: *n* = 23, day 7: *n* = 17, day 14: *n* = 9. Reduced sample numbers at day 7 and 14 are due to lack of flowers in both N supplementation treatments. *S. arvensis*: day 0: *n* = 29, day 2: *n* = 26, day 7: *n* = 17, day 14: *n* = 15. Each colored point represents one scent sample from one plant. Boxplots show the median range and the 25th and 75th percentile interquartile ranges. Whiskers show the 1.5x interquartile range. White diamonds show mean values. + *P* < 0.1, *P < 0.05, **P < 0.01, ****P < 0.0001, n. s. = not significant

In the floral scent samples of the three species, we identified 112 scent compounds, belonging to seven compound classes. Apart from the identified compounds, we found 27 unidentified compounds (see supplementary information, Table S1). The total scent emission rate in ng per hr and per flower differed among the species (LMM: *χ*^*2*^ = 63.86, df = 2, *P* < 0.0001), with the lowest scent emission in *B. napus* flowers (Post-hoc Tukey: *B. napus* – *S. alba P* < 0.0001; *B. napus* – *S. arvensis P* < 0.0001; Fig. 2). The scent emission of the two *Sinapis* species did not differ from each other. The scent emission of *B. napus* flowers was affected by time (LM: *F*_2,83_ = 5.77, *P* = 0.004), with emission being the highest on day 7 and the lowest on day 2 (Post-hoc Tukey *P* = 0.013). Water-deficient individuals emit more scent than watered plants, however, the differences were not significant (LM: *F*_1,83_ = 3.22, *P* = 0.076). N supplementation did not affect scent emission. The scent emission of *S. alba* decreased over time (LMM: *χ*^*2*^ = 14.09, df = 3, *P* = 0.003; Post-hoc Tukey: 0d – 2d, *P* = 0.029; 0d – 7d, *P* = 0.034). Watering or N supplementation did not affect scent emission. The scent emission of *S. arvensis* flowers was also affected by time (LMM: *χ*^*2*^ = 10.70, df = 3, *P* = 0.013), with the highest emission at day 0. The scent emission decreased after 7 days (Post-hoc Tukey: 0d – 7d, *P* = 0.049; 0d – 14d, *P* = 0.077). Treatments did not affect total scent emission.

### Scent Bouquets, Time, and Treatments

#### *Brassica napus*

**Fig. 3:**
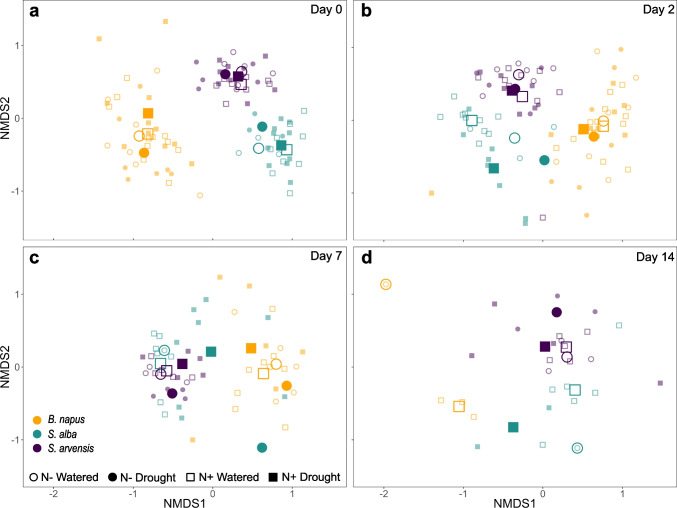
NMDS (non-metric multidimensional scaling) plot projected in two-dimensional space based on Bray-Curtis distances of scent composition for all species (yellow symbols: *Brassica. napus*; green symbols: *Sinapis alba*; dark purple symbols: *Sinapis arvensis*) and treatments (open circles: watered plants without N; filled circles: water-deficient plants without N; open rectangles: watered plants with N; filled rectangles: water-deficient plants with N) at the four time points: (**a**) day 0, (**b**) day2, (**c**) day 7, and (**d**) day 14. Each small colored symbol shows one scent sample; the larger symbols show centroids grouped by species and treatments. Reduced sample numbers for *B. napus* at day 7 and 14 are due to lack of flowers in the N- Drought, N + Drought and N- Control treatment and reduced sample numbers for *S. alba* at day 7 and 14 are due to lack of flowers in both N supplementation treatments

Scent bouquets of individuals were significantly affected by time, as bouquets from day 0 significantly differed from bouquets at the other time points (PERMANOVA: Pseudo-*F*_2,81_ = 1.87, *P* = 0.013), and the interaction of watering treatment and N supplementation (PERMANOVA: Pseudo-*F*_1,81_ = 2.43, *P* = 0.008; Fig. 3a-d, yellow symbols; see statistical details in Table S2a) with the bouquets at day 0 differed significantly from the bouquets collected on the other days (see statistical details for pair-wise analysis in Table S2b). Further, the scent bouquets between watered and water-deficient plants significantly differed with N supplementation, but not without N supplementation (PERMANOVA_N+_: *t* = 1.68, *P* = 0.004; see statistical details in Table S2c). Dispersion/variability of scent bouquets within time points, and within treatments were similar, respectively (PERMDISP_time_: *F*_2,90_ = 1.67, *P* = 0.278; PERMDISP_treat_: *F*_3,89_ = 2.47, *P* = 0.133; see details and distance to centroids in Table S2d and e, respectively).

#### *Sinapis alba*

Scent bouquets of individuals changed over time, as bouquets from day 0 significantly differed from bouquets at the other time points (PERMANOVA: Pseudo*-F*_3,60_ = 2.09, *P* = 0.003; Fig. 3a-d, green symbols, Table S3a and b for pair-wise analysis). Bouquets within each watering-treatment clustered more closely together (PERMANOVA: Pseudo*-F*_1,60_ = 3.67, *P* = 0.001; Table S3a). Bouquets of both N supplementation treatments also significantly differed from each other (PERMANOVA: Pseudo*-F*_1,60_ = 3.13, *P* = 0.004). Scent bouquets were also affected by the interaction of time with watering treatment (PERMANOVA: Pseudo*-F*_3,60_ = 1.70, *P* = 0.027), as the bouquets of watered and water-deficient plants significantly differed at day 7 (Table S3c for pair-wise analysis); as well as by the interaction of watering treatment and N supplementation (PERMANOVA: Pseudo*-F*_1,60_ = 2.27, *P* = 0.026), where bouquets differed between watering treatments with N supplementation (*t* = 2.65, *P* = 0.0001); without N supplementation, bouquets of watering treatments did not differ (*t* = 1.45, *P* = 0.083; Table S3d). Analysis of dispersion revealed that bouquets within day 2 and day 7 were more variable compared with bouquets at day 0 (PERMDISP: *F*_1,71_ = 6.19, *P* = 0.005; Table S3e). Dispersion/variability of scent bouquets of watered and water-deficient plants, and of both N supplementation treatments were similar (PERMDISP: *F*_1,73_ = 1.96, *P* = 0.228, Table S3f; PERMDISP: *F*_1,73_ = 0.01, *P* = 0.933, Table S3g). Dispersion/variability of watering treatments within each time point were similar (PERMDISP: *F*_7,67_ = 2.26, *P* = 0.222; Table S3h); however, pair-wise analysis revealed that variability between time points within the watering treatments was different, as bouquets of water-deficient plants at day 7 were more variable than bouquets of water-deficient plants at day 0 (*t* = 3.52, *P* = 0.012). Dispersion/variability of the interaction of watering treatment and N supplementation did not differ (PERMDISP: *F*_3,71_ = 0.55, *P* = 0.791; Table S3i).

#### *Sinapis arvensis*

Scent bouquets within each watering treatment clustered more closely together (PERMANOVA: *F*_1,71_ = 2.97, *P* = 0.004; Fig. 3a-d, dark purple symbols; see statistical details in Table S4a). Dispersion/variability was similar for both watering treatments (PERMDISP: *F*_1,85_ = 1.25, *P* = 0.309; Table S4b). Neither time nor N supplementation, nor the interaction of factors had an effect on scent bouquets.

#### Response of Important Scent Compounds to Treatments and Changes Over Time

The emission of fatty-acid-derived compounds increased following water deficits and with N supplementation in the *Sinapis* species; in *B. napus*, emission of Linolenic acid decreased over time and under water deficit and with N supplementation (Table 1). For the emission of aromatic compounds in *S. alba* plants, there were differing results. Emission decreased over time but increased with N supplementation. The emission of aromatics in *B. napus* plants was not significantly affected by time and N supplementation. In *S. arvensis*, the emission of aromatics was mainly unaffected by time and treatments; however, emission of Methyl salicylate was reduced in water-deficient plants and in plants with N supplementation. Emission of monoterpene-compounds in *B. napus* was mainly unaffected by treatments; however, Linalool and Myrcene emission decreased over time. In *S. alba*, monoterpene-emission increased mainly over time, and water deficit either decreased ((*E*)-β-Ocimene) or increased (6-Methyl-5-heptene-2-one) emission. For *S. arvensis*, most monoterpene-compounds were unaffected by time and treatment; however, emission of (*E*)-β-Ocimene and (*Z*)-β-Ocimene decreased, whereas emission of Limonene increased under water deficit. Emission of the sesquiterpenes in *B. napus* decreased over time ((*E,E*)-α-Farnesene) or increased with N supplementation ((*E,E*)-α-Farnesene). In *S. alba*, emission increased with time, but decreased with N supplementation for (*E*)-β-Farnesene. Emission of (*E,E*)-α-Farnesene decreased under water deficit, which was also true for *S. arvensis* plants. The emission of the homoterpene (*E*3,*E*7)-4,8,12-Trimethyltrideca-1,3,7,11-tetraene decreased over time and increased with N supplementation ((3*E*)-4,8-Dimethyl-1,3,7-nonatriene) in *B. napus* there were no significant effects of time and treatments for the *Sinapis* species (details for mean emission under time and treatments for each species see in Tables S7 to S9).

**Table 1:**
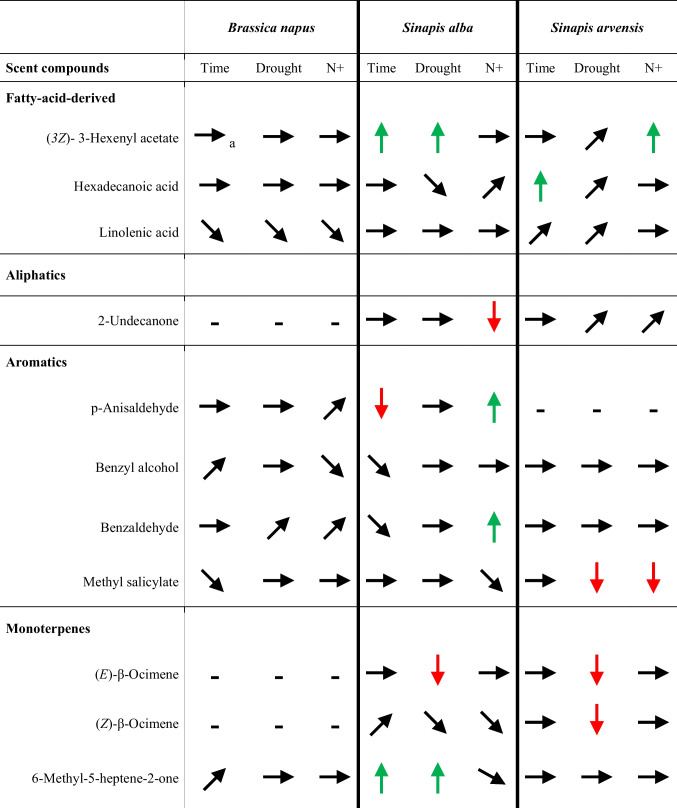

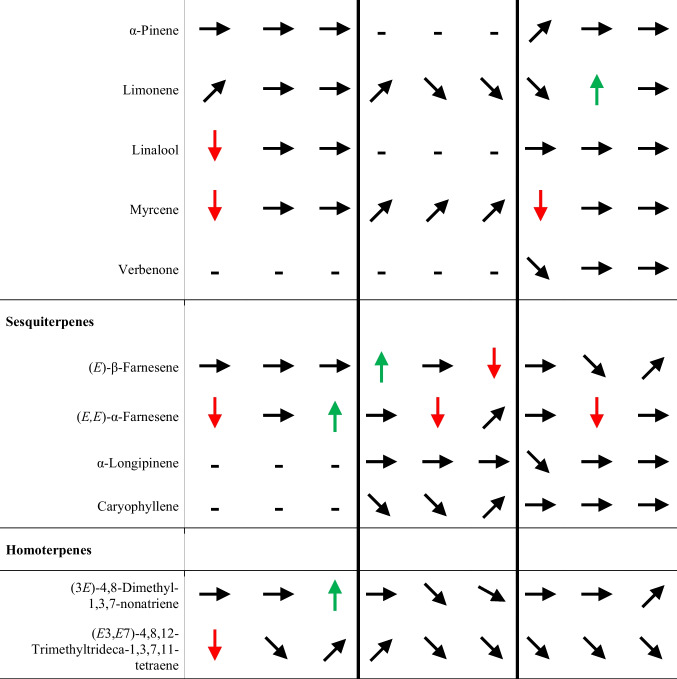
Effect of time, water deficit (“Drought”), and nitrogen (N+) on the emission of selected scent compounds for *Brassica napus*, *Sinapis alba*, and *Sinapis arvensis*

Significance was assessed using Analysis of Deviance Table (Type II tests) (Statistical details are in supplement Tables S7-S9)

^a^ → No effect,  , Increased,  , Decreased,  Tendency (not significant),    - not emitted

#### Compound-class emission

**Fig. 4:**
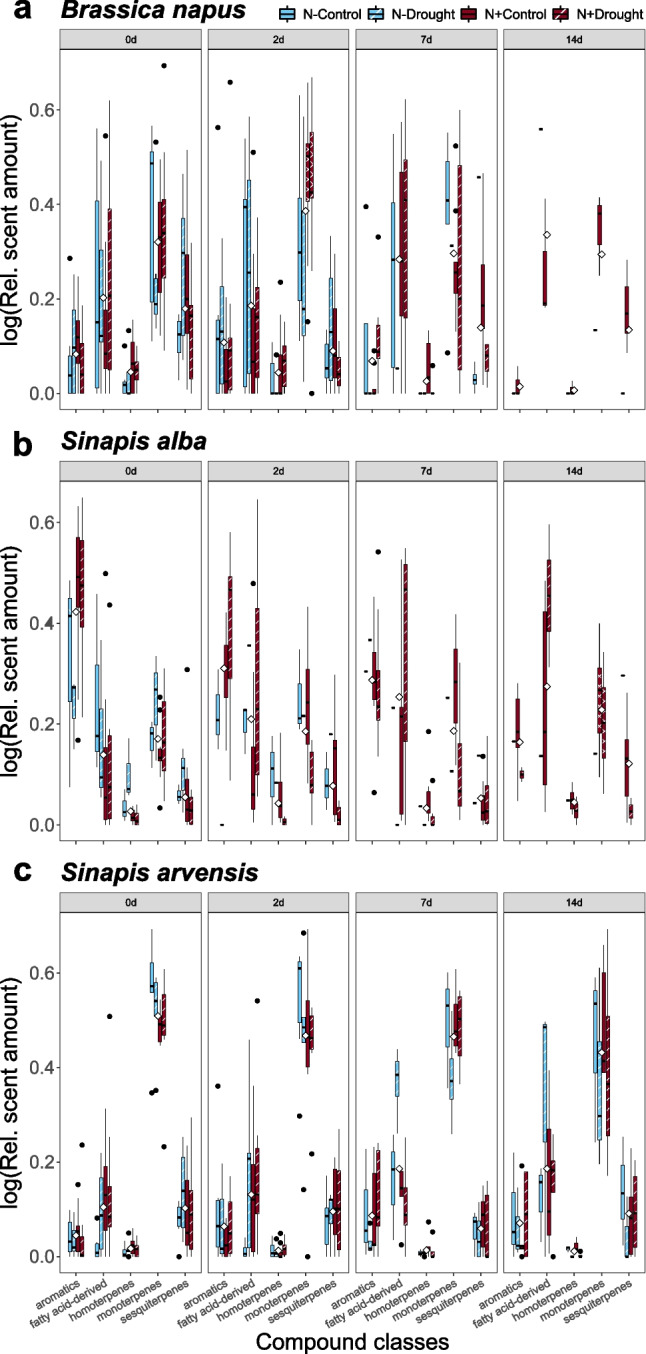
Relative scent emission of those compound classes that are most responsible for differences between species at four time points (0 days, 2 days, 7 days, 14 days) and treatments (light blue: N- Watered; light-blue with white stripes: N- Drought; red: N + Watered; red with white stripes: N + Drought). Scent emission for (**a**) *Brassica napus*; (**b**) *Sinapis alba*; (**c**) *Sinapis arvensis*. Reduced sample numbers for *B. napus* at day 7 and 14 are due to lack of flowers in the N- Drought, N + Drought and N- Control treatment and reduced sample numbers for *S. alba* at day 7 and 14 are due to lack of flowers in both N supplementation treatments. Boxplots show median range and 25th and 75th percentile interquartile ranges. Whiskers show 1.5x interquartile range. White diamonds show mean values grouped by pathway. Black points show outliers

For the emission of scent grouped according to the classes of scent compounds, we found aromatics were most highly emitted in *S. alba* (Post-hoc Tukey: *P* < 0.0001; Fig. 4, statistical details for pair-wise comparisons in Table S10 and mean values in Table S11) and least highly in *S. arvensis.* Monoterpene emission was highest in *S. arvensis* (Post-hoc Tukey: *P* < 0.0001) and lowest in *S. alba* plants (Post-hoc Tukey: *P* < 0.0001), whereas sesquiterpene emission was lowest in *S. alba* and highest in *B. napus* (Post-hoc Tukey: *P* = 0.015). The emission of fatty acid derivatives did not differ between the species. Emission of homoterpenes was lowest in *S. arvensis*, and significantly differed from *S. alba* (Post-hoc Tukey: *P* = 0.031).

#### Aromatics

In *B. napus* and *S. arvensis* plants, the emission of aromatics was unaffected by time and treatments and their interactions. In *S. alba* plants, emission decreased over time (LMM: *P* < 0.0001; Fig. 4b, Table S12) and was highest in plants with N supplementation (LMM: *P* < 0.001).

#### Fatty acid derivatives

The emission of fatty-acid-derived compounds was stable over time within each species and was not affected by watering treatment, N supplementation, or interaction effects in *B. napus* and *S. alba* (Fig. 4a + b, Table S12). In *S. arvensis* plants, emission was twice as high in water-deficient plants (LMM: *P* = 0.033; Fig. 4c, Table S12), and we found an interacting effect of time and N supplementation, as the emission increased five-fold from day 0 to day 7 without N supplementation (Post-hoc Tukey: 0d – 7d, *P* = 0.027), whereas the emission was constant over time with N supplementation. Further, we found an interacting effect of watering treatment and N supplementation, as emission was four times higher in water-deficient plants without N supplementation compared with well-watered plants (Post-hoc Tukey: *P* = 0.034), whereas emission did not differ between watered and water-deficient plants with N supplementation.

#### Monoterpenes

Emission in *B. napus* was stable over time within each species and was not affected by watering treatment, N supplementation, and interacting effects in *B. napus* (Fig. 4a, Table S12). In *S. alba*, emission nearly doubled in watered plants compared with water-deficient plants (LMM_dr_: *P* = 0.047; Fig. 4b). Further, an interacting effect of time and watering was found. Emissions between watered and water-deficient plants were the same at days 0, 2, and 14; at day 7 the emission in water-deficient plants was three times lower than that in watered plants (Post-hoc Tukey: *P* = 0.191). Emission in *S. arvensis* was unaffected by time and treatments and their interactions (Fig. 4c, Table S12).

#### Sesquiterpenes

Emission in *B. napus* was affected by time (LMM: *P* = 0.002; Fig. 4a, Table S12), with the highest emission on day 0. Further, we found an interacting effect of watering treatment and N supplementation: emission was twice as high in watered plants with N supplementation than in watered plants without N supplementation (LMM: *P* = 0.0001; Fig. 4a, Table S12). In *S. alba* plants, emission was only affected by watering treatment, with emission twice as high in watered than in water-deficient plants (LMM: *P* = 0.027; Fig. 4b, Table S12). In *S. arvensis*, the emission decreased over time and was lowest at day 7 (Post-hoc Tukey: 0d – 7d, *P* = 0.031; Fig. 4c, Table S12). Further, we found an interacting effect of time and watering. The emission decreased over time in both watering treatments. However, it was only significant in water-deficient plants between day 0 and day 7 (Post-hoc Tukey: 0d – 7d, *P* = 0.010).

#### Homoterpenes

Emission in *B. napus* decreased over time (LMM: *P* = 0.038; Fig. 4a, Table S12), with highest emission at day 0. Emission was twice as high with N supplementation compared with the treatment without N supplementation (LMM: *P* = 0.006). In *S. alba* plants, emission was affected by watering treatment and N supplementation as well as the interaction of time and watering. Water deficit and N supplementation decreased emission by more than half (LMM_Drought_: *P* = 0.009, LMM_N_: *P* = 0.004; Fig. 4b, Table S12). The interacting effect of time and watering resulted in an increase of homoterpene emission over time in the watering treatment, whereas emission decreased over time under water deficit (LMM: *P* = 0.033; Fig. 4b, Table S12). In *S. arvensis* plants we found an interacting effect of time and watering treatment (LMM: *P* = 0.006; Fig. 4c, Table S12), as the emission was significantly lower under water deficit at day 14 (Post-hoc Tukey: *P* = 0.033).

## Discussion

Single abiotic factors can affect floral scent emission in specific plant species (Burkle and Runyon 2016; Majetic et al. 2017; Glenny et al. 2018; Campbell et al. 2019); however, little is known about the effects of the interaction of abiotic factors on total floral scent emission and scent composition. An understanding of the plasticity of floral scent in crop species in response to several abiotic factors is especially important for the growing agricultural sector of flowering crops depending on pollinators. In this paper, we have shown, for three Brassicaceae species (cultivated species: *Brassica napus* & *Sinapis alba*, wild plant: *Sinapis arvensis*), the way that progressive water deficit interacts with N availability to affect emission rate and composition of floral scent. Overall, we have found that the scent emission of the cultivated *B. napus* and the wild *S. arvensis* is less sensitive to abiotic factors, whereas the cultivated *S. alba* is highly sensitive to changes in abiotic conditions.

### Stem Water Potential, Blooming, and Survival

The stem water potential revealed that *B. napus* and *S. alba* under water deficit showed pronounced drought stress, in contrast to *S. arvensis*; though, this result may be limited due to a reduced sample size for *S. arvensis* for the SWP measurement. Nevertheless, all plant individuals of all species under water deficit showed significant signs of wilting, indicating that they suffer from drought stress. Contrary to our expectation that an adequate N supply can enhance the tolerance of plants to water deficits (Quemada and Gabriel 2016; Akter and Klečka 2020), N supplementation had no (mitigating) effect on the SWP. However, N availability had a great impact on the stability and survival of *S. alba* plants until flowering, as stems of individuals starving N snapped off, indicating a low breaking resistance (Ye et al. 2016). Moreover, N seems to impact the flowering time, as *S. alba* plants with N supplementation flowering earlier, than plants without N supplementation (personal observation). Indeed, it was shown that N starvation down-regulates photosynthetic and macromolecular synthesis, causing severe growth retardation, which possibly explains delayed flowering (Lin and Tsay 2017; Weber and Burow 2018). On the other hand, in *Arabidopsis* and pea, low levels of nitrate accelerated flowering time but stopped flowering earlier (Jeuffroy and Sebillotte 1997; Castro Marín et al. 2011; Liu et al. 2013), which was the same for our plants without N supplementation, that stopped producing flowers after seven days. Water-deficient plants also stopped flowering earlier as a possible adaption to escape drought stress by accelerating flowering (Sherrard and Maherali 2006; Franks et al. 2007; Bernal et al. 2011; Franks 2011; Kazan and Lyons 2016). Most individuals for all species that produced flowers until the last day of scent collection (day 14) were in the watered treatment with N supplementation. The most individuals that bloomed until the end, independent of treatment, were *S. arvensis*. In summary, survival until flowering, and blooming time were very different among our focal species, indicating that species differ in their response to water deficit and N availability.

### Total Scent Production

The overall scent production per flower was lowest in *B. napus* compared with the other species. Together with the lowest total number of flowers of all three species, the overall scent emission in a single *B. napus* plant is therefore reduced. Since commercialized oilseed rape is grown in large monocultures, there might be no need for a single individual to produce a high number of flowers and large amounts of scent to attract pollinators, due to group scent and visual display. Furthermore, a reduced scent emission might reduce attractiveness for herbivores, as was shown for *B. napus* and *B. rapa*, where the former species produced lesser scent in the bud stage, leading to a reduced infestation of *B. napus* plants with pollen beetles *Meligethes aeneus* (Cook et al. 2007). Even though, insect pollination increases yield by more than 50% (Chiari et al. 2005; Araneda Durán et al. 2010), there is artificial selection to low emission rates due to detrimental effects of crop pest attraction, compared with the wild species *S. arvensis*. It is also possible that selection for reduced scent emission is not targeted, but that strong selection for higher seed yield comes at the expanse of floral traits, especially scent (Saunier and Blande 2019).

Treatment only had an effect on *B. napus*, with an increased scent emission under water deficit compared with the watered individuals, which was already shown in other species (Burkle and Runyon 2016; Glenny et al. 2018), suggesting that there could be widespread enhancement of this pollinator cue under the predicted drier climate. In both *Sinapis*-species, scent emission was not affected by treatments, indicating that scent emission remains unchanged under water deficit. However, drought is often accompanied by high temperatures, which are known to increase volatile emission (Farré-Armengol et al. 2013; Farré-Armengol et al. 2014). Thus, predicted increasing temperatures could cause floral scent emission to increase.

### Scent Bouquets, Compounds, and Treatments

All three species differed in their floral scent bouquets. In contrast to the expectation that scent bouquets of *S. arvensis* plants be the most variable given the variable environmental conditions under which it grows, the scent bouquets of *B. napus* showed the highest variation. As *B. napus* plants show a mixed-mating system and are not necessarily dependent on pollinators for reproduction (Blochtein et al. 2014), the emission of a specific scent bouquet for pollinator attraction possibly plays a subordinate role in this species. However, we did not control for maternal lines, and variations of scent bouquets might be due to unplanned differences in genetic diversity between plant individuals, rather than a result of phenotypic plasticity of an individual. Nevertheless, insect pollination increased seed production (Kevan and Eisikowitch 1990; Rosa et al. 2010; Ali et al. 2011; Blochtein et al. 2014), seed weight (Araneda Durán et al. 2010; Bommarco et al. 2012), and seed germination rate (Kevan and Eisikowitch 1990). *Sinapis alba* and *S. arvensis* are self-incompatible species (Olsson 1960; Ford and Kay 1985; Lucas-Barbosa 2016). Therefore, both rely on insect pollination (Flacher et al. 2020), which might explain why they emit more scent and have stable bouquets.

Floral scent bouquets of *B. napus* and *S. alba* changed over time. In *S. alba*, bouquets of watered and water-deficient plants significantly differed after one week, indicating that progressive water deficit induced stress in plants, which then affected floral scent production. Further, our results revealed that scent bouquets of *B. napus* and *S. alba* were affected by the interacting effect of watering- and N supplementation. While bouquets of watered and water-deficient plants were separated with N supplementation, the bouquets of watered and water-deficient plants did not differ without N supplementation. We would have expected the opposite, as N supplementation might mitigate water-deficient, so plants emit scent bouquets that are similar to plants in the watered treatment. It could be the case that N starvation has a such strong impact that even watered plants that suffer from N starvation, emit similar bouquets as water-deficient plants. Thus, N starvation and water-deficient might not act additive to floral scent.

For *S. arvensis* bouquets we found only an effect of watering treatment. In our previous study (Höfer et al. 2021), we found no effect of water deficit on floral scent emission for this species. However, in this former experiment, watering treatment started before the flowering stage and lasted for several weeks, before we took one-time scent samples. Short periods of water deficit without pulsed watering to the point of saturation at the beginning of flowering might have a stronger impact (Kuppler and Kotowska 2021). When water deficit is only effective for a short time, plants would not yet be able to maintain their scent bouquets by adjusting their physiology to compensate for abiotic stress, e.g., by reducing plant growth to allocate resources to the reproductive organs for the maintenance of scent production for attracting pollinators. Under long-term drought stress, the ability to restore ‘normal’ scent bouquet is a key ability, as pollinators can recognize quantitative and qualitative differences in floral scent (Andersson 2003; Andersson and Dobson 2003; Wright et al. 2005). Changes in the floral scent bouquet might lead to functional and recognition mismatches between host plants and flower visitors (Miller-Struttmann et al. 2015; Descamps et al. 2020; Gérard et al. 2020), as increases in volatile emissions might be a deterrent to pollinators that rely on specific scent to find their host plant, and hence negatively affecting pollinator visits (Junker and Blüthgen 2010) with consequences on plant’s reproductive success. *Sinapis arvensis* grows in highly disturbed environments such as meadows or agricultural landscapes and therefore might demonstrate high plasticity in floral scent. Therefore, the effects of a higher water deficit and N availability on floral scent might lie within the normal range of variation, making this species less sensitive to abiotic stressors.

The effect of treatments on compound emission was highly compound- and species-specific. Emission rates for monoterpenes were significantly reduced under water deficit in *S. alba*, whereas they were unaffected in *S. arvensis* and *B. napus*. These mixed results have previously been shown in various tree species in which foliar terpene emission decreases in the terpene-storing *Pinus halepensis* and increases in the non-storing *Quercus ilex* (Blanch et al. 2007). In sage (*Salvia officinalis*), the quantity of monoterpenes in leaves increases strongly under drought stress (Nowak et al. 2010). Monoterpenes are known to have a defensive role in vegetative tissues (Dudareva and Pichersky 2000) and serve as antioxidants by scavenging reactive oxygen species (González-Burgos and Gómez-Serranillos 2012), which are produced under abiotic stressors, e.g., elevated ozone, high temperature, intense light, or drought stress (Aro et al. 1993; Kozaki and Takeba 1996; Peñuelas and Llusià 2002; Saunier and Blande 2019). Nevertheless, in the common weed *S. arvensis*, the emission of monoterpenes by its flowers might not have been increased but might be maintained under drought stress, because it accumulates and is used in tissues potentially to protect cells from reactive oxygen species, and because it stabilizes membranes (Peñuelas and Llusià 2002). This might be the reason that the stem water potential (SWP) of watered and water-deficient plants did not differ, as the monoterpenes stabilize and maintain cell turgor. In *S. alba*, we found a significant reduction in SWP under water deficit, indicating this species might be unable to maintain cell turgor through increased monoterpene accumulation. The reduced emission of those compounds is therefore more likely to be attributable to the down-regulation of monoterpene synthases to reduce metabolic costs (Farré-Armengol et al. 2014). Measurement of SWP in *B. napus* revealed a strong impact of water deficit on the water potential, indicating that this highly cultivated species might not be able to compensate for water deficit and to maintain cell turgor. However, the potential mechanisms of monoterpenes on water-use efficiency need further investigations. The impacts of reduced monoterpene emission on flower visitors should be further investigated, as degraded floral scent and also changes in the ratio of compounds in a scent bouquet due to exposure to ozone led to reduced attractiveness to pollinators (Farré-Armengol et al. 2016).

In contrast to Veromann et al. (2013), we found no increased emission of Methyl salicylate, produced by Shikimate pathway, with N supplementation. However, this might be attributable to differences in the amount of applied N. Following previous studies (Dudareva and Pichersky 2000; Majetic et al. 2008; Majetic et al. 2017), the emission of aromatics, such as the N-containing compound Indole or of substances that are synthesized from the N-containing Phenylalanine (e.g. Benzaldehyde) were increased with N supplementation; however, we found this effect only for the *S. alba* plants. The emission of fatty-acid derivatives were not affected by N supplementation in any of the species, as these compounds are not derived from N-containing compounds (Dudareva and Pichersky 2000; Majetic et al. 2008).

General scent emission of compounds was unaffected by treatments, indicating that pollinator attraction is still ensured. Moreover, these species attract a broad range of generalist insect species, such as hoverflies and honeybees, for which modifications of emission rates for specific compounds might not lead to recognition mismatches between them and their host plants. However, for specialized (oligolectic) pollinator species that use species-specific olfactory cues to find their host-species (e.g., Burger et al. 2010), modifications of emission rates would have detrimental consequences.

In summary, we found that scent bouquets and emission of single compounds are less affected by environmental factors in *B. napus* compared with the other species. It might have been artificially selected to withstand decreased water availability and to maintain its normal phenotype and for increased seed yield; total scent emission might be reduced due to a possible trade-off between pollinator attraction and pest deterrence to increase yield production in this important crop species. In contrast, the second cultivated plant species *S. alba*, might be less artificially selected to withstand changes in water and nutrient availability and therefore, scent bouquets and single compounds changed in response to our treatments. In *S. arvensis*, scent bouquets and single compound emission showed weak responses to treatments indicating that this species has been naturally selected, during its evolution, to withstand decreased water availability and maintain its normal phenotype.

### Conclusion

Overall, we found that the response to changes in environmental factors, such as water and nutrient availability, is highly species-specific, even within species from the same family/genus. Furthermore, we found that the emission of single compounds in response to environmental factors varied between species and is highly compound-specific. Nonetheless, our study revealed that especially terpenes were negatively affected by water deficit. Further, as plants age, the scent emission decreases, and composition changes. The questions that arise now are what are the consequences if whole plant communities are under stress and how does this affect the plant-pollinator interactions?

## Supplementary Information

Below is the link to the electronic supplementary material.

ESM 1 (CSV 6.96 KB)

ESM 2 (CSV 280 bytes)

ESM 3 (CSV 2.77 KB)

ESM 4 (CSV 1.59 KB)

ESM 5 (CSV 149 bytes)

ESM 6 (CSV 1.07 KB)

ESM 7 (TXT 4.87 KB)

ESM 8 (CSV 4.87 KB)

ESM 9 (R 109 KB)

ESM 10 (CSV 372 KB)

ESM 11 (DOCX 1.16 MB)

## Data Availability

Data and R-Code used in this paper are available in the electronical supplementary material (Data: ESM 1-6, 8, 10; Readme: ESM 7; R-Code: ESM 9).
