# Supplementary material for: Water Deficit, Nitrogen Availability, and Their Combination Differently Affect Floral Scent Emission in Three Brassicaceae Species
Source: J Chem Ecol. 2022 Dec 16;48(11-12):882–99. doi: 10.1007/s10886-022-01393-z (PMC9840598; doi:10.1007/s10886-022-01393-z)
Supplement: Supplementary file 11 — (DOCX 1.16 MB) [file 10886_2022_1393_MOESM11_ESM.docx]

**Supplementary Information**

Water Deficit, Nitrogen Availability and their combination differently affect Floral Scent Emission in Three Brassicaceae Species

Rebecca J. Höfer^1*^, Manfred Ayasse^1^, Jonas Kuppler^1^

*^1^Institute of Evolutionary Ecology and Conservation Genomics, Ulm University, Ulm, Germany*

*****For correspondence: rebecca.hoefer@uni-ulm.de

Contents:

**Notes S1 Survival/Mortality of plants until flowering**

**Notes S2 Scent bouquet species day 0, N-treatment**

**Notes S3 Flower traits**

**Supplemental Figures** S1

**Supplemental Tables** S1-S9

# Notes S1 Survival/Mortality of plants until flowering

We found that the mortality rate until flowering of individuals in the no-Nitrogen treatment among all species was at 64% (Table I) and was significantly higher compared to individuals in the N+ treatment (t_4_ = 3.20, *P* = 0.033).

Especially individuals of *S. alba* in the no-Nitrogen treatment had very thin stems and and easily broke despite providing them some stabilization. For *S. arvensis* we observed similar. It was shown for maize (*Zea mays* L.) that dry weight, internode diameter and breaking resistance improved with increasing N rate (Ye et al. 2016). Many of *B. napus* plant individuals in the no-Nitrogen treatment grew very slow and did not produce floral buds until the end of the experiment (included in ‘Mortality’ data).

**Table SI** Survival of plant individuals in no-Nitrogen (N-) and Nitrogen (N+)-treatment until flowering

| **Species** | **Treatment** | **Start**  **#** | **Survival**  **#** | **Mortality**  **#** | **Survrate %** | **Mortrate %** |
| --- | --- | --- | --- | --- | --- | --- |
| *B. napus* | N- | 30 | 14 | 16 | 46.7 | 53.3 |
| *B. napus* | N+ | 30 | 22 | 8 | 73.3 | 26.7 |
| *S. alba* | N- | 30 | 6 | 24 | 20.0 | 80.0 |
| *S. alba* | N+ | 30 | 20 | 10 | 66.7 | 33.3 |
| *S. arvensis* | N- | 30 | 12 | 18 | 40.0 | 60.0 |
| *S. arvensis* | N+ | 30 | 17 | 13 | 56.7 | 43.3 |

## References

Ye DL, Zhang YS, Al-Kaisi MM, Duan LS, Zhang MC, and Li ZH (2016) Ethephon improved stalk strength associated with summer maize adaptations to environments differing in nitrogen availability in the North China Plain. J Agric Sci 154:960–977. https://doi.org/10.1017/S0021859615000829j

# Notes S2 Scent bouquet of species at day 0 in the N-treatment

To detect differences between the three plant species at day 0 for treatments N- Watered and N- Drought, we performed permutational analyses (PERMANOVA) with the Bray-Curtis similarity distance matrix by using the *adonis*()-function (9999 permutations) from the vegan-package 2.5-7 (Oksanen et al. 2020). The *envfit*()-function from the vegan-package was used to extract the scent compounds and class of compounds that mainly drove the differences between the scent bouquets of the plant species (*P.max* = 0.01). For a pair-wise comparison between species, we employed the *pairwise.adonis*()-function from the *pairwiseAdonis*-package 0.4 (Martinez Arbizu 2020). To calculate the multivariate dispersion for each species, we first calculated dissimilarity indices with the Bray-Curtis similarity distance matrix by using the *vegdist*()-function from the *vegan*-package. To test for differences in scent variability (dispersion; the average distance from the group centroid), we performed a permutational test for homogeneity of multivariate dispersion by using the *betadisper*()-function from the vegan-package. The *anova*()-function from the vegan-package with 9999 permutations was employed to test for significance of multivariate dispersion between species. Subsequently, the Tukey-test was performed using the *TukeyHSD*()-function from the stats-package in order to check for pairwise differences of group dispersions between the species. We used non-metric multidimensional scaling (NMDS) for ordination in order to depict differences in the scent bouquet between the species graphically. The *metaMDS*()-function from the vegan-package was employed to run the NMDS, with the Bray-Curtis distance matrix and 9999 permutations.

Result R1 scent bouquets at day 0 and N-

*Scent Emission of the Tested Species.* The NMDS graph of the scent bouquet at day 0 for the N- treatments revealed that all three species differed significantly in their scent composition (Fig. I, Table I; Pair-wise comparison: *Padj*= 0.003 for all comparisons, Table Ib). Calculation of the multivariate dispersion showed that scent bouquets of *B. napus* plants were more variable than that of *S. alba* and *S. arvensis* (Post-hoc Tukey: *B. napus* – *S. alba Padj*= 0.004; *B. napus* – *S. arvensis Padj* < 0.001; *S. alba* – *S. arvensis* *Padj*= 0.517, see details in Table Id). Further, we found that the scent bouquet of *B. napus* was mainly characterized by fatty-acid-derivates and aromatics, whereas *S. alba* and *S. arvensis* were mainly characterized by mono- and sesquiterpenes (Fig. I).


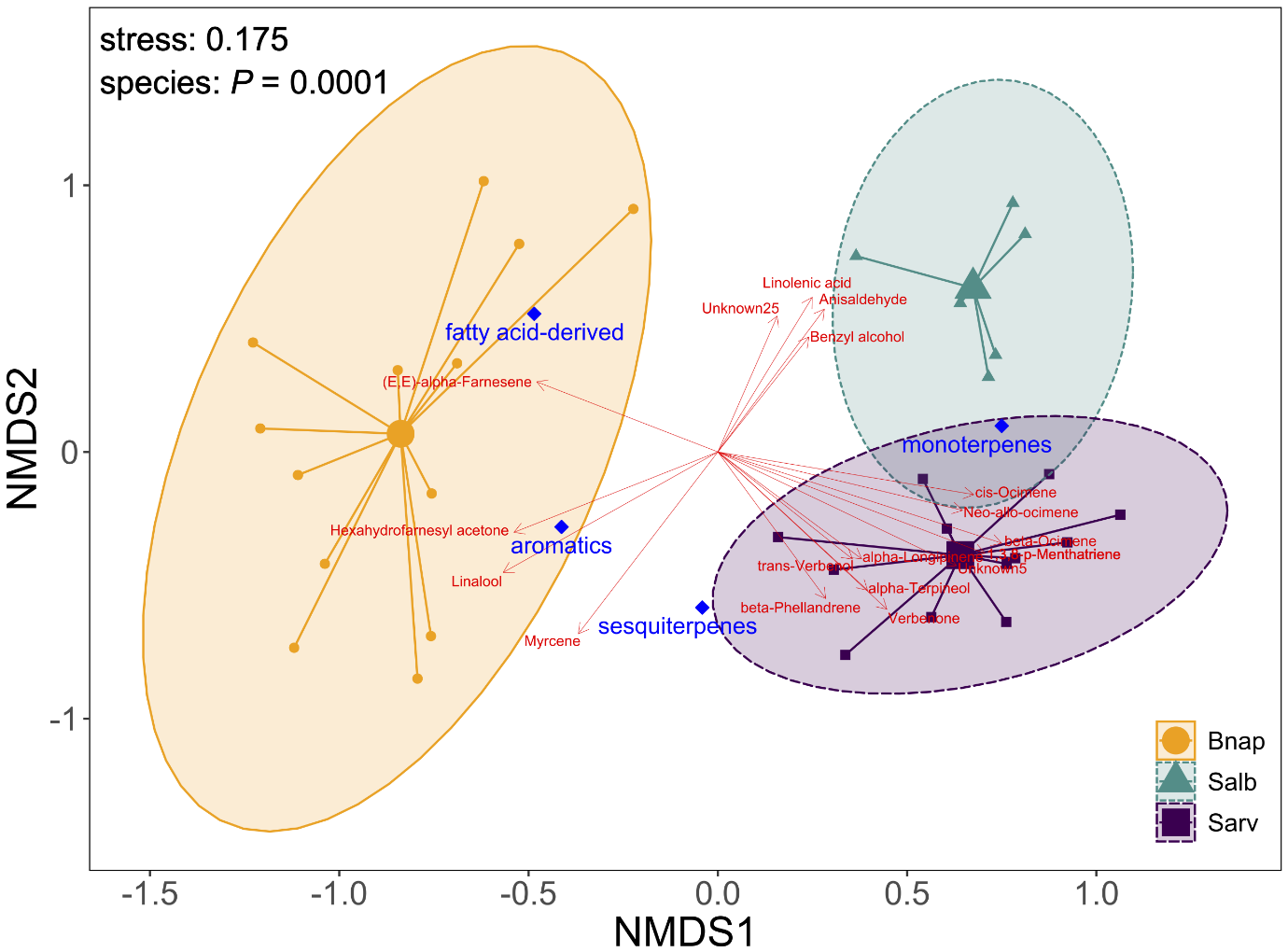


**Fig. I** NMDS (non-metric multidimensional scaling) plot projected in two-dimensional space based on Bray-Curtis distances (2D stress = 0.175) of scent composition at day 0 for treatments N- Watered and N- Drought together. Colored symbols represent separate species: *B.* *napus* (yellow circles): *n* = 14; *S.* *alba* (green triangles): *n* = 6; *S. arvensis* (purple rectangles): *n* = 12. Each small colored symbol shows the scent sample of an individual; the three larger symbols show centroids for each species. Each red arrow represents scent compounds, and each blue diamond the class of compounds most responsible for variation in floral scent between species. Colored ellipses illustrate the confidence level of 95 % for the *t*-distribution for each species. Scent composition is significantly affected by species (PERMANOVA: *R^2^* = 0.407, *P* = 0.0001)

**Table II** Results of Permutational multivariate analysis of variance (*adonis* function) and multivariate homogeneity of group dispersion analysis (*betadisper* function) for scent bouquets of the three species *B. napus* (Bnap), *S. alba* (Salb), *S. arvensis* (Sarv). Tests are based on Bray-Curtis dissimilarity distances and 9999 permutations. Signiﬁcant p-values at *P* ≤ 0.05 are highlighted in bold.

1. Permutational multivariate analysis of variance (PERMANOVA) between the different species.

| **Source** | **Df** | **Sums Sq** | **Mean Sq** | **F Model** | ***R^2^*** | ***P*** |
| --- | --- | --- | --- | --- | --- | --- |
| Groups | 2 | 4.02 | 2.01 | 9.96 | 0.41 | **0.0001** |
| Residuals | 29 | 5.86 | 0.20 |  | 0.59 |  |
| Total | 31 | 9.88 |  |  | 1.00 |  |

1. Pairwise analysis of variance between the different species.

| **Pairs** | **Df** | **Sums Sq** | **F Model** | ***R^2^*** | ***P*** | ***P* adjusted** |
| --- | --- | --- | --- | --- | --- | --- |
| Bnap × Salb | 1 | 1.66 | 6.69 | 0.27 | 0.001 | **0.003** |
| Bnap × Sarv | 1 | 2.71 | 12.97 | 0.35 | 0.001 | **0.003** |
| Salb × Sarv | 1 | 1.38 | 9.96 | 0.38 | 0.001 | **0.003** |

1. Multivariate homogeneity of group dispersions analysis between the different species.

| **Factor** | **Df** | **Sums Sq** | **Mean Sq** | ***F* Model** | ***P*** |
| --- | --- | --- | --- | --- | --- |
| Groups | 2 | 0.22 | 0.11 | 17.08 | **<0.001** |
| Residuals | 29 | 0.19 | 0.01 |  |  |

1. Pairwise analysis of multivariate homogeneity of group dispersions between the different species.

| **Pairs** | **diff** | **lwr** | **upr** | ***P* adjusted** | **Distance to**  **centroid** |
| --- | --- | --- | --- | --- | --- |
| Salb - Bnap | -0.14 | -0.23 | -0.04 | **0.004** | Bnap: 0.506 |
| Sarv - Bnap | -0.18 | -0.26 | -0.10 | **<0.0001** | Salb: 0.368 |
| Sarv - Salb | -0.04 | -0.14 | 0.06 | 0.562 | Sarv: 0.326 |

# Notes S3 Flowers

Method S3.1 Sum of produced flowers

To investigate how watering- and Nitrogen-treatment affect the overall produced flowers during the flowering period of each plant species, we counted all open and fresh flowers each sampling day for each plant individual (0d, 2d, 7d, 14d) and summarized it to the sum of flowers. For statistical analysis, we performed linear models by using the *lm*()-function from the stats-package 4.1.0 (R Core Team 2021) for each species, with sum of flowers as the dependent factor and with treatments as fixed factors. To explore differences in the overall produced flower number between the three species, we performed linear models with sum of flowers as the dependent factor and with species as fixed factors. The *Anova*() function was used to test for significance. The assumptions of all models were assessed using the *DHARMa*-package 0.4.1 and were met (Hartig 2020).

Results R3.1 Sum of produced flowers

For *B. napus* and *S. arvensis*, we found no effect of treatments on the number of produced flower (Table II). For *S. alba* plants, we found that treatment significantly affected flower number (Table II). Drought-stressed plants produced 1.5 times less flowers compared to watered plants (LM: *F*_1,22_ = 5.39, *P* = 0.030; data not shown), and plants without nitrogen produced half the flowers compared to plants with nitrogen (LM: *F*_1,22_ = 11.56, *P* = 0.003; data not shown). Overall, *B. napus* plants produced the fewest number of flowers and *S. arvensis* plants most flowers, with all treatments taken together.

**Table SIII** Mean (± SD) of sum flowers produced over entire flowering period for each plant species under the four treatments

| **Species** |  | **Treatment** | | |  |
| --- | --- | --- | --- | --- | --- |
|  | Mean ± SD | N-Control | N-Drought | N+Control | N+Drought |
| *B. napus* | 18.6 ± 7.7A^a^ | 18.3 ± 7.7a | 13.4 ± 5.4a | 21.2 ± 8.6a | 19 ± 7.5a |
| *S. alba* | 56.5 ± 33.1B | 42 ± 29.6ab | 21.7 ± 11.a | 75.9 ± 33.2b | 52 ± 28.ab |
| *S. arvensis* | 66.7 ± 29.5B | 76 ± 35.2a | 60.7 ± 20.9a | 73.1 ± 30.9a | 56.98 ± 30.2a |

Capital letters denote significant differences between the means of species, while small letters denote significant treatment differences within the species.

^a^ Means were compared by ANOVA

Method S3.2 Flower fresh weight

To explore how treatments affect the flower weight, we collected all flowers of a plant after the last scent collection at day 14, provided that the plants have produced flowers by then. Flowers were collected without the pedicel, but with sepals and were weighed directly afterwards (pooled for each plant individual). Afterwards, the weight per flower was calculated (pooled weight divived by collected flower number). For *B. napus*, as no individual under drought stress flowered until day 14, we could not collect flowers for this treatment. For statistical analysis, we performed linear models models by using the *lm*()-function from the stats-package 4.1.0 for each species, with floral fresh weight as the dependet factor and treatments as the fixed factors. The *Anova*() function was used to test for significance. The assumptions of all models were assessed using the *DHARMa*-package 0.4.1 and were met.

Results R3.2 Flower fresh weight

For *B. napus*, we found no effect of treatment on floral fresh weight; however, we were able to collect flowers only from 4 plant individuals. For *S. alba*, treatment has also no effect on floral weight. For *S. arvensis*, we found that flowers in the drought-stress treatment were half lighter than flowers from plants in the watered treatment (LM: *F*_1,11_ = 15.63, *P* = 0.002; see Table III for means).

**Table SIV** Mean (± SD) g of fresh weight per flower for each plant species under the treatments

| **Species** |  | **Treatment** | | |  |
| --- | --- | --- | --- | --- | --- |
|  | Mean ± SD | N- | N+ | Watered | Drought |
| *B. napus* | 0.011 ± 0.004A^a^ | 0.012 ± NAa | 0.011 ± 0.005a |  |  |
| *S. alba* | 0.017 ± 0.010A |  |  | 0.016 ± 0.008a | 0.019 ± 0.014a |
| *S. arvensis* | 0.015 ± 0.006A |  |  | 0.019 ± 0.005a | 0.010 ± 0.003b |

Capital letters denote significant differences between the means of species, while small letters denote significant treatment differences within the species.

^a^ Means were compared by ANOVA

# Supplemental Figures


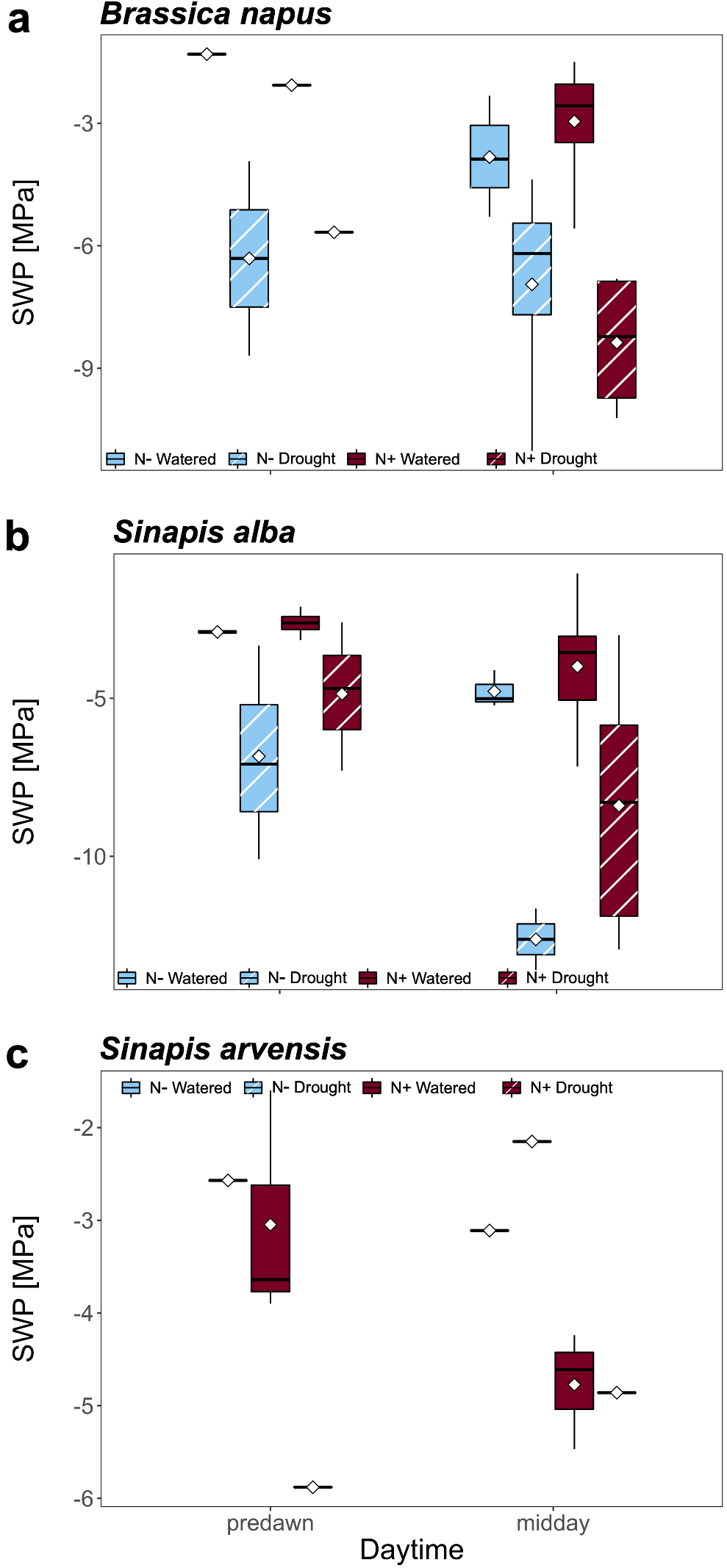


**Fig. S1** Stem water potential (SWP in MPa) for the plant species measured predawn and at midday. Blue color shows treatment without additional nitrogen. Red color shows treatment with additional nitrogen; white stripes show drought stress treatment.

(**A**) *B. napus*: predawn: *n* = 5 (N- Watered *n* = 1, N- Drought *n* = 2, N+ Watered *n* = 1, N+ Drought *n* = 1); midday: *n* = 21 (N- Watered *n* = 6, N- Drought *n* = 4, N+ Watered *n* = 7, N+ Drought *n* = 4).

(**B**) *S. alba*: predawn: *n* = 12 (N- Watered *n* = 2, N- Drought *n* = 3, N+ Watered *n* = 4, N+ Drought *n* = 3); midday: *n* = 17 (N- Watered *n* = 3, N- Drought *n* = 2, N+ Watered *n* = 7, N+ Drought *n* = 5).

(**C**) *S. arvensis*: predawn: *n* = 5 (N- Watered *n* = 1, N- Drought *n* = 0, N+ Watered *n* = 3, N+ Drought *n* = 1); midday: *n* = 6 (N- Watered *n* = 1, N- Drought *n* = 1, N+ Watered *n* = 3, N+ Drought *n* = 1).

Boxplots show median range, 25th and 75th percentile interquartile range. Whiskers show 1.5x interquartile range. White diamonds show mean values.

# Supplemental **Tables**

**Table S1** Relative amount (mean ± SE with *n* = sample size) of volatile compounds in headspace samples of flowers of *B. napus*, *S. alba* and *S. arvensis* at different time points (day 0 - day 14) under four treatments (N- C = watered without nitrogen, N- D = drought without nitrogen, N+ C = watered with nitrogen, N+ D = drought with nitrogen). Retention index (RI) is given for each compound. Compounds are grouped in compound classes. ‘-‘ indicates compounds, that were not found in samples. For compound identification we used three criteria: a = using authentic standards, b = RI and mass spectra consistent with values in Adams°2007, NIST Webbook or Pherobase, and c = high MS library match (>91%; if no standard was available and RI does not match published values). The compounds that we found in the flowers were compared with those found in blank controls (empty oven bags and samples of green leaves) and were checked using ‘The Pherobase’ to determine those compounds that were emitted in particular by flowers

|  | | ***Brassica napus*** | | | | | | | | | | | | | | | |
| --- | --- | --- | --- | --- | --- | --- | --- | --- | --- | --- | --- | --- | --- | --- | --- | --- | --- |
|  |  | 0 d | | | | 2 d | | | | 7 d | | | | 14 d | | | |
| Compounds | RI | N- C | N- D | N+ C | N+ D | N- C | N- D | N+ C | N+ D | N- C | N- D | N+ C | N+ D | N- C | N- D | N+ C | N+D |
| ***Aliphatics*** |  |  |  |  |  |  |  |  |  |  |  |  |  |  |  |  |  |
| 3-Octanone**^a^** | 985 | - | - | - | - | - | - | - | - | - | - | - | - | - | - | - | - |
| 2-Undecanone**^b^** | 1293 | - | - | - | - | - | - | - | - | - | - | - | - | - | - | - | - |
| 1-Tetradecene**^b^** | 1392 | 0.01 ±  0  *1* | - | - | 0.01 ±  0  *1* | - | - | - | - | - | - | - | - | 0.07 ±  0  *1* | - | - | - |
| 2-Pentadecanone**^a^** | 1698 | - | - | - | 0.01 ±  0  *1* | - | 0.07 ±  0  *1* | - | - | - | - | - | - | - | - | - | - |
|  |  |  |  |  |  |  |  |  |  |  |  |  |  |  |  |  |  |
| ***Aromatics*** |  |  |  |  |  |  |  |  |  |  |  |  |  |  |  |  |  |
| 2-Furanmethanol**^a^** | 859 | - | - | - | - | - | - | - | - | - | - | - | - | - | - | - | - |
| 1,2-Cyclopentanedione**^b^** | 923 | - | - | - | - | - | - | 0.01 ±  0  *1* | - | - | - | - | 0.01 ±  0  *1* | - | - | - | - |
| Benzaldehyde**^a^** | 962 | 0.11 ±  0  *1* | 0.05 ±  0  *1* | 0.14 ±  0.07  *3* | 0.05 ±  0.01  *4* | 0.06 ±  0  *1* | 0.08 ±  0.06  *2* | 0.12 ±  0.06  *2* | 0.29 ±  0.20  *3* | 0.10 ±  0  *1* | - | 0.06 ±  0  *1* | 0.10 ±  0.03  *4* | - | - | 0.06 ±  0  *1* | - |
| Benzyl alcohol**^a^** | 1033 | - | - | 0.03 ±  0.02  *2* | 0.02 ±  0.004  *2* | 0.12 ±  0.09  *2* | 0.10 ±  0.08  *3* | 0.01 ±  0  *1* | 0.07 ±  0.05  *2* | 0.06 ±  0  *1* | - | - | 0.04 ±  0.01  *3* | - | - | - | - |
| Phenylacetaldehyde**^a^** | 1042 | - | - | - | - | - | 0.01 ±  0  *1* | - | - | 0.11 ±  0  *1* | - | - | - | - | - | - | - |
| Acetophenone**^a^** | 1065 | 0.06 ±  0  *1* | 0.01 ±  0.001  *2* | 0.12 ±  0  *1* | 0.02 ±  0.01  *3* | 0.03 ±  0  *1* | 0.01 ±  0  *1* | - | 0.07 ±  0.05  *2* | - | - | 0.07 ±  0  *1* | 0.06 ±  0.02  *3* | - | - | - | - |
| p-Cymenene**^b^** | 1090 | - | - | - | - | - | - | - | - | - | - | - | - | - | - | - | - |
| Methyl benzoate**^a^** | 1096 | 0.03 ±  0  *1* | 0.04 ±  0.01  *2* | 0.05 ±  0.02  *3* | 0.02 ±  0  1 | 0.19 ±  0.07  *2* | 0.09 ±  0.09  *2* | 0.03 ±  0  *1* | 0.08 ±  0  *1* | - | - | 0.03 ±  0  *1* | - | - | - | - | - |
| 2-Phenylethanol**^a^** | 1118 | - | - | - | - | - | 0.07 ±  0  *1* | - | 0.03 ±  0  *1* | 0.05 ±  0  *1* | - | - | - | - | - | - | - |
| Benzyl cyanide**^a^** | 1136 | - | - | - | - | - | - | - | - | - | - | - | - | - | - | - | - |
| Benzyl acetate**^a^** | 1163 | - | - | - | - | - | - | - | - | - | - | - | - | - | - | - | - |
| Methyl salicylate**^a^** | 1191 | - | 0.11 ±  0.06  *3* | 0.04 ±  0.02  *5* | 0.01 ±  0.001  *2* | 0.03 ±  0  *1* | 0.02 ±  0  *1* | 0.03 ±  0.01  *5* | 0.01 ±  0.003  *4* | 0.05 ±  0  *1* | - | 0.01 ±  0  *1* | 0.01 ±  0  *1* | - | - | - | - |
| Coumaran**^b^** | 1217 | - | - | - | - | - | - | - | - | - | - | - | - | - | - | - | - |
| 2-Aminobenzaldehyde**^a^** | 1220 | - | - | - | - | - | - | - | - | - | - | - | - | - | - | - | - |
| p-Anisaldehyde**^a^** | 1261 | - | - | 0.03 ±  0  *1* | 0.02 ±  0.01  *2* | - | 0.01 ±  0  *1* | - | - | - | - | - | - | - | - | - | - |
| p-Anisyl alcohol**^b^** | 1287 | - | - | - | - | - | - | - | - | - | - | - | - | - | - | - | - |
| Indole**^a^** | 1292 | 0.08 ±  0.02  *4* | 0.09 ±  0.04  *3* | 0.07 ±  0.01  *3* | 0.05 ±  0.02  *4* | 0.23 ±  0.05  *2* | 0.22 ±  0  *1* | 0.06 ±  0.02  *4* | 0.07 ±  0.03  *4* | 0.27 ±  0  *1* | - | - | - | - | - | - | - |
| 1-Nitro-2-phenylethane**^b^** | 1301 | - | - | - | - | - | - | - | - | - | - | - | - | - | - | - | - |
| Benzyl mustard oil**^b^** | 1364 | - | - | - | - | - | - | - | - | - | - | - | - | - | - | - | - |
| Methyl vanillin**^b^** | 1481 | - | - | - | - | - | - | - | - | - | - | - | - | - | - | - | - |
|  |  |  |  |  |  |  |  |  |  |  |  |  |  |  |  |  |  |
| ***Fatty acid-derived*** |  |  |  |  |  |  |  |  |  |  |  |  |  |  |  |  |  |
| (*Z*)*-*3-Hexenol**^a^** | 861 | - | - | - | - | - | - | - | - | - | - | - | - | - | - | - | - |
| Heptanal**^a^** | 903 | - | 0.01 ±  0.004  *2* | 0.03 ±  0  *1* | 0.01 ±  0  *1* | 0.02 ±  0  *1* | 0.03 ±  0.03  *2* | 0.01 ±  0  *1* | 0.01 ±  0  *1* | 0.04 ±  0.02  *2* | - | - | 0.06 ±  0.02  *4* | - | - | - | - |
| Amyl acetate**^b^** | 912 | - | - | - | - | - | - | - | - | - | - | - | - | - | - | - | - |
| (*E*)-2-Heptenal**^b^** | 957 | - | - | - | - | - | - | - | - | 0.02 ± 0  *1* | - | - | - | - | - | - | - |
| (*Z*)-1,5-Octadien-3-ol**^c^**  ^match 92%^ | 973 | - | - | - | - | - | - | - | - | - | - | 0.02 ± 0  *1* | - | - | - | 0.06 ± 0  *1* | - |
| 1-Octen-3-ol**^a^** | 980 | - | - | - | - | - | - | - | - | - | - | - | - | - | - | - | - |
| Octanal**^a^** | 1003 | - | 0.02 ±  0  *1* | - | 0.08 ±  0  *1* | - | 0.02 ±  0  *1* | 0.08 ±  0  *1* | 0.06 ±  0.01  *2* | 0.04 ±  0  *1* | - | 0.03 ±  0  *1* | 0.23 ±  0  *1* | - | - | - | - |
| (3*Z*)-3-Hexenyl acetate**^a^** | 1005 | 0.25 ±  0.15  *3* | 0.14 ±  0.08  *4* | 0.16 ±  0.08  *8* | 0.13 ±  0.04  *5* | 0.15 ±  0.09  *4* | 0.15 ±  0.07  *4* | 0.10 ±  0.02  *7* | 0.17 ±  0.04  *6* | 0.09 ±  0.04  *4* | 0.05 ±  0  *1* | 0.18 ±  0.06  *8* | 0.06 ±  0  *1* | - | - | 0.16 ±  0.02  *3* | - |
| Hexyl acetate**^a^** | 1012 | 0.03 ±  0  *1* | 0.01 ±  0  *1* | 0.01 ±  0.003  *3* | 0.02 ±  0  *1* | 0.05 ±  0  *1* | 0.05 ±  0  *1* | - | - | 0.02 ±  0  *1* | - | 0.02 ±  0.01  *4* | - | - | - | - | - |
| Nonanal**^a^** | 1105 | 0.04 ±  0  *1* | 0.24 ±  0.13  *2* | 0.35 ±  0  *1* | 0.12 ±  0.03  *2* | 0.21 ±  0.03  *2* | 0.14 ±  0  *1* | 0.14 ±  0.01  *2* | 0.11 ±  0  *1* | 0.17 ±  0  *1* | - | 0.13 ±  0.08  *2* | 0.40 ±  0.20  *2* | - | - | 0.14 ±  0  *1* | - |
| (*Z*3)-Hexenyl butanoate**^b^** | 1142 | - | - | - | - | - | - | - | - | - | - | - | - | - | - | - | - |
| (2*E*, *Z*6)-Nonadienal**^b^** | 1154 | - | - | - | - | - | - | - | - | 0.01 ±  0  *1* | - | - | - | - | - | - | - |
| p-Mentha-1,5-dien-8-ol**^b^** | 1173 | - | - | - | - | - | - | - | - | - | - | - | - | - | - | - | - |
| Octanoic acid**^a^** | 1183 | - | - | - | - | - | 0.11 ±  0  *1* | - | - | 0.01 ±  0  *1* | - | - | - | - | - | - | - |
| Decanal**^a^** | 1207 | 0.05 ±  0  *1* | 0.11 ±  0  *1* | - | 0.29 ±  0.21  *2* | 0.41 ±  0  *1* | 0.08 ±  0  *1* | 0.39 ±  0  *1* | 0.25 ±  0  *1* | 0.25 ±  0  *1* | - | 0.22 ±  0.18  *2* | - | - | - | 0.23 ±  0  *1* | - |
| (*Z*)-3-Hexenyl-  2-methylbutanoate**^a^** | 1230 | - | - | - | - | - | - | - | - | - | - | - | - | - | - | - | - |
| (*Z*3)-Hexenyl- isovalerate**^a^** | 1236 | - | - | - | 0.02 ±  0  *1* | - | - | - | - | - | - | - | - | - | - | - | - |
| Undecanal**^a^** | 1308 | - | - | - | 0.02 ±  0  *1* | - | - | - | - | 0.03 ±  0  *1* | - | - | - | - | - | - | - |
| Decanoic acid**^a^** | 1371 | - | - | - | - | - | 0.07 ±  0  *1* | - | - | 0.03 ±  0  *1* | - | - | - | - | - | - | - |
| Dodecanal**^a^** | 1410 | - | - | - | 0.02 ±  0.01  *2* | - | - | - | - | 0.02 ±  0  *1* | - | - | - | - | - | - | - |
| 1-Dodecanol**^b^** | 1475 | - | - | - | 0.02 ±  0.001  *2* | - | - | - | - | - | - | - | - | - | - | - | - |
| 1-Pentadecene**^b^** | 1492 | - | 0.01 ±  0.001  *2* | 0.04 ±  0.02  *3* | 0.02 ±  0  *1* | 0.38 ±  0  *1* | 0.38 ±  0.21  *2* | - | - | 0.04 ±  0  *1* | - | 0.03 ±  0  *1* | 0.17 ±  0  *1* | 0.37 ± 0  *1* | - | - | - |
| Tridecanal**^a^** | 1511 | - | - | - | 0.004 ±  0  *1* | - | - | - | - | - | - | - | - | 0.08 ± 0  *1* | - | - | - |
| Myristic aldehyde**^a^** | 1613 | 0.02 ±  0  *1* | - | 0.02 ±  0  *1* | 0.01 ±  0  *1* | 0.10 ±  0  *1* | - | - | - | - | - | - | - | - | - | - | - |
| 1-Tetradecanol**^a^** | 1677 | - | - | - | 0.01 ±  0  *1* | - | - | - | - | - | - | - | - | - | - | - | - |
| Methyl myristate**^b^** | 1724 | 0.04 ±  0  *1* | - | - | - | - | - | - | - | - | - | - | 0.03 ±  0  *1* | - | - | - | - |
| Myristic acid**^a^** | 1757 | - | - | - | 0.06 ±  0.002  *2* | - | - | - | - | - | - | 0.03 ±  0  *1* | - | - | - | - | - |
| Methyl  pentadecanoate**^b^** | 1824 | - | - | - | - | - | - | - | - | - | - | - | 0.02 ±  0  *1* | - | - | - | - |
| Pentadecanoic acid**^b^** | 1863 | - | - | - | 0.04 ±  0.01  *2* | - | - | - | - | - | - | 0.02 ±  0  *1* | - | - | - | - | - |
| Methyl palmitate**^a^** | 1925 | 0.21 ±  0  *1* | - | - | - | - | - | - | - | 0.08 ±  0  *1* | - | 0.04 ±  0  *1* | 0.21 ±  0  *1* | - | - | - | - |
| Hexadecanoic acid**^a^** | 1974 | 0.51 ±  0  *1* | 0.06 ±  0.02  *2* | - | 0.30 ±  0.11  *4* | 0.21 ±  0  *1* | 0.11 ±  0  *1* | - | 0.25 ±  0  *1* | 0.05 ±  0  *1* | - | 0.23 ±  0.12  *3* | 0.37 ±  0  *1* | - | - | - | - |
| Methyl stearate**^b^** | 2125 | 0.08 ±  0  *1* | - | - | - | - | - | - | - | 0.10 ±  0  *1* | - | 0.02 ±  0  *1* | 0.09 ±  0  *1* | - | - | - | - |
| Linolenic acid**^a^** | 2146 | 0.12 ±  0  *1* | - | - | - | - | 0.05 ±  0  *1* | - | 0.03 ±  0  *1* | - | - | - | 0.02 ±  0  *1* | - | - | - | - |
| Octadecanoic acid**^a^** | 2168 | - | - | - | 0.10 ±  0.05  *2* | - | 0.03 ±  0  *1* | - | 0.05 ±  0  *1* | - | - | 0.07 ±  0  *1* | 0.08±  0  *1* | - | - | - | - |
| Oleamide**^c^** | 2179 | - | - | - | - | - | - | - | - | - | - | - | 0.01 ± 0  *1* | - | - | - | - |
|  |  |  |  |  |  |  |  |  |  |  |  |  |  |  |  |  |  |
| ***Monoterpenes*** |  |  |  |  |  |  |  |  |  |  |  |  |  |  |  |  |  |
| α-Thujene**^b^** | 924 | - | - | - | 0.01 ±  0  *1* | - | - | - | 0.02 ±  0.01  *2* | 0.03 ±  0  *1* | - | - | 0.02 ±  0  *1* | - | - | - | - |
| α-Pinene**^a^** | 932 | - | - | - | - | - | - | - | 0.004±  0  *1* | - | - | - | 0.01 ±  0  *1* | - | - | - | - |
| Sabinene**^a^** | 972 | 0.01 ±  0  *1* | 0.06 ±  0  *1* | 0.03 ±  0.01  *2* | 0.02 ±  0.003  *3* | 0.09 ±  0.03  *3* | 0.01 ±  0.01  *2* | 0.06 ±  0.01  *5* | 0.05 ±  0.02  *4* | 0.09 ±  0  *1* | - | 0.03 ±  0.002  *2* | 0.06 ±  0.05  *2* | - | - | 0.05 ±  0  *1* | - |
| β-Pinene**^a^** | 977 | - | 0.004±  0  *1* | - | - | - | - | 0.04 ±  0  *1* | - | - | 0.07 ±  0  *1* | - | - | - | - | - | - |
| 6-Methyl-5-heptene-2-one**^a^** | 985 | 0.17 ±  0.08  *4* | 0.09 ±  0.01  *3* | 0.14 ±  0.04  *5* | 0.09 ±  0.03  *8* | 0.15 ±  0.04  *5* | 0.18 ±  0.12  *4* | 0.23 ±  0.06  *7* | 0.17 ±  0.04  *9* | 0.15 ±  0.05  *5* | - | 0.10 ±  0.02  *7* | 0.17 ±  0.06  *4* | - | - | 0.15 ±  0.03  *3* | - |
| β-Myrcene**^a^** | 989 | 0.13 ±  0.04  *7* | 0.07 ±  0.02  *7* | 0.12 ±  0.02  *11* | 0.12 ±  0.02  *11* | 0.11 ±  0.03  *7* | 0.09 ±  0.03  *6* | 0.16 ±  0.03  *11* | 0.19 ±  0.02  *10* | 0.06 ±  0.02  *5* | 0.13 ±  0  *1* | 0.10 ±  0.01  *8* | 0.05 ±  0.02  *5* | - | - | 0.05 ±  0.03  *3* | - |
| p-Cymene**^a^** | 1025 | - | - | - | - | - | - | - | - | - | - | 0.01 ±  0  *1* | - | - | - | - | - |
| Limonene**^a^** | 1029 | 0.07 ±  0.02  *3* | 0.05 ±  0.01  *6* | 0.09 ±  0.01  *10* | 0.07 ±  0.02  *10* | 0.10 ±  0.03  *5* | 0.06 ±  0.02  *4* | 0.11 ±  0.02  *11* | 0.11 ±  0.02  *10* | 0.11 ±  0.04  *5* | 0.14 ±  0  *1* | 0.13 ±  0.04  *8* | 0.10 ±  0.05  *4* | 0.06 ±  0  *1* | - | 0.14 ±  0.05  *3* | - |
| β-Phellandrene**^a^** | 1031 | - | - | - | - | - | - | - | - | - | - | - | - | - | - | - | - |
| Eucalyptol**^a^** | 1032 | - | - | - | 0.005 ±  0  *1* | 0.01 ±  0  *1* | - | - | 0.03 ±  0  *1* | 0.04 ±  0  *1* | - | 0.02 ±  0  *1* | 0.03 ±  0  *1* | - | - | - | - |
| (*Z*)-β-Ocimene**^a^** | 1036 | - | - | - | - | - | - | - | - | - | - | - | - | - | - | - | - |
| (*E*)-β-Ocimene**^a^** | 1046 | - | 0.02 ±  0  *1* | - | 0.02 ±  0.01  *2* | - | - | 0.03 ±  0  *1* | - | - | - | - | - | - | - | - | - |
| α-Pinene oxide**^b^** | 1074 | - | - | - | - | - | - | - | - | - | - | - | - | - | - | - | - |
| α-Terpinolene**^b^** | 1085 | - | - | - | - | - | - | - | - | - | - | - | - | - | - | - | - |
| Linalool**^a^** | 1100 | 0.21 ±  0.07  *6* | 0.10 ±  0.04  *6* | 0.09 ±  0.02  *10* | 0.14 ±  0.03  *9* | 0.11 ±  0.05  *4* | 0.13 ±  0.07  *3* | 0.11 ±  0.03  *9* | 0.09 ±  0.01  *10* | 0.10 ±  0.06  *3* | 0.02 ±  0  *1* | 0.03 ±  0.01  *6* | 0.09 ±  0.01  *2* | 0.03 ±  0  *1* | - | 0.02 ±  0  *1* | - |
| Neo-allo-ocimene**^a^** | 1128 | - | - | - | - | - | - | - | - | - | - | - | - | - | - | - | - |
| 1,3,8-p-Menthatriene**^c^** | 1130 | - | - | - | - | - | - | - | - | - | - | - | - | - | - | - | - |
| (*E*)-Verbenol**^b^** | 1147 | - | - | - | - | - | - | - | - | - | - | - | - | - | - | - | - |
| α-Terpineol**^b^** | 1196 | - | - | - | - | - | - | - | - | - | - | - | - | - | - | - | - |
| Verbenone**^b^** | 1208 | - | - | - | - | - | - | - | - | - | - | - | - | - | - | - | - |
| (*Z*)-Geranylacetone**^a^** | 1448 | - | 0.02 ±  0.01  *2* | - | 0.03 ±  0.01  *3* | - | - | 0.04 ±  0  *1* | 0.04 ±  0.01  *2* | - | - | - | 0.21 ±  0  *1* | - | - | - | - |
|  |  |  |  |  |  |  |  |  |  |  |  |  |  |  |  |  |  |
| ***Sesquiterpenes*** |  |  |  |  |  |  |  |  |  |  |  |  |  |  |  |  |  |
| α-Cubebene**^b^** | 1347 | - | - | - | - | - | - | - | - | - | - | - | - | - | - | - | - |
| α-Longipinene**^b^** | 1352 | - | - | - | - | - | - | - | - | - | - | - | - | - | - | - | - |
| α-Copaene**^a^** | 1376 | - | - | - | - | - | - | - | - | - | - | - | - | - | - | - | - |
| β-Bourbonene**^b^** | 1384 | - | - | - | - | - | - | - | - | - | - | - | - | - | - | - | - |
| β-Cubebene**^b^** | 1388 | - | - | - | - | - | - | - | - | - | - | - | - | - | - | - | - |
| β-Elemene**^b^** | 1390 | 0.03 ±  0  *1* | 0.03 ±  0.02  *2* | 0.03 ±  0.01  *4* | 0.03 ±  0.01  *6* | - | 0.01 ±  0  *1* | 0.04 ±  0.02  *3* | 0.02 ±  0  *1* | - | - | 0.05 ±  0.003  *3* | 0.02 ±  0.01  *2* | - | - | 0.02 ±  0  *1* | - |
| Longifolene**^b^** | 1402 | - | - | - | - | - | - | - | - | - | - | - | - | - | - | - | - |
| α-Gurjunene**^b^** | 1408 | - | - | - | - | - | - | - | - | - | - | - | - | - | - | - | - |
| α-Barbatene**^b^** | 1417 | - | - | - | 0.005 ±  0  *1* | - | - | - | - | - | - | 0.003±  0  *1* | - | - | - | - | - |
| β-Caryophyllene**^a^** | 1421 | - | - | - | - | - | - | - | - | - | - | - | - | - | - | - | - |
| β-Copaene**^b^** | 1432 | - | - | - | - | - | - | - | - | - | - | - | - | - | - | - | - |
| (*Z*)-Thujopsene**^b^** | 1437 | - | - | - | - | - | - | - | - | - | - | - | - | - | - | - | - |
| (*E*)-β-Farnesene**^a^** | 1452 | - | 0.21 ±  0.17  *3* | 0.24 ±  0.09  *4* | 0.08 ±  0  *1* | 0.03 ±  0  *1* | 0.13 ±  0.09  *2* | 0.11 ±  0.03  *2* | - | - | 0.50 ±  0  *1* | 0.21 ±  0.10  *5* | 0.05 ±  0  *1* | - | - | 0.07 ±  0.004  *2* | - |
| α-Humulene**^a^** | 1457 | - | - | - | - | - | - | - | - | - | - | - | - | - | - | - | - |
| α-Curcumene**^b^** | 1481 | - | - | - | 0.02 ±  0  *1* | - | - | - | - | - | - | 0.01 ±  0.00  *2* | - | - | - | - | - |
| γ-Amorphene**^b^** | 1482 | - | - | - | - | - | - | - | - | - | - | - | - | - | - | - | - |
| Germacrene D**^a^** | 1482 | - | - | - | - | - | - | - | - | - | - | - | - | - | - | - | - |
| β-Chamigrene**^b^** | 1484 | - | - | - | - | - | - | - | - | - | - | - | - | - | - | - | - |
| γ-Humulene**^b^** | 1484 | - | - | - | - | - | - | - | - | - | - | - | - | - | - | - | - |
| (*Z,E*)-α-Farnesene**^a^** | 1490 | - | - | - | - | - | - | - | - | - | - | - | - | - | - | - | - |
| β-Himachalene**^b^** | 1501 | - | - | - | - | - | - | - | - | - | - | - | - | - | - | - | - |
| (*E,E*)-α-Farnesene**^a^** | 1504 | 0.09 ±  0.02  *4* | 0.14 ±  0.06  *6* | 0.11 ±  0.02  *8* | 0.12 ±  0.04  *8* | - | 0.04 ±  0.02  *4* | 0.10 ±  0.03  *7* | 0.04 ±  0.01  *7* | - | - | 0.07 ±  0.02  *7* | - | - | - | 0.03 ±  0  *1* | - |
| γ-Cadinene**^b^** | 1514 | - | - | - | - | - | - | - | - | - | - | - | - | - | - | - | - |
| δ-Cadinene**^b^** | 1518 | - | - | - | - | - | - | - | - | - | - | - | - | - | - | - | - |
| (*E*)-Calamenene**^b^** | 1522 | - | - | - | - | - | - | - | - | - | - | - | - | - | - | - | - |
| (*E*)-γ-Bisabolene**^b^** | 1549 | - | - | - | - | - | - | - | - | - | - | - | - | - | - | - | - |
| Caryophyllene oxide**^b^** | 1583 | - | - | - | - | - | - | - | - | - | - | - | - | - | - | - | - |
| Longiverbenone**^b^** | 1648 | - | - | - | - | - | - | - | - | - | - | - | - | - | - | - | - |
| Hexahydrofarnesyl acetone**^a^** | 1840 | 0.07 ±  0.03  *6* | 0.05 ±  0.01  *7* | 0.06 ±  0.02  *9* | 0.03 ±  0.01  *9* | 0.07 ±  0.02  *6* | 0.09 ±  0.02  *5* | 0.05 ±  0.01  *7* | 0.03 ±  0.01  *7* | 0.04 ±  0.01  *4* | 0.07 ±  0  *1* | 0.07 ±  0.05  *6* | 0.05 ±  0.01  *6* | - | - | 0.10 ±  0.07  *3* | - |
|  |  |  |  |  |  |  |  |  |  |  |  |  |  |  |  |  |  |
| ***Homoterpenes*** |  |  |  |  |  |  |  |  |  |  |  |  |  |  |  |  |  |
| (3*E*)-4,8-Dimethyl-  1,3,7-nonatriene**^a^** | 1113 | 0.04 ±  0.02  *4* | 0.06 ±  0.03  *2* | 0.08 ±  0.02  *7* | 0.05 ±  0.01  *8* | 0.08 ±  0.02  *3* | 0.07 ±  0.02  *1* | 0.15 ±  0.04  *4* | 0.08 ±  0.01  *8* | - | - | 0.08 ±  0.02  *5* | 0.06 ±  0  *1* | - | - | - | - |
| (*E*3,*E*7)-4,8,12-Trimethyltrideca-1,3,7,11-tetraene**^b^** | 1572 | - | 0.03 ±  0.02  *2* | 0.04 ±  0.01  *4* | 0.03 ±  0.01  *4* | - | - | 0.02 ±  0  *1* | - | - | - | 0.03 ±  0.01  *3* | - | - | - | 0.03 ±  0  *1* | - |
|  |  |  |  |  |  |  |  |  |  |  |  |  |  |  |  |  |  |
| ***Trisulfides*** |  |  |  |  |  |  |  |  |  |  |  |  |  |  |  |  |  |
| Dimethyltrisulfide**^a^** | 969 | 0.07 ±  0.05  *3* | 0.06 ±  0.01  *2* | - | 0.02 ±  0  *1* | - | - | 0.03 ±  0  *1* | - | 0.19 ±  0  *1* | - | 0.01 ±  0  *1* | - | - | - | 0.08 ±  0  *1* | - |
|  |  |  |  |  |  |  |  |  |  |  |  |  |  |  |  |  |  |
| ***Unknown compounds*** |  |  |  |  |  |  |  |  |  |  |  |  |  |  |  |  |  |
| Unknown1  91,119,134,92,77,105,93,79,  117,41 | 1004 | - | - | - | - | - | - | - | - | - | - | - | - | - | - | - | - |
| Unknown2  69,84,41,81,68,39,53,79,109,  55 | 1072 | - | - | - | - | - | - | - | - | - | - | - | - | - | - | - | - |
| Unknown3  91,119,134,77,117,82,79,41,  39,105 | 1079 | - | - | - | - | - | - | - | - | - | - | - | - | - | - | - | - |
| Unknown4  80,107,79,91,122,70,77,105,  93,41 | 1101 | - | - | - | - | - | - | - | - | - | - | - | - | - | - | - | - |
| Unknown5  119,91,134,77,79,105,92,117,  93,39 | 1121 | - | - | - | - | - | - | - | - | - | - | - | - | - | - | - | - |
| Unknown6  79,117,43,102,81,90,116,77,  89,91 | 1139 | - | - | - | - | - | - | - | - | - | - | - | - | - | - | - | - |
| Unknown7  94,109,91,79,81,95,59,119,77,67 | 1143 | - | - | - | - | - | - | - | - | - | - | - | - | - | - | - | - |
| Unknown8  144,43,101,44,72,73,55,45,  115,42 | 1145 | - | - | - | - | - | - | - | - | - | - | - | - | - | - | - | - |
| Unknown9  70,55,83,69,41,119,134,53,92,81 | 1160 | - | - | - | - | - | - | - | - | - | - | - | - | - | - | - | - |
| Unknown10  142,129,101,143,75,116,130,  103,117,127 | 1173 | - | - | - | - | - | - | - | - | - | - | - | - | - | - | - | - |
| Unknown11  109,43,81,91,152,79,77,93,67,  119 | 1211 | - | - | - | - | - | - | - | - | - | - | - | - | - | - | - | - |
| Unknown12  150,107,135,91,109,79,39,108,  82,77 | 1339 | - | - | - | - | - | - | - | - | - | - | - | - | - | - | - | - |
| Unknown13  138,83,95,109,193,124,137,57,  41,180 | 1344 | - | - | 0.13 ±  0  *1* | - | - | - | 0.47 ±  0  *1* | - | 0.53 ±  0  *1* | - | - | - | - | - | - | - |
| Unknown14  150,91,107,135,79,77,105,108,  95,109 | 1419 | - | - | - | - | - | - | - | - | - | - | - | - | - | - | - | - |
| Unknown15  161,120,105,119,91,81,121,93,  162,41 | 1419 | - | - | - | - | - | - | - | - | - | - | - | - | - | - | - | - |
| Unknown16  161,105,91,119,93,81,79,77,  133,120 | 1445 | - | - | - | - | - | - | - | - | - | - | - | - | - | - | - | - |
| Unknown17  177,220,135,149,163,67,205,  41,91,136 | 1461 | - | 0.42 ±  0.01  *2* | 0.26 ±  0  *1* | - | - | - | - | - | - | - | - | - | - | - | - | - |
| Unknown18  161,39,119,105,91,93,79,133,  204,162 | 1475 | - | - | - | - | - | - | - | - | - | - | - | - | - | - | - | - |
| Unknown19  157,69,115,41,158,142,129,91,  132,117 | 1528 | 0.06 ±  0  *1* | 0.01 ±  0  *1* | 0.03 ±  0.01  *3* | 0.03 ±  0.01  *4* | - | 0.02 ±  0  *1* | 0.03 ±  0  *1* | 0.03 ±  0.01  *6* | - | - | 0.03 ±  0.01  *3* | 0.02 ±  0  *1* | - | - | - | - |
| Unknown20  55,54,84,100,71,57,129,41,111,  56 | 1546 | - | - | - | 0.08 ±  0  *1* | - | - | - | - | - | - | 0.05 ±  0  1 | - | 0.25 ±  0  1 | - | - | - |
| Unknown21  69,81,41,79,134,67,53,93,95,107 | 1554 | - | - | - | - | - | - | - | - | - | - | - | - | - | - | - | - |
| Unknown22  107,135,93,91,204,105,41,77,  79,43 | 1595 | 0.02 ±  0.003  *2* | 0.02 ±  0.003  *2* | 0.03 ±  0.01  *2* | 0.01 ±  0.01  *2* | - | - | 0.03 ±  0  *1* | - | - | - | 0.01 ±  0.0004  *2* | - | - | - | - | - |
| Unknown23  107,135,93,91,204,105,41,77,  79,43 | 1618 | 0.03 ±  0.005  *2* | 0.03 ±  0.001  *2* | 0.02 ±  0  *1* | 0.01 ±  0.01  *2* | - | - | 0.04 ±  0  *1* | - | - | - | 0.01 ±  0  *1* | - | - | - | - | - |
| Unknown24  118,209,224,194,179,119,117,  91,178,210 | 1827 | 0.05 ±  0.03  *2* | - | - | 0.06 ±  0.01  *2* | 0.06 ±  0  *1* | 0.14 ±  0.002  *2* | 0.13 ±  0.08  *2* | 0.03±  0  *1* | 0.09 ±  0  *1* | - | 0.04 ±  0  *1* | 0.34 ±  0  *1* | - | - | 0.31 ±  0  *1* | - |
| Unknown25  98,85,79,67,55,234,41,112,194,  154 | 2317 | 0.06 ±  0  *1* | - | - | - | 0.03 ±  0  *1* | 0.03 ±  0  *1* | - | 0.12 ±  0  *1* | - | - | - | 0.02 ±  0  *1* | - | - | 0.03 ±  0  *1* | - |
| Unknown26  91,117,207,194,115,92,193,208,  103,77 | 2434 | 0.02 ±  0  *1* | 0.02 ±  0  *1* | 0.18 ±  0  *1* | 0.09 ±  0.06  *4* | - | 0.22 ±  0.12  *3* | - | 0.22 ±  0.05  *2* | - | - | - | 0.10 ±  0.08  *2* | 0.15 ±  0  *1* | - | - | - |
| Unknown27  40,44,41,43,91,42,79,78,54,55 | 2690 | - | - | - | - | - | - | - | - | - | - | - | - | - | - | - | - |

|  |  | ***Sinapis alba*** | | | | | | | | | | | | | | | |
| --- | --- | --- | --- | --- | --- | --- | --- | --- | --- | --- | --- | --- | --- | --- | --- | --- | --- |
|  |  | 0 d | | | | 2 d | | | | 7 d | | | | 14 d | | | |
| Compounds | RI | N- C | N- D | N+ C | N+ D | N- C | N- D | N+ C | N+ D | N- C | N- D | N+ C | N+ D | N- C | N-D | N+ C | N+D |
| ***Aliphatics*** |  |  |  |  |  |  |  |  |  |  |  |  |  |  |  |  |  |
| 3-Octanone**^a^** | 985 |  |  |  |  |  |  |  |  |  |  |  |  |  |  |  |  |
| 2-Undecanone**^b^** | 1293 | 0.02 ±  0.01  *3* | 0.04 ±  0.02  *3* | 0.01 ±  0.002  *9* | 0.01 ±  0.002  *10* | 0.04 ±  0.01  *3* | 0.05±  0  *1* | 0.02 ±  0.004  *10* | 0.02 ±  0.01  *7* | 0.01 ±  0  *1* | 0.30±  0  *1* | 0.02 ±  0.01  *6* | 0.02 ±  0.01  *7* | 0.01±  0  *1* | - | 0.02 ±  0.003  *6* | 0.02±  0  *1* |
| 1-Tetradecene**^b^** | 1392 | - | - | - | - | - | - | - | - | - | - | - | - | - | - | - | - |
| 2-Pentadecanone**^a^** | 1698 | - | - | - | - | - | - | - | - | - | - | - | - | - | - | - | - |
|  |  |  |  |  |  |  |  |  |  |  |  |  |  |  |  |  |  |
| ***Aromatics*** |  |  |  |  |  |  |  |  |  |  |  |  |  |  |  |  |  |
| 2-Furanmethanol**^a^** | 859 | - | - | - | 0.004±  0  *1* | - | - | - | - | - | - | - | 0.01 ±  0  *1* | - | - | - | - |
| 1,2-Cyclopentanedione**^b^** | 923 | 0.01 ±  0  *1* | 0.002±  0  *1* | 0.003±  0.0004  *2* | - | - | - | 0.003 ± 0.001  *2* | - | - | - | 0.01 ±  0.01  *3* | 0.004 ±  0  *1* | - | - | 0.01 ±  0  *1* | - |
| Benzaldehyde**^a^** | 962 | 0.11 ±  0.01  *2* | 0.05 ±  0  *1* | 0.21 ±  0.05  *10* | 0.21 ±  0.04  *10* | - | - | 0.13 ±  0.01  *8* | 0.28 ±  0.05  *7* | 0.10 ±  0  *1* | - | 0.21 ±  0.06  *4* | 0.18 ±  0.06  *5* | - | - | 0.10 ±  0.06  *2* | - |
| Benzyl alcohol**^a^** | 1033 | 0.05±  0.03  *2* | 0.02±  0.001  *2* | 0.07 ±  0.02  *10* | 0.06 ±  0.01  *10* | 0.03 ±  0.02  *2* | - | 0.04 ±  0.01  *8* | 0.06 ±  0.01  *8* | 0.01 ±  0  *1* | 0.27±  0  *1* | 0.03 ±  0.01  *4* | 0.05 ±  0.01  *5* | - | - | 0.01 ±  0.001  *4* | 0.06±  0  *1* |
| Phenylacetaldehyde**^a^** | 1042 | - | - | - | - | - | 0.01±  0  *1* | - | - | 0.09 ±  0  *1* | - | - | - | - | - | - | - |
| Acetophenone**^a^** | 1065 | - | - | 0.01 ±  0  *1* | - | - | - | 0.01 ±  0.004  *2* | - | - | - | - | - | - | - | - | - |
| p-Cymenene**^b^** | 1090 | - | - | 0.01 ±  0  *1* | - | - | - | - | - | - | - | 0.01 ±  0  *1* | - | - | - | - | - |
| Methyl benzoate**^a^** | 1096 | - | - | - | 0.01 ±  0  1 | 0.02 ±  0  *1* | - | 0.01 ±  0.001  *2* | - | - | - | - | - | - | - | - | - |
| 2-Phenylethanol**^a^** | 1118 | - | - | - | - | - | - | - | - | - | - | - | - | - | - | - | - |
| Benzyl cyanide**^a^** | 1136 | 0.01 ±  0.002  *2* | 0.005±  0  *1* | 0.02 ±  0.004  *5* | 0.01 ±  0.002  *6* | 0.03 ±  0  *1* | - | 0.02 ±  0.003  *7* | 0.02 ±  0.01  *7* | - | 0.17±  0  *1* | 0.03 ±  0.005  *3* | 0.03 ±  0.01  *5* | - | - | 0.05 ±  0.03  *3* | 0.05±  0  *1* |
| Benzyl acetate**^a^** | 1163 | - | - | 0.01 ±  0.002  *7* | 0.02 ±  0.005  *7* | 0.01 ±  0  *1* | - | 0.01 ±  0.001  *6* | 0.004 ±  0  *1* | - | - | 0.01 ±  0.001  *2* | - | - | - | - | - |
| Methyl salicylate**^a^** | 1191 | 0.03 ±  0.01  *3* | 0.05 ±  0.01  *3* | 0.02 ±  0.01  *8* | 0.02 ±  0.002  *8* | 0.10 ±  0.06  *3* | - | 0.06 ±  0.02  *7* | 0.05 ±  0.02  *5* | 0.02 ±  0  *1* | - | 0.04 ±  0.03  *4* | 0.05 ±  0.03  *5* | 0.07±  0  *1* | - | 0.03 ±  0.01  *4* | 0.04±  0  *1* |
| Coumaran**^b^** | 1217 | - | 0.01 ±  0  *1* | 0.01 ±  0.003  *3* | 0.01 ±  0.003  *2* | - | - | 0.01 ±  0  *1* | - | 0.01 ±  0  *1* | - | - | 0.02 ±  0.002  *3* | - | - | - | - |
| 2-Aminobenzaldehyde**^a^** | 1220 | - | - | 0.002±  0  *1* | - | - | - | - | - | - | - | - | - | - | - | - | - |
| p-Anisaldehyde**^a^** | 1261 | 0.22 ±  0.09  *3* | 0.12 ±  0.02  *3* | 0.21 ±  0.03  *10* | 0.23 ±  0.03  *10* | 0.09 ±  0.08  *2* | *-* | 0.12 ±  0.03  *9* | 0.14 ±  0.04  *9* | 0.16 ±  0  *1* | - | 0.11 ±  0.02  *7* | 0.11 ±  0.03  *6* | 0.04±  0  *1* | - | 0.10 ±  0.04  *5* | - |
| p-Anisyl alcohol**^b^** | 1287 | 0.01 ±  0.003  *2* | 0.01 ±  0  *1* | 0.01 ±  0.002  *3* | 0.01 ±  0.01  *5* | - | - | - | - | 0.01 ±  0  *1* | - | 0.01 ±  0.002  *2* | 0.01 ±  0  *1* | - | - | - | - |
| Indole**^a^** | 1292 | 0.02 ±  0.01  *3* | 0.01 ±  0.01  *2* | 0.03 ±  0.01  *8* | 0.04 ±  0.01  *10* | - | - | 0.02 ±  0.004  *10* | 0.02 ±  0.01  *5* | 0.01 ±  0  *1* | - | 0.02 ±  0.005  *4* | 0.02 ±  0.01  *5* | - | - | 0.01 ±  0  *1* | - |
| 1-Nitro-2-phenylethane**^b^** | 1301 | - | - | - | - | - | - | - | - | - | - | - | - | - | - | - | - |
| Benzyl mustard oil**^b^** | 1364 | - | 0.07 ±  0  *1* | 0.01 ±  0  *1* | 0.01 ±  0.002  *3* | 0.03 ±  0  *1* | - | 0.01 ±  0.0003  *3* | 0.01 ±  0  *1* | - | - | 0.03 ±  0.01  *2* | 0.05 ±  0.02  *3* | - | - | 0.03 ±  0.01  *5* | 0.05±  0  *1* |
| Methyl vanillin**^b^** | 1481 | - | - | 0.004±  0  *1* | 0.002±  0  *1* | - | - | - | - | - | - | - | - | - | - | - | - |
|  |  |  |  |  |  |  |  |  |  |  |  |  |  |  |  |  |  |
| ***Fatty acid-derived*** |  |  |  |  |  |  |  |  |  |  |  |  |  |  |  |  |  |
| (*Z*)*-*3-Hexenol**^a^** | 861 | - | - | - | - | - | - | - | - | - | - | - | - | - | - | - | - |
| Heptanal**^a^** | 903 | - | - | - | - | - | - | - | - | - | - | - | - | - | - | - | 0.02±  0  *1* |
| Amyl acetate**^b^** | 912 | - | - | - | 0.01 ±  0  *1* | 0.01 ±  0  *1* | - | 0.004 ±  0.002  *4* | - | - | - | 0.01 ±  0.003  *3* | - | - | - | 0.01 ±  0.003  *2* | - |
| (*E*)-2-Heptenal**^b^** | 957 | - | - | - | - | - | - | - | - | - | - | - | - | - | - | - | - |
| (*Z*)-1,5-Octadien-3-ol**^c^**  ^match 92%^ | 973 | - | - | - | - | - | - | - | - | - | - | - | 0.01 ±  0.01  *2* | - | - | 0.01 ± 0.001  *2* | - |
| 1-Octen-3-ol**^a^** | 980 | - | - | - | - | - | - | - | - | - | - | - | - | - | - | - | - |
| Octanal**^a^** | 1003 | - | - | - | - | - | - | - | - | - | - | - | - | 0.02±  0  *1* | - | - | - |
| (3*Z*)-3-Hexenyl acetate**^a^** | 1005 | 0.06 ±  0.03  *3* | - | 0.02 ±  0.01  *3* | 0.03 ±  0.01  *7* | 0.16 ±  0.05  *2* | 0.38±  0  *1* | - | 0.28 ±  0.11  *9* | 0.01 ±  0  *1* | - | 0.03 ±  0.01  *3* | 0.23 ±  0.09  *6* | 0.07±  0  *1* | - | 0.12 ±  0.06  *5* | 0.32±  0.02  *2* |
| Hexyl acetate**^a^** | 1012 | 0.01 ±  0.003  *2* | 0.04 ±  0  *1* | - | 0.01 ±  0.01  *2* | 0.03 ±  0.005  *2* | 0.02±  0  *1* | - | 0.03 ±  0.01  *3* | - | - | 0.03 ±  0  *1* | 0.02 ±  0.01  *2* | - | - | 0.03 ±  0.01  *2* | 0.02±  0  *1* |
| Nonanal**^a^** | 1105 | 0.02 ±  0  *1* | - | 0.02 ±  0  *1* | - | - | - | - | - | - | - | - | 0.05 ±  0  *1* | - | - | *-* | 0.38±  0  *1* |
| (*Z*3)-Hexenyl butanoate**^b^** | 1142 | - | - | - | - | - | - | - | - | - | - | - | 0.004 ±  0  *1* | - | - | - | - |
| (2*E*, *Z*6)-Nonadienal**^b^** | 1154 | - | - | - | - | - | - | - | - | - | - | - | 0.01 ±  0  *1* | - | - | - | - |
| p-Mentha-1,5-dien-8-ol**^b^** | 1173 | - | - | 0.01 ±  0.002  *4* | 0.01 ±  0.001  *5* | - | - | 0.01 ±  0.001  *4* | 0.01 ±  0.001  *2* | 0.01 ±  0  *1* | - | 0.01 ±  0.001  *4* | - | - | - | 0.005 ±  0  *1* | - |
| Octanoic acid**^a^** | 1183 | - | - | - | - | - | - | - | - | - | - | - | 0.02 ±  0  *1* | - | - | - | - |
| Decanal**^a^** | 1207 | - | - | 0.01 ±  0  *1* | - | - | - | - | - | - | - | - | 0.13 ±  0.14  *1* | - | - | - | - |
| (*Z*)-3-Hexenyl-  2-methylbutanoate**^a^** | 1230 | - | - | - | - | - | - | - | - | - | - | - | 0.02 ±  0  *1* | - | - | - | - |
| (*Z*3)-Hexenyl- isovalerate**^a^** | 1236 | - | - | - | - | - | - | - | - | - | - | - | - | - | - | - | - |
| Undecanal**^a^** | 1308 | - | - | - | - | - | - | - | - | - | - | - | - | - | - | - | - |
| Decanoic acid**^a^** | 1371 | - | - | - | - | - | - | - | - | - | - | - | - | - | - | - | - |
| Dodecanal**^a^** | 1410 | - | - | - | - | - | - | - | - | - | - | - | - | - | - | - | - |
| 1-Dodecanol**^b^** | 1475 | - | - | - | - | - | - | - | - | - | - | - | - | - | - | - | - |
| 1-Pentadecene**^b^** | 1492 | - | - | - | - | - | - | - | - | - | - | - | - | - | - | - | - |
| Tridecanal**^a^** | 1511 | - | - | - | - | - | - | - | - | - | - | - | - | - | - | - | - |
| Myristic aldehyde**^a^** | 1613 | - | 0.01 ±  0  *1* | - | - | - | 0.02±  0  *1* | - | - | - | - | - | - | - | - | - | - |
| 1-Tetradecanol**^a^** | 1677 | - | - | - | 0.01 ±  0  *1* | - | - | - | - | - | - | - | - | - | - | - | - |
| Methyl myristate**^b^** | 1724 | 0.04 ±  0  *1* | - | - | - | - | - | - | - | - | - | - | 0.03 ±  0  *1* | - | - | - | - |
| Myristic acid**^a^** | 1757 | - | - | 0.003±  0  *1* | - | - | - | - | 0.01 ±  0  *1* | - | - | *-* | 0.03 ±  0  *1* | - | - | - | - |
| Methyl  pentadecanoate**^b^** | 1824 | - | - | - | - | - | - | - | - | - | - | - | 0.02 ±  0  *1* | - | - | - | - |
| Pentadecanoic acid**^b^** | 1863 | - | - | - | - | - | - | - | 0.004±  0  *1* | - | - | - | - | - | - | - | - |
| Methyl palmitate**^a^** | 1925 | 0.23 ±  0  *1* | - | 0.01 ±  0  *1* | - | - | - | 0.04 ±  0.001  *2* | - | - | - | - | - | - | - | 0.23 ±  0  *1* | - |
| Hexadecanoic acid**^a^** | 1974 | - | 0.12 ±  0  *1* | 0.08 ±  0.03  *4* | 0.11 ±  0  *1* | - | - | 0.07 ±  0.02  *6* | 0.10 ±  0  *1* | - | - | 0.17 ±  0.06  *3* | 0.16 ±  0.001  *2* | - | - | 0.22 ±  0  *1* | - |
| Methyl stearate**^b^** | 2125 | 0.13 ±  0  *1* | - | - | - | - | - | 0.02 ±  0  *1* | - | - | - | - | 0.08 ±  0  *1* | - | - | - | - |
| Linolenic acid**^a^** | 2146 | 0.07 ±  0.02  *3* | 0.16 ±  0.08  *2* | 0.19 ±  0.08  *4* | 0.14 ±  0.05  *5* | 0.21 ±  0  *1* | - | 0.14 ±  0.06  *5* | 0.11 ±  0.03  *3* | 0.21 ±  0  *1* | - | 0.15 ±  0.06  *4* | 0.21 ±  0.06  *5* | - | - | 0.23 ±  0.05  *2* | 0.06±  0  *1* |
| Octadecanoic acid**^a^** | 2168 | 0.03 ±  0.01  *2* | 0.04 ±  0  *1* | 0.02 ±  0.01  *5* | 0.03 ±  0.01  *5* | - | - | 0.02 ±  0.01  *5* | 0.04 ±  0.01  *3* | - | - | 0.04 ±  0.02  *4* | 0.04±  0.005  *4* | - | - | 0.09±  0  *1* | - |
| Oleamide**^c^** | 2179 | - | - | 0.01 ±  0  *1* | 0.004±  0  *1* | - | - | - | - | - | - | - | 0.01 ± 0.001  *2* | - | - | - | - |
|  |  |  |  |  |  |  |  |  |  |  |  |  |  |  |  |  |  |
| ***Monoterpenes*** |  |  |  |  |  |  |  |  |  |  |  |  |  |  |  |  |  |
| α-Thujene**^b^** | 924 | - | - | - | - | - | - | - | - | - | - | - | - | - | - | - | - |
| α-Pinene**^a^** | 932 | - | - | - | - | - | - | - | - | - | - | - | - | - | - | - | - |
| Sabinene**^a^** | 972 | - | - | - | - | 0.02 ±  0  *1* | - | - | - | - | - | - | - | - | - | 0.01 ±  0.004  *2* | 0.02±  0  *1* |
| β-Pinene**^a^** | 977 | - | - | - | - | - | - | - | - | - | - | - | - | - | - | - | - |
| 6-Methyl-5-heptene-2-one**^a^** | 985 | - | 0.01 ±  0  *1* | 0.01 ±  0.001  *6* | 0.01 ±  0.01  *4* | 0.05 ±  0.02  *2* | 0.09±  0  *1* | - | 0.02 ±  0.004  *4* | 0.01 ±  0  *1* | - | 0.03 ±  0  *1* | 0.08 ±  0.04  *5* | - | - | 0.01 ±  0  *1* | 0.05±  0.01  *2* |
| β-Myrcene**^a^** | 989 | 0.02 ±  0.01  *2* | 0.03 ±  0.01  *3* | 0.01 ±  0.01  *2* | 0.02 ±  0.005  *3* | 0.06 ±  0.04  *2* | 0.05±  0  *1* | 0.02 ±  0.005  *5* | 0.02 ±  0.00  *2* | 0.01 ±  0  *1* | 0.11±  0  *1* | 0.02 ±  0.003  *3* | 0.03 ±  0.01  *4* | - | - | 0.02 ±  0.01  *6* | 0.07±  0  *1* |
| p-Cymene**^a^** | 1025 | - | - | - | - | - | - | - | - | - | - | - | - | - | - | - | - |
| Limonene**^a^** | 1029 | 0.02 ±  0  *1* | 0.02 ±  0  *1* | - | - | 0.06 ±  0  *1* | - | 0.02 ±  0.0001  *2* | - | - | - | - | - | - | - | 0.04 ±  0  *1* | - |
| β-Phellandrene**^a^** | 1031 | - | - | - | - | - | - | - | - | - | - | - | - | - | - | - | - |
| Eucalypto**l^a^** | 1032 | - | 0.01 ±  0  *1* | 0.004±  0.0005  *2* | 0.004±  0.0002  *2* | - | - | 0.003 ±  0.002  *2* | 0.003±  0  *1* | - | - | 0.01 ±  0  *1* | - | - | - | 0.01 ±  0.01  *3* | - |
| (*Z*)-β-Ocimene**^a^** | 1036 | 0.01 ±  0.003  *3* | 0.03 ±  0.01  *3* | 0.01 ±  0.001  *6* | 0.02 ±  0.01  *7* | 0.02 ±  0.002  *3* | 0.01±  0  *1* | 0.03 ±  0.004  *8* | 0.02 ±  0.01  *3* | 0.03 ±  0  *1* | - | 0.05 ±  0.01  *5* | 0.01 ±  0.003  *3* | 0.01±  0  *1* | - | 0.03 ±  0.005  *6* | 0.04±  0  *1* |
| (*E*)-β-Ocimene**^a^** | 1046 | 0.11 ±  0.02  *3* | 0.15 ±  0.03  *3* | 0.09 ±  0.02  *10* | 0.12 ±  0.02  *10* | 0.12 ±  0.01  *3* | 0.08±  0  *1* | 0.16 ±  0.03  *10* | 0.09 ±  0.03  *7* | 0.14 ±  0  *1* | - | 0.19 ±  0.03  *7* | 0.04 ±  0.02  *6* | 0.07±  0  *1* | - | 0.17 ±  0.03  *6* | 0.21±  0  *1* |
| α-Pinene oxide**^b^** | 1074 | 0.002±  0  *1* | - | 0.01 ±  0.001  *4* | 0.01 ±  0  *1* | - | - | 0.01 ±  0.001  *3* | 0.002±  0  *1* | 0.01 ±  0  *1* | - | 0.04 ±  0.001  *2* | - | - | - | 0.004 ±  0  *1* | - |
| α-Terpinolene**^b^** | 1085 | - | - | - | - | - | - | - | - | - | - | - | - | - | - | - | - |
| Linalool**^a^** | 1100 | - | - | - | - | - | - | - | - | - | - | - | - | - | - | - | - |
| Neo-allo-ocimene**^a^** | 1128 | 0.01 ±  0.001  *2* | 0.01 ±  0.001  *2* | 0.01 ±  0.002  *7* | - | - | - | 0.01 ±  0.004  *6* | 0.01 ±  0  *1* | 0.01 ±  0  *1* | - | 0.01 ±  0.002  *5* | - | - | - | 0.01 ±  0.002  *2* | - |
| 1,3,8-p-Menthatriene**^c^** | 1130 | 0.02 ±  0.005  *3* | 0.03 ±  0.01  *3* | 0.03 ±  0.01  *10* | 0.04 ±  0.01  *10* | 0.01 ±  0.004  *2* | - | 0.04 ±  0.01  *10* | 0.03 ±  0.01  *5* | 0.04 ±  0  *1* | - | 0.05 ±  0.01  *6* | 0.01 ±  0.002  *3* | 0.01±  0  *1* | - | 0.04 ±  0.01  *5* | 0.02±  0  *1* |
| (*E*)-Verbenol**^b^** | 1147 | - | - | - | - | - | - | - | - | - | - | - | - | - | - | - | - |
| α-Terpineol**^b^** | 1196 | 0.003±  0  *1* | - | - | - | 0.03 ±  0  *1* | - | 0.004 ±  0  *1* | - | - | - | - | - | - | - | 0.003 ±  0.001  *2* | - |
| Verbenone**^b^** | 1208 | - | - | - | - | - | - | - | - | - | - | - | - | - | - | - | - |
| (*Z*)-Geranylacetone**^a^** | 1448 | 0.01 ±  0  *1* | - | - | 0.01 ±  0  *1* | 0.01 ±  0  *1* | - | - | 0.01 ±  0  *1* | 0.01 ±  0  *1* | - | 0.01 ±  0  *1* | 0.03 ±  0.01  *4* | - | - | 0.01 ±  0  *1* | - |
|  |  |  |  |  |  |  |  |  |  |  |  |  |  |  |  |  |  |
| ***Sesquiterpenes*** |  |  |  |  |  |  |  |  |  |  |  |  |  |  |  |  |  |
| α-Cubebene**^b^** | 1347 | - | 0.01 ±  0.002  *2* | 0.01 ±  0  *1* | 0.01 ±  0  *1* | - | - | 0.003 ±  0.0004  *4* | - | - | - | 0.002±  0  *1* | - | - | - | 0.01 ±  0  *1* | - |
| α-Longipinene**^b^** | 1352 | - | - | - | - | - | - | - | - | - | - | - | 0.004 ±  0  *1* | - | - | - | - |
| α-Copaene**^a^** | 1376 | - | 0.01 ±  0  *1* | 0.002±  0  *1* | 0.002±  0  *1* | - | - | 0.003 ±  0.0004  *2* | - | - | - | 0.002±  0  *1* | - | - | - | 0.004 ±  0  *1* | - |
| β-Bourbonene**^b^** | 1384 | 0.001±  0  *1* | 0.003±  0  *1* | 0.003±  0  *1* | 0.002±  0  *1* | - | - | 0.004 ±  0.0004  *4* | - | - | - | 0.004±  0.00  *2* | - | - | - | 0.004 ±  0  *1* | - |
| β-Cubebene**^b^** | 1388 | 0.003±  0.001  *2* | 0.02 ±  0.01  *2* | 0.01 ±  0.002  *5* | 0.005±  0.001  *3* | 0.01 ±  0  *1* | 0.02 ±  0  *1* | 0.01 ±  0.002  *6* | 0.01 ±  0  *1* | - | - | 0.01 ±  0.004  *2* | - | - | - | 0.01 ±  0.001  *2* | - |
| β-Elemene**^b^** | 1390 | - | - | 0.001±  0  *1* | - | - | - | - | - | - | - | - | - | - | - | - | - |
| Longifolene**^b^** | 1402 | - | - | - | - | - | - | - | - | - | - | - | - | - | - | - | - |
| α-Gurjunene**^b^** | 1408 | 0.01 ±  0  *1* | 0.01 ±  0.01  *2* | 0.005±  0  *1* | 0.003±  0.001  *2* | - | - | 0.01 ±  0.001  *3* | 0.01 ±  0  *1* | - | - | 0.01 ±  0  *1* | - | - | - | 0.004 ±  0.001  *2* | - |
| α-Barbatene**^b^** | 1417 | - | - | - | - | - | - | - | - | - | - | - | - | - | - | - | - |
| β-Caryophyllene**^a^** | 1421 | - | 0.01 ±  0.01  *2* | 0.08 ±  0.07  *3* | 0.01 ±  0.003  *3* | 0.01 ±  0  *1* | 0.03±  0  *1* | 0.06 ±  0.05  *6* | 0.01 ±  0  *1* | - | - | 0.01 ±  0.003  *3* | - | - | - | 0.01 ±  0.003  *4* | - |
| β-Copaene**^b^** | 1432 | 0.002±  0  *1* | 0.005±  0.003  *2* | 0.01 ±  0.002  *4* | 0.003±  0.001  *4* | - | - | 0.01 ±  0.001  *5* | - | - | - | 0.01 ±  0.0004  *2* | - | - | - | 0.01 ±  0.001  *3* | - |
| (*Z*)-Thujopsene**^b^** | 1437 | - | - | - | - | - | - | - | - | - | - | - | - | - | - | - | - |
| (*E*)-β-Farnesene**^a^** | 1452 | 0.04 ±  0.02  *2* | 0.02 ±  0.01  *2* | 0.05 ±  0  *1* | 0.01 ±  0.005  *2* | 0.03 ±  0.01  *2* | 0.13±  0  *1* | 0.01 ±  0.005  *4* | 0.04 ±  0  *1* | 0.02 ±  0  *1* | - | 0.02 ±  0.01  *2* | 0.09 ±  0.03  *3* | 0.16±  0  *1* | - | 0.08 ±  0.03  *5* | 0.03±  0  *1* |
| α-Humulene**^a^** | 1457 | - | - | 0.02 ±  0  *1* | - | - | - | - | - | - | - | - | - | - | - | - | - |
| α-Curcumene**^b^** | 1481 | - | - | - | - | - | - | - | - | - | - | - | - | - | - | - | - |
| γ-Amorphene**^b^** | 1482 | - | - | - | - | - | - | - | - | - | - | 0.001±  0  *1* | - | - | - | 0.003 ±  0  *1* | - |
| Germacrene D**^a^** | 1482 | 0.01 ±  0  *1* | 0.02 ±  0  *1* | 0.01 ±  0.01  *5* | 0.01 ±  0.001  *2* | 0.01 ±  0  *1* | - | 0.02 ±  0.004  *6* | 0.003±  0  *1* | - | - | 0.01 ±  0.003  *2* | - | - | - | 0.02 ±  0.005  *4* | - |
| β-Chamigrene**^b^** | 1484 | - | - | - | - | - | - | - | - | - | - | - | - | - | - | - | - |
| γ-Humulene**^b^** | 1484 | - | - | - | - | - | - | - | - | - | - | - | - | - | - | - | - |
| (*Z,E*)-α-Farnesene**^a^** | 1490 | - | - | 0.003±  0.001  *4* | - | 0.01 ±  0  *1* | - | 0.01 ±  0.004  *3* | - | - | - | 0.01 ±  0.003  *2* | - | - | - | 0.01 ±  0  *1* | - |
| β-Himachalene**^b^** | 1501 | - | - | - | - | - | - | - | - | - | - | - | - | - | - | - | - |
| (*E,E*)-α-Farnesene**^a^** | 1504 | 0.04 ±  0.01  *2* | 0.01 ±  0  *1* | 0.02 ±  0.01  *7* | 0.03 ±  0.01  *5* | 0.04 ±  0.02  *2* | - | 0.04 ±  0.01  *9* | 0.02 ±  0.005  *5* | 0.01 ±  0  *1* | - | 0.03 ±  0.01  *4* | 0.02 ±  0.01  *2* | 0.01±  0  *1* | - | 0.04 ±  0.01  *3* | 0.02±  0  *1* |
| γ-Cadinene**^b^** | 1514 | - | 0.01 ±  0  *1* | 0.004±  0  *1* | - | - | - | 0.01 ±  0.0004  *2* | - | - | - | - | - | - | - | 0.01 ±  0.002  *2* | - |
| δ-Cadinene**^b^** | 1518 | 0.002±  0  *1* | 0.01 ±  0.005  *2* | 0.01 ±  0.002  *3* | 0.003±  0.001  *4* | - | 0.02±  0  *1* | 0.01 ±  0.001  *5* | 0.01 ±  0  *1* | - | - | 0.005±  0.0002  *2* | - | - | - | 0.01 ±  0  *1* | - |
| (*E*)-Calamenene**^b^** | 1522 | - | - | 0.004±  0  *1* | - | - | - | 0.004 ±  0  *1* | - | - | - | - | - | - | - | - | - |
| (*E*)-γ-Bisabolene**^b^** | 1549 | - | - | - | - | 0.003±  0  *1* | - | 0.003 ±  0  *1* | - | - | - | 0.001±  0  *1* | - | - | - | - | - |
| Caryophyllene oxide**^b^** | 1583 | - | - | 0.01 ±  0  *1* | - | - | - | 0.02 ±  0  *1* | - | - | - | - | - | - | - | - | - |
| Longiverbenone**^b^** | 1648 | - | - | - | - | - | - | - | - | - | - | - | - | - | - | - | - |
| Hexahydrofarnesyl acetone**^a^** | 1840 | 0.01 ±  0.001  *2* | 0.03 ±  0.02  *3* | 0.005±  0.001  *5* | 0.01 ±  0.001  *3* | 0.02 ±  0.01  *3* | - | 0.01 ±  0.004  *7* | 0.01 ±  0  *1* | 0.01 ±  0  *1* | 0.14±  0  *1* | 0.01 ±  0.0003  *2* | 0.02 ±  0.01  *5* | 0.04±  0  *1* | - | 0.02 ±  0.01  *3* | - |
|  |  |  |  |  |  |  |  |  |  |  |  |  |  |  |  |  |  |
| ***Homoterpenes*** |  |  |  |  |  |  |  |  |  |  |  |  |  |  |  |  |  |
| (3*E*)-4,8-Dimethyl-  1,3,7-nonatriene**^a^** | 1113 | 0.02±  0.004  *2* | 0.05 ±  0.02  *3* | 0.01 ±  0.002  *7* | 0.01 ±  0.002  *5* | 0.014±  0.01  *2* | 0.06±  0  *1* | 0.02 ±  0.005  *9* | 0.01 ±  0.002  *5* | 0.01 ±  0  *1* | - | 0.03 ±  0.01  6 | 0.05 ±  0.04  *2* | - | - | 0.02 ±  0.01  *5* | 0.04±  0  *1* |
| (*E*3,*E*7)-4,8,12-Trimethyltrideca-1,3,7,11-tetraene**^b^** | 1572 | 0.02 ±  0.01  *3* | 0.05 ±  0.02  *3* | 0.01 ±  0.005  *6* | 0.01 ±  0.005  *5* | 0.10 ±  0.04  *2* | 0.03±  0  *1* | 0.05 ±  0.02  *7* | 0.005±  0.0004  *3* | 0.03 ±  0  *1* | - | 0.04 ±  0.02  *5* | 0.01 ±  0.0005  *1* | 0.03±  0  *1* | - | 0.03 ±  0.004  *6* | 0.02±  0  *1* |
|  |  |  |  |  |  |  |  |  |  |  |  |  |  |  |  |  |  |
| ***Trisulfides*** |  |  |  |  |  |  |  |  |  |  |  |  |  |  |  |  |  |
| Dimethyltrisulfide**^a^** | 969 | - | - | - | - | - | - | - | - | - | - | - | - | - | - | - | - |
|  |  |  |  |  |  |  |  |  |  |  |  |  |  |  |  |  |  |
| ***Unknown compounds*** |  |  |  |  |  |  |  |  |  |  |  |  |  |  |  |  |  |
| Unknown1  91,119,134,92,77,105,93,79,  117,41 | 1004 | - | - | - | - | - | - | - | - | - | - | - | - | - | - | - | - |
| Unknown2  69,84,41,81,68,39,53,79,109,  55 | 1072 | 0.01 ±  0  *1* | 0.01 ±  0  *1* | 0.01 ±  0.002  *4* | 0.01 ±  0  *4* | - | - | 0.01 ±  0.003  *3* | 0.01 ±  0  *1* | 0.01 ±  0  *1* | - | 0.01 ±  0.002  *3* | - | - | - | 0.02 ±  0  *1* | - |
| Unknown3  91,119,134,77,117,82,79,41,  39,105 | 1079 | - | 0.01 ±  0  *1* | 0.01 ±  0.001  *6* | 0.01 ±  0.001  *7* | - | - | 0.01 ±  0.002  *5* | 0.01 ±  0.001  *3* | 0.01 ±  0  *1* | - | 0.01 ±  0.001  *3* | - | - | - | 0.01 ±  0  *1* | - |
| Unknown4  80,107,79,91,122,70,77,105,  93,41 | 1101 | - | - | 0.01 ±  0  *1* | - | - | - | 0.01 ±  0.001  *2* | - | - | - | 0.01 ±  0  *1* | - | - | - | - | - |
| Unknown5  119,91,134,77,79,105,92,117,  93,39 | 1121 | 0.004±  0.0002  *2* | 0.01 ±  0.01  *2* | 0.01 ±  0.002  *9* | 0.01 ±  0.003  *6* | - | - | 0.01 ±  0.003  *8* | 0.01 ±  0.001  *5* | 0.01 ±  0  *1* | - | 0.01 ±  0.002  *6* | 0.003 ±  0.001  *2* | - | - | 0.01 ±  0.003  *5* | 0.01±  0  *1* |
| Unknown6  79,117,43,102,81,90,116,77,  89,91 | 1139 | - | - | - | - | - | - | - | - | - | - | - | - | - | - | - | - |
| Unknown7  94,109,91,79,81,95,59,119,77,67 | 1143 | - | - | - | - | - | - | - | - | - | - | - | - | - | - | - | - |
| Unknown8  144,43,101,44,72,73,55,45,  115,42 | 1145 | 0.005±  0  *1* | 0.01 ±  0  *1* | 0.01 ±  0  *1* | 0.01 ±  0  *1* | - | - | 0.01 ±  0  *1* | - | 0.004±  0  *1* | - | - | 0.01 ±  0.002  *3* | - | - | - | - |
| Unknown9  70,55,83,69,41,119,134,53,92,81 | 1160 | 0.002±  0  *1* | - | 0.002±  0.0004  *5* | 0.003±  0.0003  *2* | - | - | 0.003 ±  0.001  *3* | 0.003±  0  *1* | 0.002±  0  *1* | - | 0.002±  0.0002  *1* | - | - | - | 0.003 ±  0  *1* | - |
| Unknown10  142,129,101,143,75,116,130,  103,117,127 | 1173 | - | 0.01 ±  0  *1* | 0.004±  0  *1* | 0.001±  0  *1* | 0.03 ±  0  *1* | - | - | - | 0.003±  0  *1* | - | - | 0.01 ±  0.005  *2* | - | - | - | - |
| Unknown11  109,43,81,91,152,79,77,93,67,  119 | 1211 | 0.01 ±  0.004  *2* | 0.02 ±  0  *1* | 0.02 ±  0.01  *6* | 0.02 ±  0.004  *6* | - | - | 0.03 ±  0.01  *6* | 0.01 ±  0.005  *3* | 0.02 ±  0  *1* | - | 0.02 ±  0.01  *5* | - | - | - | 0.01 ±  0.004  *3* | - |
| Unknown12  150,107,135,91,109,79,39,108,  82,77 | 1339 | - | - | 0.001±  0  *1* | - | - | - | 0.002 ±  0  *1* |  | - | - | - | - | - | - | - | - |
| Unknown13  138,83,95,109,193,124,137,57,  41,180 | 1344 | - | - | - | - | - | - | - | - | - | - | - | - | - | - | - | - |
| Unknown14  150,91,107,135,79,77,105,108,  95,109 | 1419 | 0.004±  0  *1* | - | 0.01 ±  0.0004  *2* | 0.01 ±  0  *1* | - | - | 0.01 ±  0.002  *3* | 0.005±  0  *1* | 0.003±  0  *1* | - | 0.01 ±  0  *1* | - | - | - | - | - |
| Unknown15  161,120,105,119,91,81,121,93,  162,41 | 1419 | - | 0.01 ±  0.005  *2* | 0.01 ±  0.003  *2* | 0.01 ±  0.001  *3* | 0.003±  0  *1* | 0.01±  0  *1* | 0.01 ±  0.002  5 | - | - | - | 0.01 ±  0.001  *2* | - | - | - | 0.01 ±  0.002  *2* | - |
| Unknown16  161,105,91,119,93,81,79,77,  133,120 | 1445 |  |  | 0.01 ±  0.00  *2* | 0.003±  0  *1* | - | - | - | - | - | - | - | - | - | - | 0.01 ±  0  *1* | - |
| Unknown17  177,220,135,149,163,67,205,  41,91,136 | 1461 | - | - | 0.09 ±  0  *1* | - | - | - | - | - | - | - | - | 0.30 ±  0  *1* | 0.34±  0  *1* | - | - | - |
| Unknown18  161,39,119,105,91,93,79,133,  204,162 | 1475 | - | - | - | - | - | - | 0.01 ±  0.0004  *2* | - | - | - | 0.003±  0  *1* | - | - | - | - | - |
| Unknown19  157,69,115,41,158,142,129,91,  132,117 | 1528 | 0.001±  0  *1* | 0.003±  0  *1* | 0.01 ±  0  *1* | 0.004±  0  *1* | - | - | 0.004 ±  0.0004  *5* | - | - | - | 0.003±  0.002  *2* | - | - | - | 0.01 ±  0  *1* | - |
| Unknown20  55,54,84,100,71,57,129,41,111,  56 | 1546 | - | - | - | - | - | - | - | - | - | - | - | - | - | - | - | - |
| Unknown21  69,81,41,79,134,67,53,93,95,107 | 1554 | 0.002±  0  *1* | 0.02 ±  0.01  *3* | - | - | 0.04 ±  0.01  *2* | - | 0.03 ±  0.01  *3* | - | - | - | 0.01 ±  0  *1* | - | - | - | 0.01 ±  0  *1* | - |
| Unknown22  107,135,93,91,204,105,41,77,  79,43 | 1595 | 0.01 ±  0.003  *3* | 0.01 ±  0.002  *2* | 0.01 ±  0.002  *3* | 0.01 ±  0.002  *5* | 0.02 ±  0  *1* | 0.02±  0  *1* | 0.02 ±  0.01  *7* | 0.01 ±  0.002  *4* | 0.004±  0  *1* | - | 0.01 ±  0.01  *3* | 0.02 ±  0.01  *2* | - | - | 0.01±  0.002  *5* | - |
| Unknown23  107,135,93,91,204,105,41,77,  79,43 | 1618 | 0.005±  0.001  *3* | 0.004±  0.001  *2* | 0.01 ±  0.001  *4* | 0.01 ±  0.001  *4* | 0.02 ±  0.01  *2* |  | 0.02 ±  0.01  *7* | 0.01 ±  0.002  *4* | 0.003±  0  *1* |  | 0.01 ±  0.004  *3* | 0.02 ±  0.01  *2* |  |  | 0.01 ±  0.002  *5* |  |
| Unknown24  118,209,224,194,179,119,117,  91,178,210 | 1827 | 0.01 ±  0  *1* | 0.07 ±  0  *1* | 0.01 ±  0.001  *5* | - | 0.03 ±  0  *1* | - | 0.01 ±  0.002  *4* | 0.02±  0.01  *2* | 0.003±  0  *1* | - | 0.01 ±  0  *1* | - | 0.04±  0  *1* | - | 0.01 ±  0.001  *3* | - |
| Unknown25  98,85,79,67,55,234,41,112,194,  154 | 2317 | 0.01 ±  0.001  *2* | 0.03 ±  0.01  *2* | 0.02 ±  0.01  *4* | 0.02 ±  0.002  *4* | 0.09 ±  0  *1* | - | 0.02 ±  0.005  *4* | 0.02 ±  0.004  *3* | 0.02 ±  0  *1* | - | 0.02 ±  0.002  *2* | 0.04 ±  0.01  *4* | - | - | 0.03 ±  0.004  *2* | 0.02±  0  *1* |
| Unknown26  91,117,207,194,115,92,193,208,  103,77 | 2434 | - | 0.01 ±  0  *1* | - | - | 0.07 ±  0  *1* | - | - | 0.01 ±  0  *1* | - | - | 0.01 ±  0.002  *2* | 0.01 ±  0  *1* | - | - | - | 0.0±  0  *1* |
| Unknown27  40,44,41,43,91,42,79,78,54,55 | 2690 | - | 0.02 ±  0  *1* | - | 0.01 ±  0  *1* | - | - | 0.02 ±  0  *1* | - | - | - | - | 0.02 ±  0  *1* | - | - | - | - |

|  |  | ***Sinapis arvensis*** | | | | | | | | | | | | | | | |
| --- | --- | --- | --- | --- | --- | --- | --- | --- | --- | --- | --- | --- | --- | --- | --- | --- | --- |
|  |  | 0 d | | | | 2 d | | | | 7 d | | | | 14 d | | | |
| Compounds | RI | N- C | N- D | N+ C | N+ D | N- C | N- D | N+ C | N+ D | N- C | N- D | N+ C | N+ D | N- C | N- D | N+ C | N+D |
|  |  |  |  |  |  |  |  |  |  |  |  |  |  |  |  |  |  |
| ***Aliphatics*** |  |  |  |  |  |  |  |  |  |  |  |  |  |  |  |  |  |
| 3-Octanone**^a^** | 985 | 0.03 ±  0  *1* | - | 0.02 ±  0  *1* | 0.03 ±  0  *1* | - | - | - | - | - | - | - | - | - | - | 0.01 ±  0  *1* | - |
| 2-Undecanone**^b^** | 1293 | - | - | - | - | - | - | 0.02 ±  0  *1* | - | - | - | - | - | - | - | - | 0.01 ±  0  *1* |
| 1-Tetradecene**^b^** | 1392 | - | - | - | - | - | - | - | - | - | - | - | - | - | - | - | - |
| 2-Pentadecanone**^a^** | 1698 | - | - | - | - | - | - | - | - | - | - | - | - | - | - | - | - |
|  |  |  |  |  |  |  |  |  |  |  |  |  |  |  |  |  |  |
| ***Aromatics*** |  |  |  |  |  |  |  |  |  |  |  |  |  |  |  |  |  |
| 2-Furanmethanol**^a^** | 859 | - | - | - | - | - | - | - | - | - | - | - | - | - | - | - | - |
| 1,2-Cyclopentanedione**^b^** | 923 | - | - | - | - | - | 0.003± 0  *1* | 0.003± 0  *1* | 0.003± 0  *1* | - | 0.003±  0  *1* | - | - | - | 0.01 ±  0.003  *2* | - | - |
| Benzaldehyde**^a^** | 962 | 0.60 ±  0.01  *2* | 0.03 ±  0.02  *2* | 0.08 ±  0.03  *4* | 0.07 ±  0.04  *2* | 0.06 ±  0.01  *3* | 0.06 ±  0.004  *2* | 0.06 ±  0  *1* | 0.09 ±  0.03  *3* | 0.11 ±  0  *1* | - | 0.09 ±  0.05  *2* | 0.16 ±  0.06  *3* | 0.02 ±  0  *1* | 0.10 ±  0  *1* | 0.04 ±  0.02  *3* | 0.11±  0.04  *2* |
| Benzyl alcohol**^a^** | 1033 | 0.01 ±  0.001  *2* | - | 0.01 ±  0.001  *4* | 0.03 ±  0.01  *4* | 0.03 ±  0.004  *2* | 0.02 ±  0.01  *3* | 0.03 ±  0.02  *3* | - | 0.01 ±  0  *1* | 0.01 ±  0.003  *2* | 0.02 ±  0.01  *2* | 0.02 ±  0.01  *2* | 0.02 ±  0.002  *2* | 0.04 ±  0.03  *2* | 0.01 ±  0  *1* | 0.04 ±  0.01  *2* |
| Phenylacetaldehyde**^a^** | 1042 | - | - | - | - | - | - | - | - | - | - | - | - | - | - | - | - |
| Acetophenone**^a^** | 1065 | - | - | - | - | 0.05 ±  0  *1* | - | - | 0.01 ±  0  *1* | - | 0.01 ±  0  *1* | *-* | - | 0.005 ±  0  *1* | - | - | - |
| p-Cymenene**^b^** | 1090 | - | 0.01 ±  0  *1* | - | - | - | - | 0.02 ±  0  *1* | - | 0.01 ±  0  *1* | 0.01 ±  0  *1* | 0.02 ±  0  *1* | 0.01 ±  0  *1* | - | - | - | - |
| Methyl benzoate**^a^** | 1096 | - | - | - | - | - | - | 0.02 ±  0  *1* | - | - | - | - | - | - | - | - | - |
| 2-Phenylethanol**^a^** | 1118 | - | - | 0.003±  0.001  *2* | - | - | 0.003±  0  *1* | 0.005 ±  0.002  *2* | - | *-* | - | 0.01 ±  0  *1* | - | 0.01 ±  0  *1* | 0.004±  0  *1* | - | 0.02 ±  0.003  *2* |
| Benzyl cyanide**^a^** | 1136 | 0.02 ±  0  *1* | 0.03 ±  0  *1* | 0.02 ±  0.002  *2* | 0.02 ±  0  *1* | 0.04 ±  0  *1* | 0.03 ±  0  *1* | 0.01 ±  0  *1* | - | - | 0.02 ±  0  *1* | 0.03 ±  0  *1* | 0.04 ±  0.003  *2* | 0.01 ±  0  *1* | - | 0.03 ±  0  *1* | 0.04 ±  0  *1* |
| Benzyl acetate**^a^** | 1163 | - | - | - | - | - | - | - | - | - | - | - | - | - | - | - | - |
| Methyl salicylate**^a^** | 1191 | 0.02 ±  0  *1* | 0.01 ±  0  *1* | 0.01 ±  0  *1* | - | 0.03 ±  0  *1* | - | 0.01 ±  0  *1* | - | - | - | - | - | 0.01 ±  0.005  *2* | - | - | - |
| Coumaran**^b^** | 1217 | - | - | - | - | - | - | 0.003 ±  0  *1* | 0.01 ±  0  *1* | - | 0.01 ±  0  *1* | - | - | - | - | - | - |
| 2-Aminobenzaldehyde**^a^** | 1220 | - | 0.01 ±  0  *1* | 0.004±  0  *1* | 0.03 ±  0  *1* | 0.07 ±  0.05  *2* | 0.04 ±  0  *1* | 0.01 ±  0  *1* | 0.02 ±  0.01  *3* | 0.02 ±  0.02  *2* | - | 0.04 ±  0.03  *2* | - | 0.05 ±  0  *1* | - | 0.08 ±  0  *1* | - |
| p-Anisaldehyde**^a^** | 1261 | - | - | - | - | - | - | - | - | - | - | - | - | - | - | - | - |
| p-Anisyl alcohol**^b^** | 1287 | - | - | - | - | - | - | - | - | - | - | - | - | - | - | - | - |
| Indole**^a^** | 1292 | 0.02 ±  0.003  *3* | 0.03 ±  0.01  *3* | 0.01 ±  0.002  *5* | 0.02 ±  0.01  *1* | 0.06 ±  0.03  *3* | 0.03 ±  0.01  *3* | 0.03 ±  0.02  *3* | 0.02 ±  0.01  *4* | 0.04 ±  0.02  *3* | 0.01 ±  0.01  *2* | 0.03 ±  0.01  *4* | 0.02 ±  0.004  *2* | 0.03 ±  0.03  *2* | 0.01 ±  0  *1* | 0.02 ±  0.01  *2* | - |
| 1-Nitro-2-phenylethane**^b^** | 1301 | 0.01 ±  0  *1* | - | 0.01 ±  0.002  *2* | - | 0.02 ±  0  *1* | 0.01 ±  0  *1* | - | - | 0.01 ±  0  *1* | 0.01 ±  0  *1* | 0.02 ±  0  *1* | - | 0.04 ±  0  *1* | - | 0.01 ±  0.  *1* | 0.02 ±  0  *1* |
| Benzyl mustard oil**^b^** | 1364 | - | - | - | - | - | - | - | - | - | - | - | - | - | - | - | - |
| Methyl vanillin**^b^** | 1481 | - | - | - | - | - | - | - | - | - | - | - | - | - | - | - | - |
|  |  |  |  |  |  |  |  |  |  |  |  |  |  |  |  |  |  |
| ***Fatty acid-derived*** |  |  |  |  |  |  |  |  |  |  |  |  |  |  |  |  |  |
| (*Z*)*-*3-Hexenol**^a^** | 861 | - | - | - | 0.01 ±  0  *1* | - | - | - | 0.03 ±  0  *1* | - | - | - | - | - | - | - | - |
| Heptanal**^a^** | 903 | - | - | - | - | - | - | - | - | - | - | - | - | - | - | - | - |
| Amyl acetate**^b^** | 912 | - | - | - | - | - | - | - | - | - | - | - | - | - | - | - | - |
| (*E*)-2-Heptenal**^b^** | 957 | - | - | - | - | - | - | - | - | *-* | - | - | - | - | - | - | 0.01± 0.004  *2* |
| (*Z*)-1,5-Octadien-3-ol**^c^**  ^match 92%^ | 973 | 0.23 ± 0  *1* | 0.02 ± 0  *1* | 0.06 ± 0.03  *3* | 0.05 ± 0.03  *2* | 0.14 ± 0  *1* | 0.02 ± 0  *1* | 0.15 ± 0.08  *3* | - | - | 0.003 ± 0  *1* | - | 0.14 ±  0  *1* | - | - | 0.01 ± 0.004  *2* | 0.11± 0.11  *2* |
| 1-Octen-3-ol**^a^** | 980 | 0.21 ± 0  *1* | - | 0.04 ± 0.02  *3* | 0.06 ± 0  *1* | 0.12 ± 0  *1* | 0.01 ± 0  *1* | 0.17 ± 0.09  *2* | - | - | - | - | 0.09 ±  0  *1* | - | - | - | 0.15 ± 0  *1* |
| Octanal**^a^** | 1003 | - | - | - | - | - | - | - | - | - | - | 0.01 ±  0  *1* | - | - | - | - | - |
| (3*Z*)-3-Hexenyl acetate**^a^** | 1005 | 0.06 ±  0  *1* | 0.12 ±  0.06  *2* | 0.05 ±  0.02  *3* | 0.11 ±  0.06  *4* | - | 0.20 ±  0.01  *2* | 0.12 ±  0.03  *3* | 0.09 ±  0.05  *5* | 0.05 ±  0  *1* | - | 0.04 ±  0.01  *4* | 0.08 ±  0.01  *3* | 0.08 ± 0.06  *2* | - | 0.06 ±  0.02  *4* | 0.07± 0.02  *2* |
| Hexyl acetate**^a^** | 1012 | 0.01 ±  0  *1* | - | 0.01 ±  0  *1* | - | 0.02 ±  0  *1* | 0.02 ±  0  *1* | 0.01 ±  0.004  *3* | - | - | - | - | - | - | - | - | - |
| Nonanal**^a^** | 1105 | 0.02 ±  0  *1* | - | - | - | - | 0.02 ±  0  *1* | - | - | - | - | 0.02 ±  0.01  *2* | - | 0.02 ±  0.01  *2* | - | 0.01 ±  0  *1* | 0.09 ±  0  *1* |
| (*Z*3)-Hexenyl butanoate**^b^** | 1142 | - | - | - | - | - | 0.02 ±  0  *1* | - | - | - | - | - | - | - | - | - | - |
| (2*E*, *Z*6)-Nonadienal**^b^** | 1154 | - | - | - | - | - | - | - | - | - | - | - | - | - | - | - | 0.01 ±  0  *1* |
| p-Mentha-1,5-dien-8-ol**^b^** | 1173 | 0.01 ±  0.002  *2* | 0.01 ±  0.001  *2* | - | 0.01 ±  0  *1* | 0.01 ±  0.001  *2* | 0.01 ±  0  *1* | 0.01 ±  0  *1* | 0.005±  0  *1* | 0.01 ±  0  *1* | - | 0.01 ±  0.003  *3* | - | 0.02 ±  0  *1* | - | 0.01 ±  0.002  *3* | - |
| Octanoic acid**^a^** | 1183 | - | - | - | - | - | - | - | - | - | - | - | - | - | - | - | 0.003±  0  *1* |
| Decanal**^a^** | 1207 | - | - | - | - | - | - | - | - | - | - | 0.02 ±  0.01  *2* | - | 0.01 ±  0  *1* | - | 0.02 ±  0  *1* | 0.07 ±  0  *1* |
| (*Z*)-3-Hexenyl-  2-methylbutanoate**^a^** | 1230 | - | - | - | - | - | 0.01 ±  0  *1* | - | - | - | - | - | - | - | - | - | - |
| (*Z*3)-Hexenyl- isovalerate**^a^** | 1236 | - | - | 0.004±  0  *1* | - | - | 0.01 ±  0  *1* | - | - | - | - | - | - | - | - | - | - |
| Undecanal**^a^** | 1308 | - | - | - | - | - | - | - | - | - | - | - | - | - | - | - | - |
| Decanoic acid**^a^** | 1371 | - | - | - | - | - | - | - | - | - | - | - | - | - | - | - | 0.17 ±  0  *1* |
| Dodecanal**^a^** | 1410 | - | - | - | - | - | - | - | - | - | - | - | - | - | - | - | - |
| 1-Dodecanol**^b^** | 1475 | - | - | - | - | - | - | - | - | - | - | - | - | - | - | - | - |
| 1-Pentadecene**^b^** | 1492 | - | - | - | - | - | - | - | - | - | - | - | - | - | - | - | - |
| Tridecanal**^a^** | 1511 | - | - | - | - | - | - | - | - | - | - | - | - | - | - | - | - |
| Myristic aldehyde**^a^** | 1613 | - | - | - | - | - | - | - | - | - | - | - | - | - | - | - | - |
| 1-Tetradecanol**^a^** | 1677 | - | - | 0.01 ±  0  *1* | 0.03 ±  0.02  *2* | - | - | - | - | - | - | - | - | - | - | - | - |
| Methyl myristate**^b^** | 1724 | - | - | - | - | - | - | - | - | - | - | - | - | - | - | - | - |
| Myristic acid**^a^** | 1757 | - | - | 0.01 ±  0  *1* | - | - | - | - | - | - | - | - | - | - | - | - | - |
| Methyl  pentadecanoate**^b^** | 1824 | - | - | - | 0.07 ±  0  *1* | - | - | - | - | - | - | - | - | - | - | - | - |
| Pentadecanoic acid**^b^** | 1863 | - | - | - | - | - | - | - | - | - | - | - | - | - | - | - | - |
| Methyl palmitate**^a^** | 1925 | - | - | - | - | - | - | - | - | 0.01 ±  0  *1* | - | - | - | - | 0.01 ±  0  *1* | - | - |
| Hexadecanoic acid**^a^** | 1974 | - | 0.07 ±  0.03  *3* | 0.08 ±  0.02  *5* | 0.16 ±  0.08  *2* | - | 0.12 ±  0.11  *2* | 0.15 ±  0.02  *3* | 0.10 ±  0.06  *3* | 0.07 ±  0.03  *3* | 0.17 ±  0.01  *4* | 0.05 ±  0.01  *5* | 0.10 ±  0.04  *3* | 0.04 ±  0.02  *2* | 0.26 ±  0.004  *2* | 0.08 ±  0.04  *3* | 0.07 ±  0.02  *2* |
| Methyl stearate**^b^** | 2125 | - | - | - | 0.01 ±  0  *1* | - | - | - | - | 0.01 ±  0  *1* | - | - | - | - | 0.02 ±  0  *1* | - | - |
| Linolenic acid**^a^** | 2146 | 0.01 ±  0  *1* | 0.04 ±  0  *1* | 0.08 ±  0.03  *6* | 0.28 ±  0  *1* | 0.04 ±  0  *1* | 0.22 ±  0  *1* | 0.15 ±  0.06  *3* | 0.20 ±  0.16  *2* | 0.08 ±  0.01  *2* | 0.19 ±  0.05  *4* | 0.04 ±  0.02  *3* | 0.02 ±  0  *1* | 0.06 ±  0  *1* | 0.29 ±  0.06  *2* | 0.15 ±  0.09  *2* | 0.02 ±  0  *1* |
| Octadecanoic acid**^a^** | 2168 | - | 0.05 ±  0.02  *2* | 0.03 ±  0.01  *6* | 0.11 ±  0  *1* | - | 0.10 ±  0  *1* | 0.05 ±  0.004  *3* | 0.04 ±  0.02  *3* | 0.03 ±  0.003  *2* | 0.06 ±  0.01  *4* | 0.01 ±  0.002  *4* | 0.02 ±  0  *1* | 0.02 ±  0  *1* | 0.11 ±  0  *1* | 0.02 ±  0.01  *2* | 0.03 ±  0  *1* |
| Oleamide**^c^** | 2179 | - | - | - | - | - | - | - | - | - | 0.01 ± 0  *1* | - | - | - | - | - | - |
|  |  |  |  |  |  |  |  |  |  |  |  |  |  |  |  |  |  |
| ***Monoterpenes*** |  |  |  |  |  |  |  |  |  |  |  |  |  |  |  |  |  |
| α-Thujene**^b^** | 924 | - | - | - | - | - | - | - | - | - | - | - | - | - | - | - | - |
| α-Pinene**^a^** | 932 | 0.03 ±  0.01  *3* | 0.08 ±  0  *1* | 0.01 ±  0.01  *4* | 0.01 ±  0.01  *4* | 0.17 ±  0.14  *3* | 0.06 ±  0.06  *2* | 0.03 ±  0.01  *5* | 0.08 ±  0  *1* | 0.02 ±  0  *1* | 0.03 ±  0.005  *2* | 0.01 ±  0.01  *2* | 0.06 ±  0.04  *3* | - | 0.04 ±  0  *1* | - | 0.24 ±  0.23  *3* |
| Sabinene**^a^** | 972 | 0.01 ±  0  *1* | 0.02 ±  0.01  *2* | 0.02 ±  0.01  *2* | 0.01 ±  0  *1* | 0.02 ±  0.005  *2* | 0.03 ±  0  *1* | 0.03 ±  0.01  *3* | 0.03 ±  0  *1* | 0.01 ±  0  *1* | 0.01 ±  0.001  *2* | 0.01 ±  0  *1* | 0.01 ±  0.002  *3* | - | - | - | - |
| β-Pinene**^a^** | 977 | - | - | - | 0.01 ±  0  *1* | 0.03 ±  0  *1* | 0.02 ±  0  *1* | 0.01 ±  0.001  *2* | 0.02 ±  0  *1* | - | - | - | 0.02 ±  0  *1* | - | - | - | - |
| 6-Methyl-5-heptene-2-one**^a^** | 985 | 0.03 ±  0  *1* | 0.01 ±  0  *1* | - | 0.01 ±  0.001  *2* | - | 0.02 ±  0.01  *2* | 0.03 ±  0.02  *2* | - | - | - | 0.01 ±  0.001  *2* | 0.09 ±  0  *1* | 0.01 ±  0.0002  *2* | - | 0.02 ±  0.01  *3* | - |
| β-Myrcene**^a^** | 989 | 0.06 ±  0.01  *6* | 0.08 ±  0.02  *6* | 0.07 ±  0.01  *9* | 0.06 ±  0.01  *8* | 0.07 ±  0.02  *6* | 0.06 ±  0.02  *5* | 0.07 ±  0.01  *8* | 0.05 ±  0.01  *6* | 0.06 ±  0.01  *2* | 0.06 ±  0.01  *3* | 0.03 ±  0.01  *5* | 0.07 ±  0.01  *3* | 0.03 ±  0.01  *3* | 0.07 ±  0.05  *3* | 0.05 ±  0.01  *3* | 0.06 ±  0.01  *3* |
| p-Cymene**^a^** | 1025 | - | 0.01 ±  0.0005  *2* | - | - | - | 0.005±  0  *1* | 0.004 ±  0  *1* | - | 0.004 ±  0  *1* | - | - | - | - | - | - | - |
| Limonene**^a^** | 1029 | 0.04 ±  0.01  *6* | 0.07 ±  0.02  *6* | 0.06 ±  0.02  *8* | 0.06 ±  0.01  *7* | 0.06 ±  0.02  *6* | 0.06 ±  0.02  *5* | 0.06 ±  0.01  *8* | 0.05 ±  0.01  *6* | 0.05 ±  0.01  *2* | 0.06 ±  0.01  *3* | 0.04 ±  0.01  *4* | 0.08 ±  0.01  *4* | 0.02 ±  0.01  *3* | 0.07 ±  0.04  *3* | 0.03 ±  0.003  *3* | 0.06 ±  0.01  *3* |
| β-Phellandrene**^a^** | 1031 | 0.04 ±  0.01  *6* | 0.06 ±  0.02  *6* | 0.04 ±  0.01  *8* | 0.05 ±  0.01  *7* | 0.05 ±  0.01  *6* | 0.05 ±  0.02  *5* | 0.05 ±  0.01  *8* | 0.04 ±  0.01  *6* | 0.04 ±  0.01  *2* | 0.05 ±  0.01  *3* | 0.03 ±  0.01  *5* | 0.05 ±  0.01  *4* | 0.01 ±  0.005  *3* | 0.05 ±  0.03  *3* | 0.02 ±  0.01  *3* | 0.04 ±  0.01  *3* |
| Eucalypto**l^a^** | 1032 | - | - | - | - | - | - | - | - | - | - | - | - | - | - | - | - |
| (*Z*)-β-Ocimene**^a^** | 1036 | 0.04 ±  0.01  *5* | 0.04 ±  0.004  *3* | 0.04 ±  0.003  *7* | 0.04 ±  0.005  *3* | 0.04 ±  0.01  *4* | 0.03 ±  0.01  *2* | 0.04 ±  0.01  *4* | 0.04 ±  0.01  *3* | 0.04 ±  0.01  *3* | 0.03 ±  0  *1* | 0.03 ±  0.01  *4* | 0.05 ±  0.0003  *2* | 0.04 ±  0.02  *3* | 0.01 ±  0  *1* | 0.04 ±  0.01  *5* | 0.02 ±  0.02  *2* |
| (*E*)-β-Ocimene**^a^** | 1046 | 0.30 ±  0.04  *6* | 0.21 ±  0.06  *5* | 0.23 ±  0.02  *9* | 0.25 ±  0.03  *8* | 0.28 ±  0.05  *6* | 0.23 ±  0.05  *4* | 0.22 ±  0.03  *8* | 0.21 ±  0.03  *6* | 0.31 ±  0.05  *3* | 0.17 ±  0.04  *4* | 0.24 ±  0.03  *5* | 0.26 ±  0.05  *5* | 0.30 ±  0.09  *3* | 0.11 ±  0.03  *3* | 0.27 ±  0.05  *5* | 0.25 ±  0.04  *2* |
| α-Pinene oxide**^b^** | 1074 | 0.01 ±  0.003  *2* | 0.01 ±  0  *1* | 0.01 ±  0.003  *2* | 0.004±  0  *1* | 0.01 ±  0.005  *2* | 0.04 ±  0  *1* | 0.01 ±  0.0004  *3* | 0.01 ±  0  *1* | 0.01 ±  0  *1* | - | 0.02 ±  0  *1* | - | 0.01 ±  0  *1* | - | 0.01 ±  0.002  *3* | - |
| α-Terpinolene**^b^** | 1085 | 0.01 ±  0.00  *2* | 0.01 ±  0  *1* | 0.01 ±  0.001  *6* | 0.01 ±  0.002  *2* | 0.003±  0.001  *2* | 0.01 ±  0  *1* | 0.01 ±  0.002  *3* | 0.01 ±  0.002  *2* | 0.01 ±  0  *1* | - | 0.004±  0  *1* | - | 0.003 ±  0.001  *2* | - | 0.01 ±  0.003  *3* | - |
| Linalool**^a^** | 1100 | - | - | - | 0.01 ±  0  *1* | - | - | - | 0.01 ±  0  *1* | - | - | - | - | - | - | - | - |
| Neo-allo-ocimene**^a^** | 1128 | 0.02 ±  0.004  *5* | 0.01 ±  0.003  *4* | 0.01 ±  0.002  *7* | 0.01 ±  0.002  *6* | 0.02 ±  0.003  *4* | 0.01 ±  0.002  *3* | 0.01 ±  0.003  *5* | 0.01 ±  0.001  *5* | 0.01 ±  0.003  *3* | 0.01 ±  0.002  *4* | 0.01 ±  0.002  *5* | 0.01 ±  0.002  3 | 0.01 ±  0.004  *3* | 0.01 ±  0  *1* | 0.01 ±  0.002  *5* | 0.01 ±  0  *1* |
| 1,3,8-p-Menthatriene**^c^** | 1130 | 0.06 ±  0.01  *6* | 0.04 ±  0.01  *6* | 0.04 ±  0.01  *9* | 0.04 ±  0.01  *7* | 0.05 ±  0.01  *4* | 0.04 ±  0.01  *4* | 0.04 ±  0.01  *7* | 0.03 ±  0.01  *6* | 0.05 ±  0.002  *3* | 0.03 ±  0.01  *4* | 0.06 ±  0.01  *5* | 0.04 ±  0.004  *4* | 0.05 ±  0.02  *3* | 0.01 ±  0.002  *2* | 0.06 ±  0.01  *5* | 0.03 ±  0  *1* |
| (*E*)-Verbenol**^b^** | 1147 | 0.01 ±  0.003  *4* | 0.02 ±  0.004  *5* | 0.01 ±  0.003  *7* | 0.01 ±  0.003  *5* | 0.01 ±  0.002  *3* | 0.01 ±  0.001  *4* | 0.01 ±  0.003  *5* | 0.01 ±  0.003  *4* | 0.01 ±  0.003  *2* | 0.01 ±  0.001  *2* | 0.01 ±  0.003  *5* | 0.005 ±  0.001  *2* | 0.01 ±  0.001  *2* | 0.01 ±  0.003  *2* | 0.01 ±  0.004  *4* | 0.02 ±  0  *1* |
| α-Terpineol**^b^** | 1196 | 0.03 ±  0.01  *6* | 0.03 ±  0.01  *6* | 0.02 ±  0.004  *9* | 0.03 ±  0.01  *8* | 0.03 ±  0.004  *4* | 0.02 ±  0.003  *4* | 0.03 ±  0.01  *6* | 0.02 ±  0.001  *5* | 0.02 ±  0.01  *2* | 0.03 ±  0.02  *2* | 0.03 ±  0.004  *5* | 0.01 ±  0.003  *3* | 0.03 ±  0.003  *3* | 0.02 ±  0.02  *2* | 0.02 ±  0.003  *5* | 0.01 ±  0.003  *2* |
| Verbenone**^b^** | 1208 | 0.12 ±  0.02  *6* | 0.14 ±  0.03  *6* | 0.10 ±  0.01  *9* | 0.12 ±  0.02  *8* | 0.12 ±  0.02  *4* | 0.11 ±  0.04  *5* | 0.10 ±  0.02  *8* | 0.09 ±  0.01  *5* | 0.08 ±  0.03  *3* | 0.06 ±  0.02  *4* | 0.13 ±  0.02  *5* | 0.07 ±  0.003  *5* | 0.11 ±  0.01  *2* | 0.11 ±  0.04  *3* | 0.11 ±  0.02  *5* | 0.07 ±  0.02  *3* |
| (*Z*)-Geranylacetone**^a^** | 1448 | 0.01 ±  0  *1* | - | - | - | - | 0.01 ±  0  *1* | 0.03 ±  0  *1* | - | - | - | 0.01 ±  0  *1* | - | - | - | - | - |
|  |  |  |  |  |  |  |  |  |  |  |  |  |  |  |  |  |  |
| ***Sesquiterpenes*** |  |  |  |  |  |  |  |  |  |  |  |  |  |  |  |  |  |
| α-Cubebene**^b^** | 1347 | - | - | - | - | - | - | - | - | - | - | 0.003±  0  *1* | - | - | - | - | 0.01 ±  0  *1* |
| α-Longipinene**^b^** | 1352 | 0.06 ±  0.01  *3* | 0.09 ±  0.01  *3* | 0.11 ±  0.02  *3* | 0.09 ±  0  *4* | 0.07 ±  0.02  *2* | 0.06 ±  0.004  *2* | 0.12 ±  0.04  *3* | 0.11 ±  0.01  *3* | 0.04 ±  0  *1* | 0.04 ±  0  *2* | 0.04 ±  0  *1* | 0.04 ±  0.09  *2* | 0.08 ±  0  *1* | 0.04 ±  0  *1* | 0.08 ±  0  *1* | 0.08 ±  0.02  *2* |
| α-Copaene**^a^** | 1376 | - | - | - | - | - | - | - | - | - | - | - | - | - | - | - | - |
| β-Bourbonene**^b^** | 1384 | - | - | - | - | - | - | - | - | - | - | - | - | - | - | - | - |
| β-Cubebene**^b^** | 1388 | 0.01 ±  0  *1* | 0.01 ±  0.001  *2* | 0.01 ±  0.0004  *2* | 0.01 ±  0  *1* | 0.003±  0  *1* | 0.005±  0  *1* | 0.01 ±  0  *1* | 0.004±  0  *1* | - | - | 0.004±  0  *1* | - | 0.01 ±  0  *1* | 0.01 ±  0  *1* | 0.01 ±  0.001  *3* | - |
| β-Elemene**^b^** | 1390 | 0.03 ±  0  *1* | 0.03 ±  0.02  *2* | 0.03 ±  0.01  *4* | 0.03 ±  0.01  *6* | - | 0.01 ±  0  *1* | 0.03 ±  0.01  *3* | 0.02 ±  0  *1* | - | - | 0.05 ±  0.01  *3* | 0.02 ±  0.005  *2* | - | - | 0.02 ±  0  *1* | - |
| Longifolene**^b^** | 1402 | 0.01 ±  0.001  *2* | 0.01±  0.003  *3* | 0.01 ±  0.003  *3* | 0.01 ±  0.002  *4* | 0.01 ±  0  *1* | 0.01±  0.001  *2* | 0.01 ±  0.004  *2* | 0.01 ±  0.001  *3* | 0.004 ±  0  *1* | 0.01 ±  0  *1* | 0.003±  0  *1* | 0.01 ±  0.001  *2* | 0.01 ±  0  *1* | 0.004±  0  *1* | 0.01 ±  0  *1* | - |
| α-Gurjunene**^b^** | 1408 | - | - | - | - | - | - | - | - | - | - | - | - | - | - | - | - |
| α-Barbatene**^b^** | 1417 | - | - | - | - | 0.01 ±  0  *1* | - | 0.01 ±  0  *1* | - | - | - | - | - | - | - | - | - |
| β-Caryophyllene**^a^** | 1421 | 0.05 ±  0.02  *3* | 0.07 ±  0.02  *5* | 0.08 ±  0.03  *5* | 0.05 ±  0.02  *6* | 0.06 ±  0.01  *3* | 0.8 ±  0.02  *3* | 0.06 ±  0.02  *6* | 0.06 ±  0.03  *4* | 0.06 ±  0.01  *2* | 0.03 ±  0  *1* | 0.09 ±  0.01  *3* | 0.06 ±  0.04  *2* | 0.11 ±  0.03  *2* | 0.07 ±  0  *1* | 0.10 ±  0.01  *3* | 0.15 ±  0  *1* |
| β-Copaene**^b^** | 1432 | - | - | - | - | - | - | - | - | - | - | - | - | - | - | - | - |
| (*Z*)-Thujopsene**^b^** | 1437 | - | - | - | - | 0.01 ±  0  *1* | - | 0.01 ±  0  *1* | - | - | - | - | - | - | - | - | - |
| (*E*)-β-Farnesene**^a^** | 1452 | 0.02 ±  0.004  *2* | 0.02 ±  0  *1* | 0.02 ±  0.01  *2* | 0.01 ±  0.005  *2* | 0.02 ±  0.01  *2* | 0.002±  0  *1* | 0.05 ±  0.02  *3* | - | - | 0.004±  0  *1* | - | - | 0.01 ±  0.004  *2* | - | 0.01 ±  0.003  *2* | 0.02 ±  0  *1* |
| α-Humulene**^a^** | 1457 | - | 0.01 ±  0.003  *2* | - | - | 0.01 ±  0  *1* | 0.01 ±  0  *1* | - | - | - | - | 0.01 ±  0.001  *2* | - | - | - | 0.01 ±  0  *1* | - |
| α-Curcumene**^b^** | 1481 | - | - | - | - | - | - | - | - | - | - | - | - | - | - | - | - |
| γ-Amorphene**^b^** | 1482 | - | - | - | - | - | - | - | - | - | - | - | - | - | - | - | - |
| Germacrene D**^a^** | 1482 | - | - | - | - | - | - | - | - | - | - | - | - | - | - | - | - |
| β-Chamigrene**^b^** | 1484 | - | - | - | - | 0.02 ±  0  *1* | - | 0.01 ±  0  *1* | - | - | - | - | - | - | - | - | - |
| γ-Humulene**^b^** | 1484 | 0.01 ±  0  *1* | 0.02 ±  0.01  *2* | 0.02 ±  0.01  *2* | 0.01 ±  0.004  *2* | 0.01 ±  0  *1* | 0.01 ±  0  *1* | 0.02 ±  0  *1* | 0.01 ±  0  *2* | - | 0.01 ±  0  *1* | 0.005±  0  *1* | 0.01 ±  0  *1* | 0.02 ±  0  *1* | - | 0.01 ±  0  *1* | - |
| (*Z,E*)-α-Farnesene**^a^** | 1490 | - | - | - | - | - | - | - | - | - | - | - | - | 0.004 ±  0  *1* | - | - | - |
| β-Himachalene**^b^** | 1501 | 0.01 ±  0.003  *2* | 0.02 ±  0.01  *3* | 0.01 ±  0  *1* | 0.02 ±  0.01  *3* | 0.01 ±  0  *1* | 0.01 ±  0  *1* | 0.03 ±  0  *1* | 0.02 ±  0.003  *3* | - | - | 0.01 ±  0  *1* | 0.01 ±  0  *1* | 0.02 ±  0  *1* | - | 0.02 ±  0  *1* | - |
| (*E,E*)-α-Farnesene**^a^** | 1504 | 0.01 ±  0.01  *2* | 0.004±  0.002  *3* | 0.01 ±  0.001  *3* | 0.01 ±  0  *1* | 0.01 ±  0.003  *2* | 0.01 ±  0  *1* | 0.01 ±  0  *1* | 0.01 ±  0.005  *2* | 0.003 ±  0  *1* | 0.01 ±  0  *1* | 0.01 ±  0.004  *3* | - | 0.01 ±  0.001  *2* | - | 0.01 ±  0.001  *2* | - |
| γ-Cadinene**^b^** | 1514 | - | - | - | - | - | - | - | - | - | - | - | - | - | - | - | - |
| δ-Cadinene**^b^** | 1518 | - | - | - | - | - | - | - | - | - | - | - | - | - | - | - | - |
| (*E*)-Calamenene**^b^** | 1522 | - | - | - | - | - | - | - | - | - | - | - | - | - | - | - | - |
| (*E*)-γ-Bisabolene**^b^** | 1549 | - | - | - | - | - | - | - | - | - | - | - | - | - | - | - | - |
| Caryophyllene oxide**^b^** | 1583 | - | - | 0.01 ±  0  *1* | - | - | - | - | - | - | - | - | - | - | - | 0.01 ±  0  *1* | - |
| Longiverbenone**^b^** | 1648 | 0.01 ±  0  *1* | 0.01 ±  0.002  *3* | 0.01 ±  0.0003  *2* | 0.01 ±  0.003  *2* | 0.004±  0  *1* | 0.01 ±  0  *1* | 0.01 ±  0  *1* | 0.01 ±  0  *1* | - | 0.01 ±  0  *1* | - | - | 0.01 ±  0  *1* | 0.003±  0  *1* | 0.004 ±  0  *1* | 0.01 ±  0  *1* |
| Hexahydrofarnesyl acetone**^a^** | 1840 | 0.02 ±  0  *1* | 0.01 ±  0.003  *2* | 0.01 ±  0.003  *3* | 0.01 ±  0.001  *2* | 0.01 ±  0  *1* | 0.01 ±  0.003  *2* | - | 0.01 ±  0  *1* | 0.01 ±  0  *1* | 0.01 ±  0  *1* | 0.004±  0  *1* | - | 0.01 ±  0.003  *2* | 0.01 ±  0  *1* | - | 0.03 ±  0.02  *2* |
|  |  |  |  |  |  |  |  |  |  |  |  |  |  |  |  |  |  |
| ***Homoterpenes*** |  |  |  |  |  |  |  |  |  |  |  |  |  |  |  |  |  |
| (3*E*)-4,8-Dimethyl-  1,3,7-nonatriene**^a^** | 1113 | 0.01 ±  0.002  *3* | 0.02 ±  0.004  *5* | 0.03 ±  0.01  *8* | 0.02 ±  0.005  *5* | 0.02 ±  0.01  *3* | 0.02 ±  0.01  *3* | 0.02 ±  0.01  *4* | 0.03 ±  0.01  *4* | 0.01 ±  0.004  *2* | 0.01 ±  0.001  *3* | 0.03 ±  0.01  *4* | 0.03 ±  0.02  *2* | 0.02 ±  0.003  *3* |  | 0.02 ±  0.01  *5* | 0.01 ±  0  *1* |
| (*E*3,*E*7)-4,8,12-Trimethyltrideca-1,3,7,11-tetraene**^b^** | 1572 | 0.02 ±  0  *1* | 0.02 ±  0  *1* | 0.005±  0.001  *3* | 0.01 ±  0.00  *2* | 0.01 ±  0.01  *2* | - | 0.01 ±  0  *1* | - | - | - | 0.01 ±  0  *1* | - | - | - | 0.004±  0  *1* | - |
|  |  |  |  |  |  |  |  |  |  |  |  |  |  |  |  |  |  |
| ***Trisulfides*** |  |  |  |  |  |  |  |  |  |  |  |  |  |  |  |  |  |
| Dimethyltrisulfide**^a^** | 969 | - | - | - | - | - | - | - | - | - | - | - | - | - | - | - | - |
|  |  |  |  |  |  |  |  |  |  |  |  |  |  |  |  |  |  |
| ***Unknown compounds*** |  |  |  |  |  |  |  |  |  |  |  |  |  |  |  |  |  |
| Unknown1  91,119,134,92,77,105,93,79,  117,41 | 1004 | - | - | - | - | - | - | - | - | 0.02 ±  0  *1* | - | - | 0.04 ±  0  *1* | - | - | - | - |
| Unknown2  69,84,41,81,68,39,53,79,109,  55 | 1072 | 0.02 ±  0.002  *2* | - | 0.01 ±  0.004  *2* | - | 0.01 ±  0  *1* | 0.04 ±  0  *1* | 0.02 ±  0.002  *3* | 0.01 ±  0.002  *2* | 0.01 ±  0  *1* | - | 0.02 ±  0.002  *3* | 0.03 ±  0.01  *3* | - | - | 0.04 ±  0.02  *2* | - |
| Unknown3  91,119,134,77,117,82,79,41,  39,105 | 1079 | 0.01 ±  0.004  *2* | 0.01 ±  0.002  *2* | 0.01 ±  0.001  *5* | 0.01 ±  0.002  *2* | 0.01 ±  0  *1* | 0.01 ±  0  *1* | 0.01 ±  0.003  *3* | 0.01 ±  0  *1* | 0.01 ±  0.0004  *3* | 0.01 ±  0.001  *2* | 0.01 ±  0.002  *3* | 0.01 ±  0  *1* | 0.01 ±  0.004  *2* | - | 0.01 ±  0.001  *4* | - |
| Unknown4  80,107,79,91,122,70,77,105,  93,41 | 1101 | - | 0.01 ±  0  *1* | 0.01 ±  0.0002  *2* | - | - | 0.01 ±  0  *1* | 0.01 ±  0  *1* | - | - | 0.01 ±  0  *1* | 0.01 ±  0  *1* | - | - | - | - | - |
| Unknown5  119,91,134,77,79,105,92,117,  93,39 | 1121 | 0.02 ±  0.003  *6* | 0.02 ±  0.004  *5* | 0.01 ±  0.002  *7* | 0.01 ±  0.002  *6* | 0.01 ±  0.001  *4* | 0.01 ±  0.004  *4* | 0.01 ±  0.004  *5* | 0.01 ±  0.001  *4* | 0.01 ±  0.001  *3* | 0.01 ±  0.002  *4* | 0.02 ±  0.003  *5* | 0.01 ±  0.001  *3* | 0.01 ±  0.004  *3* | 0.002±  0  *1* | 0.02 ±  0.003  *5* | 0.01 ±  0  *1* |
| Unknown6  79,117,43,102,81,90,116,77,  89,91 | 1139 | 0.02 ±  0  *1* | - | 0.01 ±  0  *1* | - | - | - | - | 0.01 ±  0  *1* | - | - | 0.02 ±  0  *1* | - | - | - | - | - |
| Unknown7  94,109,91,79,81,95,59,119,77,67 | 1143 | 0.005±  0  *1* | 0.01 ±  0  *1* | 0.005±  0.002  *3* | 0.005±  0.0003  *2* | 0.004±  0  *1* | 0.004±  0.001  *2* | 0.01 ±  0  *1* | 0.003±  0.0005  *2* | 0.002 ±  0  *1* | - | 0.004±  0  *1* | - | 0.001 ±  0  *1* | - | 0.004 ±  0  *1* | 0.01 ±  0  *1* |
| Unknown8  144,43,101,44,72,73,55,45,  115,42 | 1145 | - | - | - | - | - | - | - | - | - | - | - | - | - | - | - | - |
| Unknown9  70,55,83,69,41,119,134,53,92,81 | 1160 | 0.003±  0  *1* | - | 0.01±  0  *1* | - | 0.01 ±  0.001  *2* | 0.01 ±  0  *1* | 0.004±  0.001  *2* | 0.003±  0  *1* | - | - | 0.01 ±  0.001  *2* | - | - | - | 0.01 ±  0  *1* | - |
| Unknown10  142,129,101,143,75,116,130,  103,117,127 | 1173 | - | - | - | - | - | 0.01 ±  0  *1* | - | - | - | - | - | - | - | 0.01 ±  0  *1* | - | - |
| Unknown11  109,43,81,91,152,79,77,93,67,  119 | 1211 | - | - | - | - | - | - | - | - | - | - | - | - | - | - | - | - |
| Unknown12  150,107,135,91,109,79,39,108,  82,77 | 1339 | 0.003±  0  *1* | - | 0.002±  0  *1* | - | - | - | 0.002±  0  *1* | - | - | - | 0.003±  0  *1* | - | - | - | 0.003±  0  *1* | 0.003±  0  *1* |
| Unknown13  138,83,95,109,193,124,137,57,  41,180 | 1344 | - | - | - | - | - | - | - | - | - | - | - | - | - | - | - | - |
| Unknown14  150,91,107,135,79,77,105,108,  95,109 | 1419 | 0.01 ±  0  *1* | 0.01 ±  0  *1* | 0.01 ±  0  *1* | - | - | 0.01 ±  0  *1* | - | - | - | - | 0.01 ±  0  *1* | - | - | - | - | - |
| Unknown15  161,120,105,119,91,81,121,93,  162,41 | 1419 | - | - | - | - | - | - | - | - | - | - | - | - | - | - | - | - |
| Unknown16  161,105,91,119,93,81,79,77,  133,120 | 1445 | - | - | - | - | - | - | - | - | - | - | - | - | - | - | - | - |
| Unknown17  177,220,135,149,163,67,205,  41,91,136 | 1461 | - | - | - | - | - | - | - | - | - | - | - | - | - | - | - | - |
| Unknown18  161,39,119,105,91,93,79,133,  204,162 | 1475 | - | - | - | - | - | - | - | - | - | - | - | - | - | - | - | - |
| Unknown19  157,69,115,41,158,142,129,91,  132,117 | 1528 | - | - | - | - | - | - | - | - | - | - | - | - | - | - | - | - |
| Unknown20  55,54,84,100,71,57,129,41,111,  56 | 1546 | - | - | - | - | - | - | - | - | - | - | - | - | - | - | - | - |
| Unknown21  69,81,41,79,134,67,53,93,95,107 | 1554 | - | - | - | - | - | - | - | - | - | - | - | - | - | - | - | - |
| Unknown22  107,135,93,91,204,105,41,77,  79,43 | 1595 | 0.01 ±  0  *1* | - | - | - | 0.01 ±  0.001  *2* | - | 0.004 ±  0  *1* | 0.01 ±  0  *1* | - | - | 0.01 ±  0  *1* | - | 0.01 ±  0.003  *2* | - | 0.01 ±  0.002  *2* | - |
| Unknown23  107,135,93,91,204,105,41,77,  79,43 | 1618 | 0.01 ±  0  *1* | - | - | - | 0.004±  0  *1* | - | 0.003 ±  0  *1* | 0.01 ±  0  *1* | - | - | 0.01 ±  0  *1* |  | 0.01 ±  0.001  *2* |  | 0.004 ±  0  *1* |  |
| Unknown24  118,209,224,194,179,119,117,  91,178,210 | 1827 | - | - | - | 0.01 ±  0  *1* | - | 0.01 ±  0  *1* | - | - | - | - | 0.003±  0  *1* | - | - | 0.02 ±  0  *1* | 0.01 ±  0  *1* | 0.02 ±  0  *1* |
| Unknown25  98,85,79,67,55,234,41,112,194,  154 | 2317 | 0.02 ±  0  *1* | 0.01 ±  0  *1* | 0.01 ±  0.0002  *5* | 0.02 ±  0  *1* | - | 0.02 ±  0  *1* | 0.01 ±  0.004  *3* | 0.02 ±  0.01  *2* | 0.02 ±  0.01  *2* | 0.03 ±  0.01  *3* | 0.01 ±  0  *1* | - | 0.01 ±  0  *1* | 0.02 ±  0.01  *2* | 0.04 ±  0  *1* | 0.01 ±  0  *1* |
| Unknown26  91,117,207,194,115,92,193,208,  103,77 | 2434 | - | 0.02 ±  0.01  *2* | - | 0.01 ±  0.0003  *2* | - | 0.01 ±  0  *1* | 0.02 ±  0  *1* | - | - | - | 0.01 ±  0  *1* | - | - | - | - | - |
| Unknown27  40,44,41,43,91,42,79,78,54,55 | 2690 | - | - | - | - | - | - | 0.01 ±  0  *1* | 0.02 ±  0  *1* | - | - | - | - | - | - | - | - |

**Table S2** Results of Permutational multivariate analysis of variance (PERMANOVA) and multivariate homogeneity of group dispersion analysis (PERMDISP) as the deviation from centroid for scent bouquets of *Brassica napus*. Tests are based on Bray-Curtis dissimilarity distances and 9999 permutations. Signiﬁcant *P*-values at *P* ≤ 0.05 are highlighted in bold

1. Main Permutational multivariate analysis of variance (PERMANOVA) of scent bouquets with time points and treatments and their interactions as fixed factors. Sums of Squares (Sums Sq) and Mean Squares (MS) in thousands. *P*(MC): with Monte-Carlo test

| **Factor** | **Df** | **Sums Sq** | **Mean Sq** | **Pseudo*-F*** | **Unique perms** | ***P*(perm)** | ***P*(MC)** |
| --- | --- | --- | --- | --- | --- | --- | --- |
| Time | 2 | 4.2 | 2.1 | 1.87 | 9901 | **0.013** | **0.013** |
| Drought | 1 | 0.8 | 0.8 | 0.75 | 9925 | 0.693 | 0.666 |
| Nitrogen | 1 | 1.5 | 1.5 | 1.30 | 9927 | 0.210 | 0.217 |
| Time × Drought | 2 | 1.4 | 0.7 | 0.62 | 9891 | 0.918 | 0.905 |
| Time × Nitrogen | 2 | 2.2 | 1.1 | 0.97 | 9900 | 0.492 | 0.494 |
| Drought × Nitrogen | 1 | 2.7 | 2.7 | 2.43 | 9922 | **0.008** | **0.010** |
| Time × Drought × Nitrogen | 2 | 1.8 | 0.9 | 0.80 | 9882 | 0.727 | 0.724 |
| Residuals | 81 | 90.4 | 1.1 |  |  |  |  |
| Total | 92 | 106.8 |  |  |  |  |  |

1. Pairwise analysis of variance between the different time points

| **Factor** | ***t*** | **Unique perms** | ***P*(perm)** | ***P*(MC)** |
| --- | --- | --- | --- | --- |
| 0d × 2d | 1.46 | 9925 | **0.020** | **0.023** |
| 0d × 7d | 1.50 | 9930 | **0.013** | **0.021** |
| 2d × 7d | 1.06 | 9927 | 0.325 | 0.334 |

1. Pairwise analysis of variance for interacting effect of watering and nitrogen-treatment

| **Factor** | ***t*** | **Unique perms** | ***P*(perm)** | ***P*(MC)** |
| --- | --- | --- | --- | --- |
| N+: Watered - Drought | 1.68 | 9933 | **0.004** | **0.006** |
| N-: Watered - Drought | 1.04 | 9908 | 0.379 | 0.369 |

1. Multivariate homogeneity of group dispersions analysis between time points and mean distance from centroid with standard error (mean ± SE).

|  | **Df** | ***F*** | ***P*(perm)** | **Distance ± SE** | | |
| --- | --- | --- | --- | --- | --- | --- |
| Groups | 2 | 1.67 | 0.278 | 0d | 2d | 7d |
|  |  |  |  | 31.60 ± 1.03 | 31.38 ± 1.52 | 34.91 ± 1.44 |

1. Multivariate homogeneity of group dispersions analysis between treatments and mean distance from centroid with standard error (mean ± SE)

|  | **Df** | ***F*** | ***P*(perm)** | **Distance ± SE** | | | |
| --- | --- | --- | --- | --- | --- | --- | --- |
| Groups | 3 | 2.47 | 0.133 | N+Drought | N+Control | N-Drought | N-Control |
|  |  |  |  | 32.78 ± 1.76 | 29.57 ± 1.08 | 35.17 ± 1.34 | 33.25 ± 1.45 |

**Table S3** Results of Permutational multivariate analysis of variance (PERMANOVA) and multivariate homogeneity of group dispersion analysis (PERMDISP) as the deviation from centroid for scent bouquets of *Sinapis alba*. Tests are based on Bray-Curtis dissimilarity distances and 9999 permutations. Signiﬁcant *P*-values at *P* ≤ 0.05 are highlighted in bold; underlined *P*-values *P* < 0.10

1. Main Permutational multivariate analysis of variance (PERMANOVA) of scent bouquets with time points and treatments and their interactions as fixed factors. Sums of Squares (Sums Sq) and Mean Squares (MS) in thousands. *P*(MC): with Monte-Carlo test

| **Factor** | **Df** | **Sums Sq** | **Mean Sq** | **Pseudo*-F*** | **Unique perms** | ***P*(perm)** | ***P*(MC)** |
| --- | --- | --- | --- | --- | --- | --- | --- |
| Time | 3 | 5.6 | 1.9 | 2.09 | 9897 | **0.003** | **0.004** |
| Drought | 1 | 3.3 | 3.3 | 3.67 | 9945 | **0.001** | **0.002** |
| Nitrogen | 1 | 2.8 | 2.8 | 3.13 | 9926 | **0.004** | **0.005** |
| Time × Drought | 3 | 4.5 | 1.5 | 1.70 | 9902 | **0.027** | **0.030** |
| Time × Nitrogen | 3 | 3.4 | 1.1 | 1.28 | 9899 | 0.170 | 0.180 |
| Drought × Nitrogen | 1 | 2.0 | 2.0 | 2.27 | 9927 | **0.026** | **0.030** |
| Time × Drought × Nitrogen | 2 | 1.9 | 1.0 | 1.07 | 9914 | 0.382 | 0.368 |
| Residuals | 60 | 53.5 | 0.9 |  |  |  |  |
| Total | 74 | 83.0 |  |  |  |  |  |

1. Pairwise analysis of variance of scent bouquets between time points

| **Factor** | ***t*** | **Unique perms** | ***P*(perm)** | ***P*(MC)** |
| --- | --- | --- | --- | --- |
| 0d × 2d | 1.82 | 9942 | **0.002** | **0.005** |
| 0d × 7d | 1.38 | 9940 | 0.067 | 0.076 |
| 0d × 14d | 1.83 | 9929 | **0.002** | **0.005** |
| 2d × 7d | 1.15 | 9949 | 0.247 | 0.252 |
| 2d × 14d | 0.86 | 9930 | 0.637 | 0.611 |
| 7d × 14d | 1.08 | 9948 | 0.335 | 0.328 |

1. Pairwise analysis of variance of scent bouquets for interacting effect of time and watering

| **Factor** | ***t*** | **Unique perms** | ***P*(perm)** | ***P*(MC)** |
| --- | --- | --- | --- | --- |
| 0d: Watered - Drought | 0.95 | 9937 | 0.493 | 0.475 |
| 2d: Watered - Drought | 1.39 | 9946 | 0.071 | 0.098 |
| 7d: Watered - Drought | 1.66 | 9915 | **0.010** | **0.033** |
| 14d: Watered - Drought | 1.35 | 252 | 0.115 | 0.151 |

1. Pairwise analysis of variance of scent bouquets for interacting effect of watering- and nitrogen-treatment

| **Factor** | ***t*** | **Unique perms** | ***P*(perm)** | ***P*(MC)** |
| --- | --- | --- | --- | --- |
| N+: Watered - Drought | 2.65 | 9941 | **0.0001** | **0.0001** |
| N-: Watered - Drought | 1.45 | 9931 | 0.083 | 0.112 |

1. Multivariate homogeneity of group dispersions analysis between timepoints

|  | **Df** | ***F*** | ***P***  **(perm)** | **Distance ± SE** | | | |
| --- | --- | --- | --- | --- | --- | --- | --- |
| Groups | 3 | 6.19 | 0.005 | 0d | 2d | 7d | 14d |
|  |  |  |  | 26.04 ± 1.22a | 32.60 ± 1.50b | 35.24 ± 1.92b | 31.02 ± 3.14ab |

Lowercase letters denote significant differences of mean distance between timepoints

1. Multivariate homogeneity of group dispersions analysis between watering treatments

|  | **Df** | ***F*** | ***P***  **(perm)** | **Distance ± SE** | |
| --- | --- | --- | --- | --- | --- |
| Groups | 1 | 1.96 | 0.228 | Watered | Drought |
|  |  |  |  | 30.30 ± 0.95a | 32.72 ± 1.52a |

Lowercase letters denote significant differences of mean distance between timepoints

1. Multivariate homogeneity of group dispersions analysis between nitrogen treatments

|  | | **Df** | ***F*** | ***P***  **(perm)** | **Distance ± SE** | |
| --- | --- | --- | --- | --- | --- | --- |
| Groups | | 1 | 0.001 | 0.938 | N- | N+ |
|  |  | |  |  | 31.77 ± 1.87a | 31.53 ± 1.05a |

Lowercase letters denote significant differences of mean distance between timepoints

1. Multivariate homogeneity of group dispersions analysis between the watering treatments and timepoints

|  | **Df** | ***F*** | ***P***  **(perm)** | **Distance ± SE** | | | | | | | | |
| --- | --- | --- | --- | --- | --- | --- | --- | --- | --- | --- | --- | --- |
| Groups | 7 | 2.26 | 0.222 | 0d W | 2d W | 7d W | 14d W | 0d D | 2d D | 7d D | 14d D |  |
|  |  |  |  | 26.01 ± 1.77  aA | 30.32 ± 1.92  aA | 27.44 ± 2.19  aA | 28.16 ± 2.60  aA | 25.56 ± 1.70  aA | 29.13 ± 2.26  abA | 35.49 ± 2.34  bA | 28.11 ± 0  abA |  |

Lowercase letters denote significant differences of mean distance between timepoints within the watering treatments, while capital letters denote significant differences between watering treatments within each time point

1. Multivariate homogeneity of group dispersions analysis between interacting effect of watering- and nitrogen-treatment

|  | **Df** | ***F*** | ***P***  **(perm)** | **Distance ± SE** | | | |
| --- | --- | --- | --- | --- | --- | --- | --- |
| Groups | 3 | 0.55 | 0.791 | N+Drought | N+Control | N-Drought | N-Control |
|  |  |  |  | 30.77 ± 1.0710 | 29.51 ± 1.00 | 33.29 ± 3.43 | 28.80 ± 1.48 |

**Table S4** Results of Permutational multivariate analysis of variance (PERMANOVA) and multivariate homogeneity of group dispersion analysis (PERMDISP) as the deviation from centroid for scent bouquets of *S. arvensis*. Tests are based on Bray-Curtis dissimilarity distances and 9999 permutations. Signiﬁcant *P*-values at *P* ≤ 0.05 are highlighted in bold

1. Main Permutational multivariate analysis of variance (PERMANOVA) of scent bouquets with time points and treatments and their interactions as fixed factors. Sums of Squares (Sums Sq) and Mean Squares (MS) in thousands. *P*(MC): with Monte-Carlo test

| **Factor** | **Df** | **Sums Sq** | **Mean Sq** | **Pseudo*-F*** | **Unique perms** | ***P*(perm)** | ***P*(MC)** |
| --- | --- | --- | --- | --- | --- | --- | --- |
| Time | 3 | 3.4 | 1.1 | 1.34 | 9897 | 0.123 | 0.135 |
| Drought | 1 | 2.5 | 2.5 | 2.98 | 9924 | **0.004** | **0.005** |
| Nitrogen | 1 | 0.8 | 0.8 | 0.95 | 9942 | 0.465 | 0.465 |
| Time × Drought | 3 | 3.5 | 1.2 | 1.35 | 9893 | 0.120 | 0.132 |
| Time × Nitrogen | 3 | 1.7 | 0.6 | 0.68 | 9890 | 0.883 | 0.873 |
| Drought × Nitrogen | 1 | 1.3 | 1.3 | 1.56 | 9922 | 0.123 | 0.137 |
| Time × Drought × Nitrogen | 3 | 1.1 | 0.4 | 0.42 | 9894 | 0.994 | 0.993 |
| Residuals | 71 | 60.7 | 0.9 |  |  |  |  |
| Total | 86 | 74.2 |  |  |  |  |  |

1. Multivariate homogeneity of group dispersions analysis between the watering treatments.

| **Factor** | **Df** | ***F*** | ***P***  **(perm)** | **Distance ± SE** | |
| --- | --- | --- | --- | --- | --- |
| Groups | 1 | 1.25 | 0.309 | Watered | Drought |
|  |  |  |  | 26.89 ± 1.19 | 28.79 ± 1.20 |

**Table S5** SIMPER analysis for scent compounds important for differences between treatments. For each compound: Av.Diss, average dissimilarity of the scent compound between treatments; Diss/SD, dissimilarity between treatment deviation; Contrib %, contribution of single compounds; CumContri%, percentage cumulative contribution to dissimilarity between treatments. Compound cumulative contribution up to 33% to dissimilarity between treatments are shown

|  | ***B. napus*** | | | | | ***S. alba*** | | | | | ***S. arvensis*** | | | | |
| --- | --- | --- | --- | --- | --- | --- | --- | --- | --- | --- | --- | --- | --- | --- | --- |
| **N+Drought**  **N+Control** | **Compound** | **Av.Diss** | **Diss/SD** | **Contrib**  **%** | **Cum**  **Contri%** | **Compound** | **Av.Diss** | **Diss/SD** | **Contrib**  **%** | **Cum**  **Contri%** | **Compound** | **Av.Diss** | **Diss/SD** | **Contrib**  **%** | **Cum**  **Contri%** |
|  | (*3Z*)-3-Hexenyl acetate | 4.07 | 1.16 | 6.66 | 6.66 | (*3Z*)-3-Hexenyl acetate | 4.10 | 0.99 | 6.94 | 6.94 | Hexadecanoic acid | 2.32 | 1.14 | 4.52 | 4.52 |
|  | 6-Methyl-5-heptene-2-one | 3.98 | 1.26 | 6.50 | 13.16 | Benzaldehyde | 3.45 | 1.21 | 5.84 | 12.78 | (*3Z*)-3-Hexenyl acetate | 2.21 | 1.14 | 4.32 | 8.84 |
|  | (*E,E*)-α-Farnesene | 3.21 | 1.27 | 5.24 | 18.40 | Linolenic acid | 2.96 | 1.06 | 5.02 | 17.80 | Linolenic acid | 2.18 | 0.90 | 4.24 | 13.09 |
|  | Linalool | 3.11 | 1.29 | 5.08 | 23.48 | Anisaldehyde | 2.64 | 1.28 | 4.48 | 22.27 | Caryophyllene | 2.10 | 1.25 | 4.09 | 17.17 |
|  | (3*E*)-4,8-dimethyl-1,3,7-Nonatriene | 2.81 | 1.16 | 4.60 | 28.08 | (*E*)-β-Ocimene | 2.53 | 1.23 | 4.28 | 26.55 | α-Longipinene | 2.06 | 1.03 | 4.02 | 21.20 |
|  | Myrcene | 2.80 | 1.20 | 4.58 | 32.66 | Hexadecanoic acid | 1.97 | 0.81 | 3.33 | 29.88 | Benzaldehyde | 2.02 | 0.99 | 3.94 | 25.14 |
|  |  |  |  |  |  | Benzyl alcohol | 1.70 | 1.26 | 2.87 | 32.76 | (*E*)-β-Ocimene | 1.88 | 0.93 | 3.67 | 28.80 |
|  |  |  |  |  |  |  |  |  |  |  | α-Pinene | 1.73 | 0.56 | 3.37 | 32.17 |
| **N+Drought**  **N-Drought** | **Compound** | **Av.Diss** | **Diss/SD** | **Contrib**  **%** | **Cum**  **Contri%** | **Compound** | **Av.Diss** | **Diss/SD** | **Contrib**  **%** | **Cum**  **Contri%** | **Compound** | **Av.Diss** | **Diss/SD** | **Contrib**  **%** | **Cum**  **Contri%** |
|  | 6-Methyl-5-heptene-2-one | 4.15 | 1.21 | 6.14 | 6.14 | (*3Z*)-3-Hexenyl acetate | 4.78 | 1.05 | 6.96 | 6.96 | Hexadecanoic acid | 2.97 | 1.16 | 5.66 | 5.66 |
|  | (*3Z*)-3-Hexenyl acetate | 3.7786 | 1.10 | 5.71 | 11.85 | Benzaldehyde | 4.54 | 1.32 | 6.61 | 13.57 | Linolenic acid | 2.84 | 0.90 | 5.41 | 11.07 |
|  | Linalool | 3.41 | 1.25 | 5.05 | 16.90 | Anisaldehyde | 3.35 | 1.19 | 4.88 | 18.45 | (*3Z*)-3-Hexenyl acetate | 2.54 | 1.10 | 4.84 | 15.90 |
|  | (*E,E*)-α-Farnesene | 3.20 | 1.14 | 4.74 | 21.64 | Linolenic acid | 2.86 | 1.06 | 4.17 | 22.62 | (*E*)-β-Ocimene | 2.49 | 1.19 | 4.75 | 20.65 |
|  | (*E*)-β-Farnesene | 3.04 | 0.68 | 4.50 | 26.14 | Benzyl alcohol | 2.78 | 1.21 | 4.06 | 26.68 | Caryophyllene | 2.09 | 1.23 | 3.98 | 24.64 |
|  | Myrcene | 2.93 | 1.31 | 4.34 | 30.48 | 2-Undecanone | 78 | 0.92 | 4.05 | 30.73 | α-Longipinene | 2.04 | 1.11 | 3.89 | 28.52 |
|  |  |  |  |  |  |  |  |  |  |  | α-Pinene | 2.00 | 0.63 | 3.80 | 32.33 |
| **N+Control**  **N-Control** | **Compound** | **Av.Diss** | **Diss/SD** | **Contrib**  **%** | **Cum**  **Contri%** | **Compound** | **Av.Diss** | **Diss/SD** | **Contrib**  **%** | **Cum**  **Contri%** | **Compound** | **Av.Diss** | **Diss/SD** | **Contrib**  **%** | **Cum**  **Contri%** |
|  | (*3Z*)-3-Hexenyl acetate | 4.35 | 1.11 | 6.92 | 6.92 | Benzaldehyde | 3.06 | 1.16 | 5.44 | 5.44 | Hexadecanoic acid | 2.01 | 1.08 | 4.18 | 4.18 |
|  | 6-Methyl-5-heptene-2-one | 4.34 | 1.25 | 6.90 | 13.82 | Linolenic acid | 2.75 | 1.20 | 4.89 | 10.33 | Caryophyllene | 1.98 | 1.23 | 4.12 | 8.30 |
|  | Linalool | 3.59 | 1.25 | 6.28 | 20.10 | Anisaldehyde | 2.53 | 1.41 | 4.51 | 14.83 | Linolenic acid | 1.89 | 0.95 | 3.92 | 12.22 |
|  | (*E,E*)-α-Farnesene | 3.40 | 1.28 | 5.41 | 25.51 | (*3Z*)-3-Hexenyl acetate | 2.53 | 1.53 | 4.50 | 19.33 | (*E*)-β-Ocimene | 1.79 | 1.05 | 3.71 | 15.92 |
|  | Limonene | 3.37 | 1.20 | 5.35 | 30.86 | (*E*)-β-Farnesene | 1.80 | 1.15 | 3.20 | 22.53 | (*3Z*)-3-Hexenyl acetate | 1.68 | 0.96 | 3.49 | 19.41 |
|  |  |  |  |  |  | Methyl salicylate | 1.77 | 1.22 | 3.16 | 25.69 | α-Longipinene | 1.66 | 0.93 | 3.44 | 22.85 |
|  |  |  |  |  |  | Hexadecanoic acid | 1.59 | 0.73 | 2.83 | 28.52 | Benzaldehyde | 1.58 | 0.97 | 3.27 | 26.12 |
|  |  |  |  |  |  | Benzyl alcohol | 1.56 | 1.19 | 2.78 | 31.30 | Verbenone | 1.54 | 1.07 | 3.20 | 29.31 |
|  |  |  |  |  |  | (3*E*,7*E*)-4,8,12-Trimethyltrideca-1,3,7,11-tetraene | 1.50 | 1.26 | 2.67 | 33.97 | Limonene | 1.42 | 1.27 | 2.95 | 32.26 |
| **N-Drought**  **N-Control** | **Compound** | **Av.Diss** | **Diss/SD** | **Contrib**  **%** | **Cum**  **Contri%** | **Compound** | **Av.Diss** | **Diss/SD** | **Contrib**  **%** | **Cum**  **Contri%** | **Compound** | **Av.Diss** | **Diss/SD** | **Contrib**  **%** | **Cum**  **Contri%** |
|  | 6-Methyl-5-heptene-2-one | 4.57 | 1.21 | 6.73 | 6.73 | (*3Z*)-3-Hexenyl acetate | 3.36 | 1.70 | 5.65 | 5.65 | Hexadecanoic acid | 2.84 | 1.10 | 5.54 | 5.54 |
|  | (*3Z*)-3-Hexenyl acetate | 4.28 | 1.10 | 6.31 | 13.04 | Anisaldehyde | 2.98 | 1.28 | 5.01 | 10.66 | (*E*)-β-Ocimene | 1.24 | 1.25 | 5.19 | 10.72 |
|  | Linalool | 4.23 | 1.19 | 6.23 | 19.27 | Linolenic acid | 2.72 | 1.16 | 4.58 | 15.23 | Linolenic acid | 2.62 | 0.94 | 5.11 | 15.83 |
|  | (*E,E*)-α-Farnesene | 3.26 | 1.10 | 4.80 | 24.08 | Benzyl alcohol | 2.27 | 0.90 | 3.80 | 19.04 | Caryophyllene | 1.98 | 1.18 | 3.86 | 19.69 |
|  | (*E*)-β-Farnesene | 3.12 | 0.66 | 4.60 | 28.68 | (*E*)-β-Farnesene | 1.91 | 1.14 | 3.21 | 22.24 | Verbenone | 1.79 | 1.22 | 3.49 | 23.18 |
|  | Limonene | 3.08 | 1.23 | 4.54 | 33.22 | Methyl salicylate | 1.88 | 1.13 | 3.16 | 25.41 | (*Z*)-β-Ocimene | 1.69 | 1.35 | 3.29 | 26.47 |
|  |  |  |  |  |  | 2-Undecanone | 1.86 | 0.88 | 3.13 | 28.53 | α-Pinene | 1.67 | 0.73 | 3.26 | 29.73 |
|  |  |  |  |  |  | Myrcene | 1.84 | 1.29 | 3.10 | 31.63 | α-Longipinene | 1.67 | 1.04 | 3.25 | 32.98 |

**Table S6** SIMPER analysis for scent compound classes between treatments. For each compound: Av.Diss, average dissimilarity of the scent compound class between treatments; Diss/SD, dissimilarity between treatment deviation; Contrib %, contribution of compound class; CumContri%, percentage cumulative contribution to dissimilarity between treatments. The first three compound classes were chosen for each species.

|  | ***B. napus*** | | | | | ***S. alba*** | | | | | ***S. arvensis*** | | | | |
| --- | --- | --- | --- | --- | --- | --- | --- | --- | --- | --- | --- | --- | --- | --- | --- |
| **N+Drought**  **N+Control** | **Compound**  **class** | **Av.Diss** | **Diss/SD** | **Contrib**  **%** | **Cum**  **Contri%** | **Compound**  **class** | **Av.Diss** | **Diss/SD** | **Contrib**  **%** | **Cum**  **Contri%** | **Compound**  **class** | **Av.Diss** | **Diss/SD** | **Contrib**  **%** | **Cum**  **Contri%** |
|  | fatty acid-derived | 9.84 | 1.29 | 27.04 | 27.04 | fatty acid-derived | 8.87 | 1.32 | 28.88 | 28.88 | fatty acid-derived | 7.04 | 1.26 | 23.36 | 23.36 |
|  | monoterpenes | 7.36 | 1.18 | 20.23 | 47.27 | aromatics | 5.82 | 1.37 | 18.94 | 47.82 | sesquiterpenes | 6.33 | 1.33 | 20.99 | 44.35 |
|  | sesquiterpenes | 7.06 | 1.33 | 19.40 | 66.67 | monoterpenes | 5.55 | 1.27 | 18.07 | 65.89 | aromatics | 5.74 | 1.38 | 19.05 | 63.40 |
| **N+Drought**  **N-Drought** | **Compound**  **class** | **Av.Diss** | **Diss/SD** | **Contrib**  **%** | **Cum**  **Contri%** | **Compound**  **class** | **Av.Diss** | **Diss/SD** | **Contrib**  **%** | **Cum**  **Contri%** | **Compound**  **class** | **Av.Diss** | **Diss/SD** | **Contrib**  **%** | **Cum**  **Contri%** |
|  | fatty acid-derived | 10.03 | 1.29 | 24.82 | 24.82 | fatty acid-derived | 8.52 | 1.35 | 23.26 | 23.26 | fatty acid-derived | 9.14 | 1.40 | 29.29 | 29.29 |
|  | monoterpenes | 8.51 | 1.40 | 21.06 | 45.88 | aromatics | 7.48 | 1.26 | 20.43 | 43.69 | sesquiterpenes | 6.44 | 1.30 | 20.64 | 49.93 |
|  | sesquiterpenes | 8.18 | 1.40 | 20.24 | 66.12 | sesquiterpenes | 5.92 | 1.69 | 16.15 | 59.85 | aromatics | 5.73 | 1.48 | 18.36 | 68.28 |
| **N+Control**  **N-Control** | **Compound**  **class** | **Av.Diss** | **Diss/SD** | **Contrib**  **%** | **Cum**  **Contri%** | **Compound**  **class** | **Av.Diss** | **Diss/SD** | **Contrib**  **%** | **Cum**  **Contri%** | **Compound**  **class** | **Av.Diss** | **Diss/SD** | **Contrib**  **%** | **Cum**  **Contri%** |
|  | fatty acid-derived | 10.49 | 1.37 | 27.94 | 27.94 | fatty acid-derived | 6.23 | 1.50 | 26.10 | 26.10 | fatty acid-derived | 7.62 | 1.37 | 25.36 | 25.36 |
|  | aromatics | 7.07 | 1.14 | 18.85 | 46.80 | aromatics | 4.84 | 1.32 | 20.30 | 46.41 | sesquiterpenes | 5.56 | 1.29 | 18.51 | 43.87 |
|  | sesquiterpenes | 6.71 | 1.37 | 17.89 | 64.68 | sesquiterpenes | 4.29 | 1.46 | 18.00 | 64.41 | aromatics | 5.54 | 1.32 | 18.45 | 62.31 |
| **N-Drought**  **N-Control** | **Compound**  **class** | **Av.Diss** | **Diss/SD** | **Contrib**  **%** | **Cum**  **Contri%** | **Compound**  **class** | **Av.Diss** | **Diss/SD** | **Contrib**  **%** | **Cum**  **Contri%** | **Compound**  **class** | **Av.Diss** | **Diss/SD** | **Contrib**  **%** | **Cum**  **Contri%** |
|  | fatty acid-derived | 10.60 | 1.34 | 26.48 | 26.48 | fatty acid-derived | 6.07 | 1.58 | 24.86 | 24.86 | fatty acid-derived | 10.29 | 1.39 | 32.82 | 32.82 |
|  | aromatics | 7.95 | 1.23 | 19.86 | 46.34 | aromatics | 5.37 | 1.16 | 21.99 | 46.85 | sesquiterpenes | 5.64 | 1.25 | 18.00 | 50.82 |
|  | sesquiterpenes | 7.88 | 1.45 | 19.67 | 66.01 | homoterpenes | 3.65 | 1.37 | 14.95 | 61.81 | aromatics | 5.39 | 1.32 | 17.19 | 68.00 |

**Table S7** Relative amount (mean ± SD) of volatile compounds of *Brassica napus* at the different timepoints (0d, 2d, 7d, 14d), treatments and their interactions (N- Watered, N- Drought, N+ Watered, N+ Drought). “C” = Watered treatment, “D” = Drought-stressed treatment, “N-“ = without additional nitrogen, “N+” = with additional nitrogen. Significance was assessed using Analysis of Deviance Table (Type II tests).

| **Compound** | **Factors** | | | | | | | | | | | |
| --- | --- | --- | --- | --- | --- | --- | --- | --- | --- | --- | --- | --- |
|  | Time | | Watering | | Nitrogen | | Time × Watering | | Time × Nitrogen | | Watering × Nitrogen | |
| (3*E*)-4,8-dimethyl-1,3,7-Nonatriene | 0d | 0.03 ± 0.04a | C | 0.04 ± 0.06a | N- | 0.02 ± 0.03a | 0d.C | 0.04 ± 0.05a | 0d.N- | 0.02 ± 0.03a | C.N- | 0.02 ± 0.04a |
|  | 2d | 0.04 ± 0.06a | D | 0.03 ± 0.04a | N+ | 0.04 ± 0.06b | 2d.C | 0.05 ± 0.08a | 2d.N- | 0.02 ± 0.04a | D.N- | 0.01 ± 0.03a |
|  | 7d | 0.02 ± 0.04a |  | |  | | 7d.C | 0.03 ± 0.05a | 7d.N- | 0.00 ± 0.00a | C.N+ | 0.05 ± 0.07a |
|  | 14d | NA |  |  |  |  | 14d.C | NA | 14d.N- | NA | D.N+ | 0.04 ± 0.04a |
|  |  | |  |  |  |  | 0d.D | 0.03 ± 0.03a | 0d.N+ | 0.04 ± 0.04a |  | |
|  |  |  |  |  |  |  | 2d.D | 0.04 ± 0.05a | 2d.N+ | 0.06 ± 0.07a |  |  |
|  |  |  |  |  |  |  | 7d.D | 0.01 ± 0.02a | 7d.N+ | 0.03 ± 0.04a |  |  |
|  |  |  |  |  |  |  | 14d.D | NA | 14d.N+ | NA |  |  |
| (*3Z*)-3-Hexenyl acetate | 0d | 0.09 ± 0.16a | C | 0.10 ± 0.15a | N- | 0.09 ± 0.14a | 0d.C | 0.11 ± 0.20a | 0d.N- | 0.09 ± 0.16a | C.N- | 0.09 ± 0.15a |
|  | 2d | 0.08 ± 0.11a | D | 0.07 ± 0.10a | N+ | 0.09 ± 0.13a | 2d.C | 0.07 ± 0.10a | 2d.N- | 0.09 ± 0.13a | D.N- | 0.08 ± 0.12a |
|  | 7d | 0.09 ± 0.13a |  | |  | | 7d.C | 0.13 ± 0.14a | 7d.N- | 0.07 ± 0.07a | C.N+ | 0.11 ± 0.16a |
|  | 14d | NA |  |  |  |  | 14d.C | NA | 14d.N- | NA | D.N+ | 0.06 ± 0.09a |
|  |  | |  |  |  |  | 0d.D | 0.07 ± 0.10a | 0d.N+ | 0.09 ± 0.16a |  | |
|  |  |  |  |  |  |  | 2d.D | 0.09 ± 0.11a | 2d.N+ | 0.08 ± 0.09a |  |  |
|  |  |  |  |  |  |  | 7d.D | 0.02 ± 0.03a | 7d.N+ | 0.10 ± 0.14a |  |  |
|  |  |  |  |  |  |  | 14d.D | NA | 14d.N+ | NA |  |  |
| (*E,E*)-α-Farnesene | 0d | 0.09 ± 0.10a | C | 0.05 ± 0.07a | N- | 0.04 ± 0.08a | 0d.C | 0.07 ± 0.07ab | 0d.N- | 0.09 ± 0.11b | C.N- | 0.02 ± 0.04a |
|  | 2d | 0.03 ± 0.05b | D | 0.05 ± 0.09a | N+ | 0.06 ± 0.07b | 2d.C | 0.04 ± 0.07a | 2d.N- | 0.01 ± 0.02a | D.N- | 0.07 ± 0.11ab |
|  | 7d | 0.02 ± 0.05b |  | |  | | 7d.C | 0.04 ± 0.05ab | 7d.N- | 0.00 ± 0.00a | C.N+ | 0.07 ± 0.07b |
|  | 14d | NA |  |  |  |  | 14d.C | NA | 14d.N- | NA | D.N+ | 0.04 ± 0.07ab |
|  |  | |  |  |  |  | 0d.D | 0.10 ± 0.12b | 0d.N+ | 0.09 ± 0.09b |  | |
|  |  |  |  |  |  |  | 2d.D | 0.02 ± 0.03a | 2d.N+ | 0.04 ± 0.06ab |  |  |
|  |  |  |  |  |  |  | 7d.D | 0.00 ± 0.00a | 7d.N+ | 0.03 ± 0.05ab |  |  |
|  |  |  |  |  |  |  | 14d.D | NA | 14d.N+ | NA |  |  |
| (*E*)-β-Farnesene | 0d | 0.05 ± 0.13a | C | 0.05 ± 0.12a | N- | 0.04 ± 0.13a | 0d.C | 0.05 ± 0.13a | 0d.N- | 0.05 ± 0.15a | C.N- | 0.002 ± 0.01a |
|  | 2d | 0.01 ± 0.04a | D | 0.04 ± 0.12a | N+ | 0.04 ± 0.11a | 2d.C | 0.01 ± 0.04a | 2d.N- | 0.02 ± 0.06a | D.N- | 0.09 ± 0.18ab |
|  | 7d | 0.08 ± 0.17a |  | |  | | 7d.C | 0.07 ± 0.16a | 7d.N- | 0.08 ± 0.21a | C.N+ | 0.07 ± 0.14b |
|  | 14d | NA |  |  |  |  | 14d.C | NA | 14d.N- | NA | D.N+ | 0.004 ± 0.02ab |
|  |  | |  |  |  |  | 0d.D | 0.04 ± 0.13a | 0d.N+ | 0.05 ± 0.12a |  | |
|  |  |  |  |  |  |  | 2d.D | 0.01 ± 0.05a | 2d.N+ | 0.01 ± 0.03a |  |  |
|  |  |  |  |  |  |  | 7d.D | 0.08 ± 0.19a | 7d.N+ | 0.07 ± 0.16a |  |  |
|  |  |  |  |  |  |  | 14d.D | NA | 14d.N+ | NA |  |  |
| (*E*)-β-Ocimene | Not emitted | | | | | | | | | | | |
| (*Z*)-β-Ocimene | Not emitted | | | | | | | | | | | |
| 2-Undecanone | Not emitted | | | | | | | | | | | |
| 6-Methyl-5-heptene-2-one | 0d | 0.07 ± 0.09a | C | 0.10 ± 0.12a | N- | 0.09 ± 0.13a | 0d.C | 0.08 ± 0.11a | 0d.N- | 0.07 ± 0.11a | C.N- | 0.12 ± 0.12a |
|  | 2d | 0.13 ± 0.15a | D | 0.09 ± 0.12a | N+ | 0.10 ± 0.11a | 2d.C | 0.13 ± 0.15a | 2d.N- | 0.11 ± 0.15a | D.N- | 0.07 ± 0.14a |
|  | 7d | 0.10 ± 0.10a |  | |  | | 7d.C | 0.10 ± 0.09a | 7d.N- | 0.13 ± 012a | C.N+ | 0.10 ± 0.12a |
|  | 14d | NA |  |  |  |  | 14d.C | NA | 14d.N- | NA | D.N+ | 0.10 ± 0.11a |
|  |  | |  |  |  |  | 0d.D | 0.06 ± 0.07a | 0d.N+ | 0.07 ± 0.08 |  | |
|  |  |  |  |  |  |  | 2d.D | 0.12 ± 0.15a | 2d.N+ | 0.14 ± 0.14a |  |  |
|  |  |  |  |  |  |  | 7d.D | 0.09 ± 0.12a | 7d.N+ | 0.09 ± 0.09a |  |  |
|  |  |  |  |  |  |  | 14d.D | NA | 14d.N+ | NA |  |  |
| α-Pinene | 0d | 0.00 ± 0.00a | C | 0.00 ± 0.00a | N- | 0.00 ± 0.00a | 0d.C | 0.00 ± 0.00a | 0d.N- | 0.00 ± 0.00a | C.N- | 0.00 ± 0.00a |
|  | 2d | 0.0001 ± 0.001a | D | 0.0003 ± 0.002a | N+ | 0.0002 ± 0.001a | 2d.C | 0.00 ± 0.00a | 2d.N- | 0.00 ± 0.00a | D.N- | 0.00 ± 0.00a |
|  | 7d | 0.0005 ± 0.00a |  | |  | | 7d.C | 0.00 ± 0.00a | 7d.N- | 0.00 ± 0.00a | C.N+ | 0.00 ± 0.00a |
|  | 14d | NA |  |  |  |  | 14d.C | NA | 14d.N- | NA | D.N+ | 0.001 ± 0.002a |
|  |  | |  |  |  |  | 0d.D | 0.00 ± 0.00a | 0d.N+ | 0.00 ± 0.00a |  | |
|  |  |  |  |  |  |  | 2d.D | 0.0002 ± 0.001a | 2d.N+ | 0.0002 ± 0.001a |  |  |
|  |  |  |  |  |  |  | 7d.D | 0.001 ± 0.004a | 7d.N+ | 0.001 ± 0.003a |  |  |
|  |  |  |  |  |  |  | 14d.D | NA | 14d.N+ | NA |  |  |
| α-Longipinene | Not emitted | | | | | | | | | | | |
| p-Anisaldehyde | 0d | 0.002 ± 0.01a | C | 0.001 ± 0.004a | N- | 0.0004 ± 0.002a | 0d.C | 0.002 ± 0.01a | 0d.N- | 0.00 ± 0.00a | C.N- | 0.00 ± 0.00a |
|  | 2d | 0.0004 ± 0.002a | D | 0.001 ± 0.01a | N+ | 0.001 ± 0.01a | 2d.C | 0.00 ± 0.00a | 2d.N- | 0.001 ± 0.003a | D.N- | 0.001 ± 0.003a |
|  | 7d | 0.00 ± 0.00a |  | |  | | 7d.C | 0.00 ± 0.00a | 7d.N- | 0.00 ± 0.00a | C.N+ | 0.001 ± 0.005a |
|  | 14d | NA |  |  |  |  | 14d.C | NA | 14d.N- | NA | D.N+ | 0.001 ± 0.01a |
|  |  | |  |  |  |  | 0d.D | 0.002 ± 0.01a | 0d.N+ | 0.003 ± 0.01a |  | |
|  |  |  |  |  |  |  | 2d.D | 0.001 ± 0.003a | 2d.N+ | 0.00 ± 0.00a |  |  |
|  |  |  |  |  |  |  | 7d.D | 0.00 ± 0.00a | 7d.N+ | 0.00 ± 0.00a |  |  |
|  |  |  |  |  |  |  | 14d.D | NA | 14d.N+ | NA |  |  |
| Benzaldehyde | 0d | 0.02 ± 0.05a | C | 0.02 ± 0.05a | N- | 0.01 ± 0.04a | 0d.C | 0.03 ±0.07a | 0d.N- | 0.01 ± 0.03a | C.N- | 0.01 ± 0.03a |
|  | 2d | 0.04 ± 0.12a | D | 0.04 ± 0.11a | N+ | 0.04 ± 0.10a | 2d.C | 0.02 ± 0.05a | 2d.N- | 0.02 ± 0.04a | D.N- | 0.01 ± 0.04a |
|  | 7d | 0.03 ± 0.05a |  | |  | | 7d.C | 0.01 ±0.03a | 7d.N- | 0.02 ± 0.04a | C.N+ | 0.02 ± 0.06a |
|  | 14d | NA |  |  |  |  | 14d.C | NA | 14d.N- | NA | D.N+ | 0.05 ± 0.13a |
|  |  | |  |  |  |  | 0d.D | 0.01 ±0.03a | 0d.N+ | 0.03 ± 0.06a |  | |
|  |  |  |  |  |  |  | 2d.D | 0.06 ± 0.16a | 2d.N+ | 0.05 ± 0.15a |  |  |
|  |  |  |  |  |  |  | 7d.D | 0.06 ± 0.07a | 7d.N+ | 0.03 ± 0.06a |  |  |
|  |  |  |  |  |  |  | 14d.D | NA | 14d.N+ | NA |  |  |
| Benzyl alcohol | 0d | 0.003 ± 0.01a | C | 0.01 ± 0.03a | N- | 0.02 ± 0.06a | 0d.C | 0.004 ± 0.01a | 0d.N- | 0.00 ± 0.00a | C.N- | 0.02 ± 0.05a |
|  | 2d | 0.02 ± 0.06a | D | 0.01 ± 0.04a | N+ | 0.01 ± 0.02a | 2d.C | 0.01 ± 0.05a | 2d.N- | 0.04 ± 0.08a | D.N- | 0.02 ± 0.07a |
|  | 7d | 0.01 ± 0.02a |  |  |  |  | 7d.C | 0.004 ± 0.02a | 7d.N- | 0.01 ± 0.02a | C.N+ | 0.002 ± 0.01a |
|  | 14d | NA |  |  |  |  | 14d.C | NA | 14d.N- | NA | D.N+ | 0.01 ± 0.03a |
|  |  |  |  |  |  |  | 0d.D | 0.003 ± 0.01a | 0d.N+ | 0.01 ± 0.01a |  |  |
|  |  |  |  |  |  |  | 2d.D | 0.02 ± 0.07a | 2d.N+ | 0.01 ± 0.03a |  |  |
|  |  |  |  |  |  |  | 7d.D | 0.02 ± 0.02a | 7d.N+ | 0.01 ± 0.02a |  |  |
|  |  |  |  |  |  |  | 14d.D | NA | 14d.N+ | NA |  |  |
| Caryophyllene | Not emitted | | | | | | | | | | | |
| Hexadecanoic acid | 0d | 0.05 ± 0.14a | C | 0.03 ± 0.10a | N- | 0.03 ± 0.10a | 0d.C | 0.03 ± 0.12a | 0d.N- | 0.05 ± 0.14a | C.N- | 0.04 ± 0.13a |
|  | 2d | 0.02 ± 0.06a | D | 0.05 ± 0.12a | N+ | 0.04 ± 0.12a | 2d.C | 0.01 ± 0.05a | 2d.N- | 0.02 ± 0.06a | D.N- | 0.02 ± 0.03a |
|  | 7d | 0.05 ± 0.13a |  | |  | | 7d.C | 0.05 ± 0.13a | 7d.N- | 0.01 ± 0.02a | C.N+ | 0.02 ± 0.09a |
|  | 14d | NA |  |  |  |  | 14d.C | NA | 14d.N- | NA | D.N+ | 0.06 ± 0.15a |
|  |  | |  |  |  |  | 0d.D | 0.07 ± 0.16a | 0d.N+ | 0.05 ± 0.15a |  | |
|  |  |  |  |  |  |  | 2d.D | 0.02 ± 0.06a | 2d.N+ | 0.01 ± 0.05a |  |  |
|  |  |  |  |  |  |  | 7d.D | 0.05 ± 0.14a | 7d.N+ | 0.07 ± 0.15a |  |  |
|  |  |  |  |  |  |  | 14d.D | NA | 14d.N+ | NA |  |  |
| Limonene | 0d | 0.06 ± 0.05a | C | 0.09 ± 0.08a | N- | 0.06 ± 0.06a | 0d.C | 0.06 ± 0.05a | 0d.N- | 0.04 ± 0.04a | C.N- | 0.07 ± 0.07a |
|  | 2d | 0.08 ± 0.07a | D | 0.07 ± 0.06a | N+ | 0.09 ± 0.08a | 2d.C | 0.09 ± 0.07a | 2d.N- | 0.05 ± 0.06a | D.N- | 0.05 ± 0.05a |
|  | 7d | 0.10 ± 0.10a |  | |  | | 7d.C | 0.11 ± 0.11a | 7d.N- | 0.11 ± 0.08a | C.N+ | 0.10 ± 0.08a |
|  | 14d | NA |  |  |  |  | 14d.C | NA | 14d.N- | NA | D.N+ | 0.08 ± 0.07a |
|  |  | |  |  |  |  | 0d.D | 0.06 ± 0.05a | 0d.N+ | 0.07 ± 0.05a |  | |
|  |  |  |  |  |  |  | 2d.D | 0.07 ± 0.06a | 2d.N+ | 0.10 ± 0.07a |  |  |
|  |  |  |  |  |  |  | 7d.D | 0.08 ± 0.09a | 7d.N+ | 0.10 ± 0.11a |  |  |
|  |  |  |  |  |  |  | 14d.D | NA | 14d.N+ | NA |  |  |
| Linalool | 0d | 0.11 ± 0.11a | C | 0.08 ± 0.10a | N- | 0.09 ± 0.12a | 0d.C | 0.12 ± 0.13a | 0d.N- | 0.13 ± 0.14a | C.N- | 0.11 ± 0.14a |
|  | 2d | 0.08 ± 0.08ab | D | 0.08 ± 0.08a | N+ | 0.08 ± 0.07a | 2d.C | 0.08 ± 0.09a | 2d.N- | 0.06 ± 0.09a | D.N- | 0.07 ± 0.09a |
|  | 7d | 0.03 ± 0.05b |  | |  | | 7d.C | 0.03 ± 0.06a | 7d.N- | 0.06 ± 0.09a | C.N+ | 0.07 ± 0.07a |
|  | 14d | NA |  |  |  |  | 14d.C | NA | 14d.N- | NA | D.N+ | 0.08 ± 0.07a |
|  |  | |  |  |  |  | 0d.D | 0.10 ± 0.09a | 0d.N+ | 0.10 ± 0.08a |  | |
|  |  |  |  |  |  |  | 2d.D | 0.07 ± 0.07a | 2d.N+ | 0.09 ± 0.07a |  |  |
|  |  |  |  |  |  |  | 7d.D | 0.03 ± 0.04a | 7d.N+ | 0.02 ± 0.03a |  |  |
|  |  |  |  |  |  |  | 14d.D | NA | 14d.N+ | NA |  |  |
| Linolenic acid | 0d | 0.003 ± 0.02a | C | 0.002 ± 0.02a | N- | 0.005 ±0.02a | 0d.C | 0.01 ± 0.03a | 0d.N- | 0.01 ± 0.03a | C.N- | 0.01 ± 0.03a |
|  | 2d | 0.002 ± 0.01a | D | 0.002 ± 0.01a | N+ | 0.001 ± 0.01a | 2d.C | 0.00 ± 0.00a | 2d.N- | 0.003 ± 0.01a | D.N- | 0.003 ± 0.01a |
|  | 7d | 0.001 ± 0.01a |  | |  | | 7d.C | 0.00 ± 0.00a | 7d.N- | 0.00 ± 0.00a | C.N+ | 0.00 ± 0.00a |
|  | 14d | NA |  |  |  |  | 14d.C | NA | 14d.N- | NA | D.N+ | 0.002 ± 0.01a |
|  |  | |  |  |  |  | 0d.D | 0.00 ± 0.00a | 0d.N+ | 0.00 ± 0.00a |  | |
|  |  |  |  |  |  |  | 2d.D | 0.004 ± 0.01a | 2d.N+ | 0.001 ± 0.01a |  |  |
|  |  |  |  |  |  |  | 7d.D | 0.003 ± 0.01a | 7d.N+ | 0.002 ± 0.01a |  |  |
|  |  |  |  |  |  |  | 14d.D | NA | 14d.N+ | NA |  |  |
| Methyl salicylate | 0d | 0.02 ± 0.04a | C | 0.01 ± 0.02a | N- | 0.01 ± 0.04a | 0d.C | 0.01 ± 0.03a | 0d.N- | 0.02 ± 0.06a | C.N- | 0.004 ± 0.01a |
|  | 2d | 0.01 ± 0.01a | D | 0.01 ± 0.04a | N+ | 0.01 ± 0.02a | 2d.C | 0.01 ± 0.02a | 2d.N- | 0.004 ± 0.01a | D.N- | 0.02 ± 0.06a |
|  | 7d | 0.003 ± 0.01a |  | |  | | 7d.C | 0.004 ± 0.01a | 7d.N- | 0.01 ± 0.02a | C.N+ | 0.01 ± 0.03a |
|  | 14d | NA |  |  |  |  | 14d.C | NA | 14d.N- | NA | D.N+ | 0.003 ± 0.01a |
|  |  | |  |  |  |  | 0d.D | 0.02 ± 0.05a | 0d.N+ | 0.01 ± 0.03a |  | |
|  |  |  |  |  |  |  | 2d.D | 0.004 ± 0.01a | 2d.N+ | 0.01 ± 0.02a |  |  |
|  |  |  |  |  |  |  | 7d.D | 0.002 ±0.01a | 7d.N+ | 0.002 ± 0.004a |  |  |
|  |  |  |  |  |  |  | 14d.D | NA | 14d.N+ | NA |  |  |
| Myrcene | 0d | 0.11 ± 0.08ab | C | 0.12 ± 0.08a | N- | 0.09 ± 0.08a | 0d.C | 0.13 ± 0.08a | 0d.N- | 0.10 ± 0.08a | C.N- | 0.11 ± 0.08a |
|  | 2d | 0.14 ± 0.09a | D | 0.11 ± 0.08a | N+ | 0.13 ± 0.09a | 2d.C | 0.14 ± 0.09a | 2d.N- | 0.10 ± 0.08a | D.N- | 0.08 ± 0.06a |
|  | 7d | 0.07 ± 0.06b |  | |  | | 7d.C | 0.08 ± 0.07a | 7d.N- | 0.07 ± 0.05a | C.N+ | 0.13 ± 0.08a |
|  | 14d | NA |  |  |  |  | 14d.C | NA | 14d.N- | NA | D.N+ | 0.12 ± 0.09a |
|  |  | |  |  |  |  | 0d.D | 0.10 ± 0.07a | 0d.N+ | 0.12 ± 0.07a |  | |
|  |  |  |  |  |  |  | 2d.D | 0.13 ± 0.10a | 2d.N+ | 0.17 ± 0.09 |  |  |
|  |  |  |  |  |  |  | 7d.D | 0.06 ±0.06ab | 7d.N+ | 0.07 ± 0.07a |  |  |
|  |  |  |  |  |  |  | 14d.D | NA | 14d.N+ | NA |  |  |
| (*E*3,*E*7)-4,8,12-Trimethyltrideca-1,3,7,11-tetraene | 0d | 0.01 ± 0.02a | C | 0.01 ± 0.01a | N- | 0.002 ± 0.01a | 0d.C | 0.01 ± 0.02a | 0d.N- | 0.004 ± 0.01ab | C.N- | 0.00 ±0.00a |
|  | 2d | 0.001 ±0.004b | D | 0.005 ± 0.01a | N+ | 0.01 ± 0.02a | 2d.C | 0.001 ±0.01a | 2d.N- | 0.00 ±0.00a | D.N- | 0.004 ± 0.01a |
|  | 7d | 0.004 ± 0.01ab |  | |  | | 7d.C | 0.01 ± 0.02a | 7d.N- | 0.00 ±0.00ab | C.N+ | 0.01 ± 0.02a |
|  | 14d | NA |  |  |  |  | 14d.C | NA | 14d.N- | NA | D.N+ | 0.01 ± 0.01a |
|  |  | |  |  |  |  | 0d.D | 0.01 ± 0.02a | 0d.N+ | 0.01 ± 0.02b |  | |
|  |  |  |  |  |  |  | 2d.D | 0.00 ± 0.00a | 2d.N+ | 0.001 ± 0.01a |  |  |
|  |  |  |  |  |  |  | 7d.D | 0.00 ±0.00a | 7d.N+ | 0.01 ± 0.02ab |  |  |
|  |  |  |  |  |  |  | 14d.D | NA | 14d.N+ | NA |  |  |
| Verbenone | Not emitted | | | | | | | | | | | |

Lowercase letters denote significant differences within factors.

**Table S8** Relative amount (mean ± SD) of volatile compounds of *Sinapis alba* in the different timepoints (0d, 2d, 7d, 14d), treatments and their interactions. “C” = Watered treatment, “D” = Drought-stressed treatment, “N-“ = without additional nitrogen, “N+” = with additional nitrogen. Significance was assessed using Analysis of Deviance Table (Type II tests).

| **Compound** | **Factors** | | | | | | | | | | | |
| --- | --- | --- | --- | --- | --- | --- | --- | --- | --- | --- | --- | --- |
|  | Time | | Watering | | Nitrogen | | Time × Watering | | Time × Nitrogen | | Watering × Nitrogen | |
| (3*E*)-4,8-dimethyl-1,3,7-Nonatriene | 0d | 0.01 ± 0.02a | C | 0.02 ± 0.02a | N- | 0.03 ± 0.03a | 0d.C | 0.01 ± 0.01a | 0d.N- | 0.03 ± 0.03a | C.N- | 0.02 ± 0.02a |
|  | 2d | 0.02 ± 0.02a | D | 0.01 ± 0.02a | N+ | 0.01 ± 0.02a | 2d.C | 0.02 ± 0.02a | 2d.N- | 0.04 ± 0.03a | D.N- | 0.04 ± 0.03a |
|  | 7d | 0.02 ± 0.03a |  | |  | | 7d.C | 0.03 ± 0.02a | 7d.N- | 0.004 ± 0.01a | C.N+ | 0.02 ± 0.02a |
|  | 14d | 0.01 ± 0.02a |  |  |  |  | 14d.C | 0.01 ± 0.01a | 14d.N- | 0.00 ± NAa | D.N+ | 0.01 ± 0.02a |
|  |  | |  |  |  |  | 0d.D | 0.01 ± 0.02a | 0d.N+ | 0.01 ± 0.01a |  | |
|  |  |  |  |  |  |  | 2d.D | 0.01 ± 0.02a | 2d.N+ | 0.01 ± 0.01a |  |  |
|  |  |  |  |  |  |  | 7d.D | 0.01 ± 0.03a | 7d.N+ | 0.02 ± 0.03a |  |  |
|  |  |  |  |  |  |  | 14d.D | 0.02 ± 0.02a | 14d.N+ | 0.02 ± 0.01a |  |  |
| (*3Z*)-3-Hexenyl acetate | 0d | 0.02 ± 0.03a | C | 0.03 ± 0.07a | N- | 0.07 ± 0.11a | 0d.C | 0.02 ± 0.03a | 0d.N- | 0.03 ± 0.05ab | C.N- | 0.07 ± 0.07ab |
|  | 2d | 0.14 ± 0.25b | D | 0.15 ± 0.23b | N+ | 0.09 ±0.18a | 2d.C | 0.02 ± 0.06a | 2d.N- | 0.17 ± 0.16ab | D.N- | 0.08 ± 0.17ab |
|  | 7d | 0.09 ± 0.16ab |  | |  | | 7d.C | 0.01 ± 0.02a | 7d.N- | 0.01 ± 0.01ab | C.N+ | 0.02 ± 0.06a |
|  | 14d | 0.15 ± 0.14ab |  |  |  |  | 14d.C | 0.10 ± 0.11ab | 14d.N- | 0.07 ± NAab | D.N+ | 0.17 ± 0.24b |
|  |  | |  |  |  |  | 0d.D | 0.02 ± 0.02a | 0d.N+ | 0.01 ± 0.02a |  | |
|  |  |  |  |  |  |  | 2d.D | 0.29 ± 0.32b | 2d.N+ | 0.13 ± 0.27ab |  |  |
|  |  |  |  |  |  |  | 7d.D | 0.16 ± 0.21ab | 7d.N+ | 0.10 ± 0.17ab |  |  |
|  |  |  |  |  |  |  | 14d.D | 0.32 ± 0.02ab | 14d.N+ | 0.16 ± 0.15b |  |  |
| (*E,E*)-α-Farnesene | 0d | 0.01 ± 0.02a | C | 0.02 ± 0.02a | N- | 0.01 ± 0.02a | 0d.C | 0.02 ± 0.02a | 0d.N- | 0.01 ± 0.02a | C.N- | 0.02 ± 0.02a |
|  | 2d | 0.02 ± 0.03a | D | 0.01 ± 0.01b | N+ | 0.02 ± 0.02a | 2d.C | 0.03 ± 0.03a | 2d.N- | 0.02 ± 0.03a | D.N- | 0.002 ± 0.004a |
|  | 7d | 0.01 ± 0.02a |  | |  | | 7d.C | 0.02 ± 0.02a | 7d.N- | 0.01 ± 0.01a | C.N+ | 0.02 ± 0.02a |
|  | 14d | 0.02 ± 0.02a |  |  |  |  | 14d.C | 0.02 ± 0.02a | 14d.N- | 0.01 ± NAa | D.N+ | 0.01 ± 0.01a |
|  |  | |  |  |  |  | 0d.D | 0.01 ± 0.02a |  | |  | |
|  |  |  |  |  |  |  | 2d.D | 0.01 ± 0.01a |  |  |  |  |
|  |  |  |  |  |  |  | 7d.D | 0.01 ± 0.01a |  |  |  |  |
|  |  |  |  |  |  |  | 14d.D | 0.01 ± 0.01a |  |  |  |  |
| (*E*)-β-Farnesene | 0d | 0.01 ± 0.02a | C | 0.02 ± 0.04a | N- | 0.04 ± 0.05a | 0d.C | 0.01 ± 0.02a | 0d.N- | 0.02 ± 0.02a | C.N- | 0.04 ± 0.05a |
|  | 2d | 0.01 ± 0.03ab | D | 0.02 ± 0.04a | N+ | 0.01 ± 0.04b | 2d.C | 0.01 ± 0.01a | 2d.N- | 0.05 ± 0.06a | D.N- | 0.03 ± 0.05a |
|  | 7d | 0.02 ± 0.04ab |  | |  | | 7d.C | 0.01 ± 0.01a | 7d.N- | 0.01 ± 0.01a | C.N+ | 0.02 ± 0.04a |
|  | 14d | 0.07 ± 0.07b |  |  |  |  | 14d.C | 0.08 ± 0.07b | 14d.N- | 0.16 ± NAa | D.N+ | 0.01 ± 0.03a |
|  |  | |  |  |  |  | 0d.D | 0.004 ± 0.01a | 0d.N+ | 0.003 ± 0.01a |  | |
|  |  |  |  |  |  |  | 2d.D | 0.02 ± 0.04ab | 2d.N+ | 0.005 ± 0.01a |  |  |
|  |  |  |  |  |  |  | 7d.D | 0.03 ± 0.05ab | 7d.N+ | 0.02 ± 0.04a |  |  |
|  |  |  |  |  |  |  | 14d.D | 0.02 ± 0.02ab | 14d.N+ | 0.05 ± 0.07a |  |  |
| (*E*)-β-Ocimene | 0d | 0.11 ± 0.06a | C | 0.14 ± 0.08a | N- | 0.11 ± 0.05a | 0d.C | 0.09 ± 0.05ab | 0d.N- | 0.13 ± 0.05a | C.N- | 0.11 ± 0.03ab |
|  | 2d | 0.11 ± 0.08a | D | 0.08 ± 0.07b | N+ | 0.11 ± 0.08a | 2d.C | 0.15 ± 0.08b | 2d.N- | 0.11 ± 0.02a | D.N- | 0.11 ± 0.08ab |
|  | 7d | 0.10 ± 0.10a |  | |  | | 7d.C | 0.19 ± 0.08b | 7d.N- | 0.07 ± 0.10a | C.N+ | 0.15 ± 0.08b |
|  | 14d | 0.14 ± 0.08a |  |  |  |  | 14d.C | 0.15 ± 0.07ab | 14d.N- | 0.07 ± NAa | D.N+ | 0.08 ± 0.07a |
|  |  | |  |  |  |  | 0d.D | 0.13 ± 0.06b | 0d.N+ | 0.11 ± 0.06a |  | |
|  |  |  |  |  |  |  | 2d.D | 0.07 ± 0.07ab | 2d.N+ | 0.11 ± 0.09a |  |  |
|  |  |  |  |  |  |  | 7d.D | 0.03 ± 0.04a | 7d.N+ | 0.11 ± 0.11a |  |  |
|  |  |  |  |  |  |  | 14d.D | 0.10 ± 0.15ab | 14d.N+ | 0.15 ± 0.08a |  |  |
| (*Z*)-β-Ocimene | 0d | 0.01 ± 0.01a | C | 0.02 ± 0.02a | N- | 0.02 ± 0.01a | 0d.C | 0.01 ± 0.01a | 0d.N- | 0.02 ± 0.01a | C.N- | 0.02 ± 0.01ab |
|  | 2d | 0.01 ± 0.01a | D | 0.01 ± 0.01a | N+ | 0.01 ± 0.02a | 2d.C | 0.02 ± 0.01a | 2d.N- | 0.01 ± 0.004a | D.N- | 0.02 ± 0.02ab |
|  | 7d | 0.02 ± 0.03a |  | |  | | 7d.C | 0.03 ± 0.03a | 7d.N- | 0.01 ± 0.02a | C.N+ | 0.02 ± 0.02a |
|  | 14d | 0.02 ± 0.01a |  |  |  |  | 14d.C | 0.02 ± 0.01a | 14d.N- | 0.01 ± NAa | D.N+ | 0.01 ± 0.01b |
|  |  | |  |  |  |  | 0d.D | 0.02 ± 0.02a | 0d.N+ | 0.01 ± 0.01a |  | |
|  |  |  |  |  |  |  | 2d.D | 0.01 ± 0.01a | 2d.N+ | 0.01 ± 0.02a |  |  |
|  |  |  |  |  |  |  | 7d.D | 0.002 ± 0.004a | 7d.N+ | 0.02 ± 0.03a |  |  |
|  |  |  |  |  |  |  | 14d.D | 0.02 ± 0.02a | 14d.N+ | 0.02 ± 0.01a |  |  |
| 2-Undecanone | 0d | 0.02 ± 0.01a | C | 0.02 ± 0.21a | N- | 0.06 ± 0.08a | 0d.C | 0.01 ± 0.01a | 0d.N- | 0.03 ± 0.03ab | C.N- | 0.03 ± 0.02a |
|  | 2d | 0.02 ± 0.02ab | D | 0.03 ± 0.05a | N+ | 0.02 ± 0.01b | 2d.C | 0.02 ± 0.02ab | 2d.N- | 0.04 ± 0.01bc | D.N- | 0.09 ± 0.12a |
|  | 7d | 0.03 ± 0.07b |  | |  | | 7d.C | 0.02 ± 0.02ab | 7d.N- | 0.16 ± 0.20c | C.N+ | 0.02 ± 0.01b |
|  | 14d | 0.02 ± 0.02ab |  |  |  |  | 14d.C | 0.03 ± 0.02ab | 14d.N- | 0.08 ± NAabc | D.N+ | 0.01 ± 0.02b |
|  |  | |  |  |  |  | 0d.D | 0.02 ± 0.02ab | 0d.N+ | 0.01 ± 0.01a |  | |
|  |  |  |  |  |  |  | 2d.D | 0.02 ± 0.02ab | 2d.N+ | 0.02 ± 0.01ab |  |  |
|  |  |  |  |  |  |  | 7d.D | 0.05 ± 0.10b | 7d.N+ | 0.02 ± 0.02ab |  |  |
|  |  |  |  |  |  |  | 14d.D | 0.01 ± 0.02ab | 14d.N+ | 0.02 ± 0.01ab |  |  |
| 6-Methyl-5-heptene-2-one | 0d | 0.004 ± 0.01a | C | 0.005 ± 0.01a | N- | 0.02 ± 0.03a | 0d.C | 0.004 ± 0.01a | 0d.N- | 0.002 ± 0.004a | C.N- | 0.01 ± 0.02ab |
|  | 2d | 0.01 ± 0.02b | D | 0.02 ± 0.04b | N+ | 0.01 ± 0.03a | 2d.C | 0.01 ± 0.02a | 2d.N- | 0.05 ± 0.04b | D.N- | 0.02 ± 0.04ab |
|  | 7d | 0.03 ± 0.06ab |  | |  | | 7d.C | 0.01 ± 0.01a | 7d.N- | 0.01 ± 0.01ab | C.N+ | 0.003 ± 0.01a |
|  | 14d | 0.01 ± 0.02ab |  |  |  |  | 14d.C | 0.001 ± 0.002a | 14d.N- | 0.00 ± NAab | D.N+ | 0.02 ± 0.05b |
|  |  | |  |  |  |  | 0d.D | 0.005 ± 0.01a | 0d.N+ | 0.01 ± 0.01a |  | |
|  |  |  |  |  |  |  | 2d.D | 0.02 ± 0.03a | 2d.N+ | 0.005 ± 0.01a |  |  |
|  |  |  |  |  |  |  | 7d.D | 0.04 ± 0.08a | 7d.N+ | 0.03 ± 0.06ab |  |  |
|  |  |  |  |  |  |  | 14d.D | 0.05 ± 0.01a | 14d.N+ | 0.01 ± 0.02ab |  |  |
| α-Longipinene | 0d | 0.00 ± 0.00a | C | 0.00 ± 0.00a | N- | 0.00 ± 0.00a | 0d.C | 0.00 ± 0.00a | 0d.N- | 0.00 ± 0.00a | C.N- | 0.00 ± 0.00a |
|  | 2d | 0.00 ± 0.00a | D | 0.0001 ± 0.001a | N+ | 0.00 ± 0.00a | 2d.C | 0.00 ± 0.00a | 2d.N- | 0.00 ± 0.00a | D.N- | 0.00 ± 0.00a |
|  | 7d | 0.001 ± 0.001a |  | |  | | 7d.C | 0.00 ± 0.00a | 7d.N- | 0.00 ± 0.00a | C.N+ | 0.00 ± 0.00a |
|  | 14d | 0.00 ± 0.00a |  |  |  |  | 14d.C | 0.00 ± 0.00a | 14d.N- | 0.00 ± NAa | D.N+ | 0.0001 ± 0.001a |
|  |  | |  |  |  |  | 0d.D | 0.00 ± 0.00a | 0d.N+ | 0.00 ± 0.00a |  | |
|  |  |  |  |  |  |  | 2d.D | 0.00 ± 0.00a | 2d.N+ | 0.00 ± 0.00a |  |  |
|  |  |  |  |  |  |  | 7d.D | 0.0004 ± 0.001a | 7d.N+ | 0.0002 ± 0.001a |  |  |
|  |  |  |  |  |  |  | 14d.D | 0.00 ± 0.00a | 14d.N+ | 0.00 ± 0.00a |  |  |
| α-Pinene | Not emitted | | | | | | | | | | | |
| p-Anisaldehyde | 0d | 0.21 ± 0.11a | C | 0.13 ± 0.10a | N- | 0.11 ± 0.11a | 0d.C | 0.21 ± 0.11a | 0d.N- | 0.17 ± 0.12bc | C.N- | 0.13 ± 0.13ab |
|  | 2d | 0.11 ± 0.10b | D | 0.14 ± 0.12a | N+ | 0.14 ± 0.11b | 2d.C | 0.10 ± 0.08bc | 2d.N- | 0.05 ± 0.09ab | D.N- | 0.07 ± 0.07a |
|  | 7d | 0.09 ± 0.07b |  | |  | | 7d.C | 0.11 ± 0.06abc | 7d.N- | 0.08 ± 0.11abc | C.N+ | 0.13 ± 0.10b |
|  | 14d | 0.06 ± 0.08b |  |  |  |  | 14d.C | 0.08 ± 0.09abc | 14d.N- | 0.04 ± NAabc | D.N+ | 0.15 ± 0.12ab |
|  |  | |  |  |  |  | 0d.D | 0.21 ± 0.11ab | 0d.N+ | 0.22 ± 0.10c |  | |
|  |  |  |  |  |  |  | 2d.D | 0.13 ± 0.11abc | 2d.N+ | 0.12 ± 0.10abc |  |  |
|  |  |  |  |  |  |  | 7d.D | 0.07 ± 0.08c | 7d.N+ | 0.09 ± 0.07ab |  |  |
|  |  |  |  |  |  |  | 14d.D | 0.00 ± 0.00c | 14d.N+ | 0.06 ± 0.09a |  |  |
| Benzaldehyde | 0d | 0.17 ± 0.15a | C | 0.11 ± 0.12a | N- | 0.03 ± 0.05a | 0d.C | 0.18 ± 0.15a | 0d.N- | 0.04 ± 0.05ab | C.N- | 0.04 ± 0.06ab |
|  | 2d | 0.13 ± 0.13a | D | 0.15 ± 0.15a | N+ | 0.15 ± 0.14b | 2d.C | 0.08 ± 0.07a | 2d.N- | 0.00 ± 0.00ab | D.N- | 0.01 ± 0.02a |
|  | 7d | 0.11 ± 0.13a |  | |  | | 7d.C | 0.12 ± 0.13a | 7d.N- | 0.05 ± 0.07abc | C.N+ | 0.13 ± 0.13ab |
|  | 14d | 0.02 ± 0.05a |  |  |  |  | 14d.C | 0.03 ± 0.06a | 14d.N- | 0.00 ± NAabc | D.N+ | 0.17 ± 0.15b |
|  |  | |  |  |  |  | 0d.D | 0.17 ± 0.14a | 0d.N+ | 0.21 ± 0.14ac |  | |
|  |  |  |  |  |  |  | 2d.D | 0.19 ± 0.17a | 2d.N+ | 0.16 ± 0.13bc |  |  |
|  |  |  |  |  |  |  | 7d.D | 0.10 ± 0.13a | 7d.N+ | 0.11 ± 0.13abc |  |  |
|  |  |  |  |  |  |  | 14d.D | 0.00 ± 0.00a | 14d.N+ | 0.03 ± 0.06ab |  |  |
| Benzyl alcohol | 0d | 0.05 ± 0.05a | C | 0.03 ± 0.04a | N- | 0.04 ± 0.08a | 0d.C | 0.06 ± 0.06a | 0d.N- | 0.02 ± 0.03ab | C.N- | 0.02 ± 0.03a |
|  | 2d | 0.04 ± 0.04a | D | 0.05 ± 0.05a | N+ | 0.04 ± 0.04a | 2d.C | 0.03 ± 0.03a | 2d.N- | 0.02 ± 0.03ab | D.N- | 0.06 ± 0.12a |
|  | 7d | 0.04 ± 0.07a |  | |  | | 7d.C | 0.02 ± 0.02a | 7d.N- | 0.14 ± 0.18ab | C.N+ | 0.04 ± 0.04a |
|  | 14d | 0.01 ± 0.02a |  |  |  |  | 14d.C | 0.01 ± 0.01a | 14d.N- | 0.00 ± NAabc | D.N+ | 0.05 ± 0.04a |
|  |  | |  |  |  |  | 0d.D | 0.05 ± 0.03a | 0d.N+ | 0.06 ± 0.05a |  | |
|  |  |  |  |  |  |  | 2d.D | 0.05 ± 0.04a | 2d.N+ | 0.04 ± 0.04ab |  |  |
|  |  |  |  |  |  |  | 7d.D | 0.06 ± 0.09a | 7d.N+ | 0.02 ± 0.03b |  |  |
|  |  |  |  |  |  |  | 14d.D | 0.03 ± 0.04a | 14d.N+ | 0.01 ± 0.02ab |  |  |
| Caryophyllene | 0d | 0.01 ± 0.04a | C | 0.02 ± 0.05a | N- | 0.004 ± 0.01a | 0d.C | 0.02 ± 0.06a | 0d.N- | 0.002 ± 0.005a | C.N- | 0.001 ± 0.002a |
|  | 2d | 0.02 ± 0.06a | D | 0.002 ± 0.01a | N+ | 0.01 ± 0.04a | 2d.C | 0.03 ± 0.08a | 2d.N- | 0.01 ± 0.01a | D.N- | 0.01 ± 0.01a |
|  | 7d | 0.002 ± 0.004a |  | |  | | 7d.C | 0.003 ± 0.01a | 7d.N- | 0.00 ± 0.00a | C.N+ | 0.02 ± 0.06a |
|  | 14d | 0.005 ± 0.01a |  |  |  |  | 14d.C | 0.01 ± 0.01a | 14d.N- | 0.00 ± NAa | D.N+ | 0.001 ± 0.002a |
|  |  | |  |  |  |  | 0d.D | 0.003 ± 0.004a | 0d.N+ | 0.01 ± 0.05a |  | |
|  |  |  |  |  |  |  | 2d.D | 0.003 ± 0.01a | 2d.N+ | 0.02 ± 0.07a |  |  |
|  |  |  |  |  |  |  | 7d.D | 0.00 ± 0.00a | 7d.N+ | 0.002 ± 0.004a |  |  |
|  |  |  |  |  |  |  | 14d.D | 0.00 ± 0.00a | 14d.N+ | 0.01 ± 0.01a |  |  |
| Hexadecanoic acid | 0d | 0.02 ± 0.04a | C | 0.04 ± 0.07a | N- | 0.01 ± 0.03a | 0d.C | 0.02 ± 0.05a | 0d.N- | 0.02 ± 0.05a | C.N- | 0.00 ± 0.00a |
|  | 2d | 0.02 ± 0.05a | D | 0.02 ± 0.05a | N+ | 0.03 ± 0.06a | 2d.C | 0.03 ± 0.05a | 2d.N- | 0.00 ± 0.00a | D.N- | 0.02 ± 0.05a |
|  | 7d | 0.05 ± 0.09a |  | |  | | 7d.C | 0.06 ± 0.11a | 7d.N- | 0.00 ± 0.00a | C.N+ | 0.05 ± 0.08a |
|  | 14d | 0.02 ± 0.07a |  |  |  |  | 14d.C | 0.03 ± 0.08a | 14d.N- | 0.00 ± NAa | D.N+ | 0.02 ± 0.05a |
|  |  | |  |  |  |  | 0d.D | 0.02 ± 0.04a | 0d.N+ | 0.02 ± 0.04a |  | |
|  |  |  |  |  |  |  | 2d.D | 0.01 ± 0.03a | 2d.N+ | 0.03 ± 0.05a |  |  |
|  |  |  |  |  |  |  | 7d.D | 0.04 ± 0.07a | 7d.N+ | 0.06 ± 0.09a |  |  |
|  |  |  |  |  |  |  | 14d.D | 0.00 ± 0.00a | 14d.N+ | 0.03 ± 0.08a |  |  |
| Limonene | 0d | 0.002 ± 0.01a | C | 0.004 ± 0.01a | N- | 0.01 ± 0.02a | 0d.C | 0.002 ± 0.01a | 0d.N- | 0.01 ± 0.01a | C.N- | 0.01 ± 0.02a |
|  | 2d | 0.004 ± 0.01a | D | 0.001 ± 0.003a | N+ | 0.001 ± 0.01a | 2d.C | 0.01 ± 0.02a | 2d.N- | 0.02 ± 0.03a | D.N- | 0.004 ± 0.01a |
|  | 7d | 0.00 ± 0.00a |  | |  | | 7d.C | 0.00 ± 0.00a | 7d.N- | 0.00 ± 0.00a | C.N+ | 0.002 ± 0.01a |
|  | 14d | 0.004 ± 0.01a |  |  |  |  | 14d.C | 0.01 ± 0.01a | 14d.N- | 0.00 ± NAa | D.N+ | 0.00 ± 0.00a |
|  |  | |  |  |  |  | 0d.D | 0.001 ± 0.01a | 0d.N+ | 0.00 ± 0.00a |  | |
|  |  |  |  |  |  |  | 2d.D | 0.00 ± 0.00a | 2d.N+ | 0.002 ± 0.01a |  |  |
|  |  |  |  |  |  |  | 7d.D | 0.00 ± 0.00a | 7d.N+ | 0.00 ± 0.00a |  |  |
|  |  |  |  |  |  |  | 14d.D | 0.00 ± 0.00a | 14d.N+ | 0.005 ± 0.01a |  |  |
| Linalool | Not emitted | | | | | | | | | | | |
| Linolenic acid | 0d | 0.08 ± 0.11a | C | 0.08 ± 0.11a | N- | 0.07 ± 0.09a | 0d.C | 0.07 ± 0.12a | 0d.N- | 0.09 ± 0.08a | C.N- | 0.08 ± 0.09a |
|  | 2d | 0.05 ± 0.10a | D | 0.07 ± 0.11a | N+ | 0.08 ± 0.11a | 2d.C | 0.07 ± 0.12a | 2d.N- | 0.05 ± 0.11a | D.N- | 0.06 ± 0.10a |
|  | 7d | 0.11 ± 0.13a |  | |  | | 7d.C | 0.10 ± 0.12a | 7d.N- | 0.11 ± 0.15a | C.N+ | 0.08 ± 0.12a |
|  | 14d | 0.06 ± 0.10a |  |  |  |  | 14d.C | 0.07 ± 0.12a | 14d.N- | 0.00 ± NAa | D.N+ | 0.07 ± 0.11a |
|  |  | |  |  |  |  | 0d.D | 0.08 ± 0.10a | 0d.N+ | 0.07 ± 0.12a |  | |
|  |  |  |  |  |  |  | 2d.D | 0.03 ± 0.06a | 2d.N+ | 0.05 ± 0.10a |  |  |
|  |  |  |  |  |  |  | 7d.D | 0.12 ± 0.15a | 7d.N+ | 0.11 ± 0.13a |  |  |
|  |  |  |  |  |  |  | 14d.D | 0.03 ± 0.04a | 14d.N+ | 0.07 ± 0.11a |  |  |
| Methyl salicylate | 0d | 0.02 ± 0.02a | C | 0.03 ± 0.04a | N- | 0.05 ± 0.06a | 0d.C | 0.02 ± 0.02a | 0d.N- | 0.04 ± 0.02a | C.N- | 0.06 ± 0.07a |
|  | 2d | 0.04 ± 0.06a | D | 0.02 ± 0.04a | N+ | 0.02 ± 0.04a | 2d.C | 0.06 ± 0.07a | 2d.N- | 0.08 ± 0.10a | D.N- | 0.03 ± 0.03a |
|  | 7d | 0.03 ± 0.05a |  | |  | | 7d.C | 0.02 ± 0.04a | 7d.N- | 0.01 ± 0.02a | C.N+ | 0.03 ± 0.04a |
|  | 14d | 0.02 ± 0.02a |  |  |  |  | 14d.C | 0.02 ± 0.02a | 14d.N- | 0.07 ± NAa | D.N+ | 0.02 ± 0.04a |
|  |  | |  |  |  |  | 0d.D | 0.02 ± 0.02a | 0d.N+ | 0.02 ± 0.01a |  | |
|  |  |  |  |  |  |  | 2d.D | 0.02 ± 0.04a | 2d.N+ | 0.03 ± 0.05a |  |  |
|  |  |  |  |  |  |  | 7d.D | 0.03 ± 0.05a | 7d.N+ | 0.03 ± 0.05a |  |  |
|  |  |  |  |  |  |  | 14d.D | 0.02 ± 0.03a | 14d.N+ | 0.02 ± 0.02a |  |  |
| Myrcene | 0d | 0.01 ± 0.01a | C | 0.01 ± 0.02a | N- | 0.03 ± 0.04a | 0d.C | 0.004 ± 0.01a | 0d.N- | 0.02 ± 0.01ab | C.N- | 0.02 ± 0.03ab |
|  | 2d | 0.01 ± 0.02a | D | 0.02 ± 0.03a | N+ | 0.01 ± 0.02a | 2d.C | 0.02 ± 0.03a | 2d.N- | 0.04 ± 0.04b | D.N- | 0.05 ± 0.04a |
|  | 7d | 0.02 ± 0.03a |  | |  | | 7d.C | 0.01 ± 0.01a | 7d.N- | 0.06 ± 0.08ab | C.N+ | 0.01 ± 0.01ab |
|  | 14d | 0.02 ± 0.03a |  |  |  |  | 14d.C | 0.02 ± 0.02a | 14d.N- | 0.00 ± NAab | D.N+ | 0.01 ± 0.02b |
|  |  | |  |  |  |  | 0d.D | 0.01 ± 0.01a | 0d.N+ | 0.003 ± 0.01a |  | |
|  |  |  |  |  |  |  | 2d.D | 0.01 ± 0.02a | 2d.N+ | 0.01 ± 0.01ab |  |  |
|  |  |  |  |  |  |  | 7d.D | 0.02 ± 0.04a | 7d.N+ | 0.01 ± 0.02ab |  |  |
|  |  |  |  |  |  |  | 14d.D | 0.04 ± 0.05a | 14d.N+ | 0.03 ± 0.03b |  |  |
| (*E*3,*E*7)-4,8,12-Trimethyltrideca-1,3,7,11-tetraene | 0d | 0.01 ± 0.02a | C | 0.03 ± 0.04a | N- | 0.04 ± 0.04a | 0d.C | 0.01 ± 0.01a | 0d.N- | 0.03 ± 0.03a | C.N- | 0.04 ± 0.04a |
|  | 2d | 0.02 ± 0.04a | D | 0.01 ± 0.02a | N+ | 0.02 ± 0.03a | 2d.C | 0.04 ± 0.05a | 2d.N- | 0.06 ± 0.06a | D.N- | 0.03 ± 0.03ab |
|  | 7d | 0.02 ± 0.03a |  | |  | | 7d.C | 0.03 ± 0.04a | 7d.N- | 0.01 ± 0.02a | C.N+ | 0.03 ± 0.04a |
|  | 14d | 0.03 ± 0.01a |  |  |  |  | 14d.C | 0.03 ± 0.01a | 14d.N- | 0.03 ± NAa | D.N+ | 0.005 ± 0.01b |
|  |  | |  |  |  |  | 0d.D | 0.02 ± 0.02a | 0d.N+ | 0.01 ± 0.01a |  | |
|  |  |  |  |  |  |  | 2d.D | 0.004 ± 0.01a | 2d.N+ | 0.02 ± 0.04a |  |  |
|  |  |  |  |  |  |  | 7d.D | 0.003 ± 0.01a | 7d.N+ | 0.02 ± 0.03a |  |  |
|  |  |  |  |  |  |  | 14d.D | 0.01 ± 0.02a | 14d.N+ | 0.03 ± 0.01a |  |  |
| Verbenone | Not emitted | | | | | | | | | | | |

Lowercase letters denote significant differences within factors.

**Table S9** Relative amount (mean ± SD) of volatile compounds of *Sinapis arvensis* in the different timepoints (0d, 2d, 7d, 14d), treatments and their interactions. “C” = Watered treatment, “D” = Drought-stressed treatment, “N-“ = without additional nitrogen, “N+” = with additional nitrogen. Significance was assessed using Analysis of Deviance Table (Type II tests).

| **Compound** | **Factors** | | | | | | | | | | | |
| --- | --- | --- | --- | --- | --- | --- | --- | --- | --- | --- | --- | --- |
|  | Time | | Watering | | Nitrogen | | Time × Watering | | Time × Nitrogen | | Watering × Nitrogen | |
| (3*E*)-4,8-dimethyl-1,3,7-Nonatriene | 0d | 0.01 ± 0.01a | C | 0.01 ± 0.02a | N- | 0.01 ± 0.01a | 0d.C | 0.02 ± 0.02a | 0d.N- | 0.01 ± 0.01a | C.N- | 0.01 ± 0.01a |
|  | 2d | 0.01 ± 0.01a | D | 0.01 ± 0.01a | N+ | 0.02 ± 0.02a | 2d.C | 0.01 ± 0.01a | 2d.N- | 0.01 ± 0.01a | D.N- | 0.01 ± 0.01a |
|  | 7d | 0.01 ± 0.02a |  | |  | | 7d.C | 0.02 ± 0.02a | 7d.N- | 0.01 ± 0.01a | C.N+ | 0.02 ± 0.02a |
|  | 14d | 0.01 ± 0.01a |  |  |  |  | 14d.C | 0.02 ± 0.01a | 14d.N- | 0.01 ± 0.01a | D.N+ | 0.01 ± 0.02a |
|  |  | |  |  |  |  | 0d.D | 0.01 ± 0.01a | 0d.N+ | 0.02 ± 0.02a |  | |
|  |  |  |  |  |  |  | 2d.D | 0.01 ± 0.02a | 2d.N+ | 0.01 ± 0.02a |  |  |
|  |  |  |  |  |  |  | 7d.D | 0.01 ± 0.02a | 7d.N+ | 0.02 ± 0.02a |  |  |
|  |  |  |  |  |  |  | 14d.D | 0.002 ± 0.004a | 14d.N+ | 0.01 ± 0.01a |  |  |
| (*3Z*)-3-Hexenyl acetate | 0d | 0.03 ± 0.06a | C | 0.03 ± 0.04a | N- | 0.02 ± 0.6a | 0d.C | 0.01 ± 0.03a | 0d.N- | 0.03 ± 0.05a | C.N- | 0.02 ± 0.04a |
|  | 2d | 0.05 ± 0.08a | D | 0.05 ± 0.08a | N+ | 0.04 ±0.06b | 2d.C | 0.02 ± 0.05a | 2d.N- | 0.04 ± 0.08a | D.N- | 0.04 ± 0.07a |
|  | 7d | 0.03 ± 0.03a |  | |  | | 7d.C | 0.02 ± 0.02a | 7d.N- | 0.01 ± 0.02a | C.N+ | 0.03 ± 0.05a |
|  | 14d | 0.04 ± 0.05a |  |  |  |  | 14d.C | 0.05 ± 0.05a | 14d.N- | 0.03 ± 0.06 | D.N+ | 0.06 ± 0.08a |
|  |  | |  |  |  |  | 0d.D | 0.05 ± 0.09a | 0d.N+ | 0.04 ± 0.07a |  | |
|  |  |  |  |  |  |  | 2d.D | 0.08 ± 0.10a | 2d.N+ | 0.06 ± 0.08a |  |  |
|  |  |  |  |  |  |  | 7d.D | 0.03 ± 0.04a | 7d.N+ | 0.04 ± 0.04a |  |  |
|  |  |  |  |  |  |  | 14d.D | 0.02 ± 0.04a | 14d.N+ | 0.04 ± 0.04a |  |  |
| (*E,E*)-α-Farnesene | 0d | 0.002 ± 0.004a | C | 0.003 ± 0.005a | N- | 0.003 ± 0.005a | 0d.C | 0.003 ± 0.005a | 0d.N- | 0.003 ± 0.005a | C.N- | 0.004 ± 0.005a |
|  | 2d | 0.002 ± 0.004a | D | 0.001 ± 0.003b | N+ | 0.002 ± 0.004a | 2d.C | 0.002 ± 0.004a | 2d.N- | 0.002 ± 0.005a | D.N- | 0.002 ± 0.004a |
|  | 7d | 0.003 ± 0.01a |  | |  | | 7d.C | 0.004 ± 0.006a | 7d.N- | 0.002 ± 0.005a | C.N+ | 0.002 ± 0.004a |
|  | 14d | 0.002 ± 0.004a |  |  |  |  | 14d.C | 0.004 ± 0.004a | 14d.N- | 0.003 ± 0.005a | D.N+ | 0.001 ± 0.003a |
|  |  | |  |  |  |  | 0d.D | 0.002 ± 0.003a | 0d.N+ | 0.002 ± 0.003a |  | |
|  |  |  |  |  |  |  | 2d.D | 0.002 ± 0.004a | 2d.N+ | 0.001 ± 0.003a |  |  |
|  |  |  |  |  |  |  | 7d.D | 0.002 ± 0.005a | 7d.N+ | 0.003 ± 0.01a |  |  |
|  |  |  |  |  |  |  | 14d.D | 0.00 ± 0.00a | 14d.N+ | 0.001 ± 0.003a |  |  |
| (*E*)-β-Farnesene | 0d | 0.004 ± 0.01a | C | 0.01 ± 0.02a | N- | 0.003 ± 0.01a | 0d.C | 0.01 ± 0.01a | 0d.N- | 0.004 ± 0.01a | C.N- | 0.01 ± 0.01a |
|  | 2d | 0.01 ± 0.02a | D | 0.001 ± 0.004a | N+ | 0.005 ± 0.02a | 2d.C | 0.01 ± 0.03a | 2d.N- | 0.003 ± 0.01a | D.N- | 0.001 ± 0.004a |
|  | 7d | 0.0003 ± 0.001a |  | |  | | 7d.C | 0.00 ± 0.00a | 7d.N- | 0.001 ± 0.002a | C.N+ | 0.01 ± 0.02a |
|  | 14d | 0.003 ± 0.01a |  |  |  |  | 14d.C | 0.004 ± 0.01a | 14d.N- | 0.004 ± 0.01a | D.N+ | 0.001 ± 0.004a |
|  |  | |  |  |  |  | 0d.D | 0.002 ± 0.01a | 0d.N+ | 0.004 ± 0.01a |  | |
|  |  |  |  |  |  |  | 2d.D | 0.0002 ± 0.001a | 2d.N+ | 0.01 ± 0.03a |  |  |
|  |  |  |  |  |  |  | 7d.D | 0.0005 ± 0.001a | 7d.N+ | 0.00 ± 0.00a |  |  |
|  |  |  |  |  |  |  | 14d.D | 0.002 ± 0.01a | 14d.N+ | 0.003 ± 0.01a |  |  |
| (*E*)-β-Ocimene | 0d | 0.24 ± 0.10a | C | 0.25 ± 0.10a | N- | 0.23 ± 0.13a | 0d.C | 0.26 ± 0.08a | 0d.N- | 0.24 ± 0.13a | C.N- | 0.29 ± 0.11a |
|  | 2d | 0.22 ± 0.11a | D | 0.19 ± 0.11b | N+ | 0.22 ± 0.10a | 2d.C | 0.23 ± 0.12abc | 2d.N- | 0.23 ± 0.13a | D.N- | 0.16 ± 0.11b |
|  | 7d | 0.24 ± 0.09a |  | |  | | 7d.C | 0.27 ± 0.08abc | 7d.N- | 0.23 ± 0.11a | C.N+ | 0.23 ± 0.09ab |
|  | 14d | 0.20 ± 0.14a |  |  |  |  | 14d.C | 0.28 ± 0.12ab | 14d.N- | 0.20 ± 0.15a | D.N+ | 0.22 ± 0.11ab |
|  |  | |  |  |  |  | 0d.D | 0.21 ± 0.11abc | 0d.N+ | 0.24 ± 0.07a |  | |
|  |  |  |  |  |  |  | 2d.D | 0.20 ± 0.10abc | 2d.N+ | 0.20 ± 0.10a |  |  |
|  |  |  |  |  |  |  | 7d.D | 0.22 ± 0.10abc | 7d.N+ | 0.25 ± 0.09a |  |  |
|  |  |  |  |  |  |  | 14d.D | 0.12 ± 0.11c | 14d.N+ | 0.20 ± 0.14a |  |  |
| (*Z*)-β-Ocimene | 0d | 0.02 ± 0.02a | C | 0.03 ± 0.02a | N- | 0.02 ± 0.02a | 0d.C | 0.03± 0.02a | 0d.N- | 0.03 ± 0.02a | C.N- | 0.03 ± 0.02a |
|  | 2d | 0.02 ± 0.02a | D | 0.01 ± 0.02b | N+ | 0.02 ± 0.02a | 2d.C | 0.02 ± 0.02a | 2d.N- | 0.02 ± 0.02a | D.N- | 0.01 ± 0.02b |
|  | 7d | 0.02 ± 0.02a |  | |  | | 7d.C | 0.03 ± 0.02a | 7d.N- | 0.02 ± 0.02a | C.N+ | 0.03 ± 0.02ab |
|  | 14d | 0.02 ± 0.02a |  |  |  |  | 14d.C | 0.04 ± 0.02a | 14d.N- | 0.02 ± 0.03a | D.N+ | 0.02 ± 0.02b |
|  |  | |  |  |  |  | 0d.D | 0.02 ± 0.02a | 0d.N+ | 0.02 ± 0.02a |  | |
|  |  |  |  |  |  |  | 2d.D | 0.02 ± 0.02a | 2d.N+ | 0.02 ± 0.02a |  |  |
|  |  |  |  |  |  |  | 7d.D | 0.01 ± 0.02a | 7d.N+ | 0.02 ± 0.02a |  |  |
|  |  |  |  |  |  |  | 14d.D | 0.01 ± 0.01a | 14d.N+ | 0.03 ± 0.02a |  |  |
| 6-Methyl-5-heptene-2-one | 0d | 0.002 ± 0.01a | C | 0.004 ± 0.01a | N- | 0.003 ± 0.01a | 0d.C | 0.002 ± 0.01a | 0d.N- | 0.003 ± 0.01a | C.N- | 0.003 ± 0.01a |
|  | 2d | 0.003 ± 0.01a | D | 0.004 ± 0.01a | N+ | 0.005 ± 0.01 a | 2d.C | 0.004 ± 0.01a | 2d.N- | 0.003 ± 0.01a | D.N- | 0.002 ± 0.01a |
|  | 7d | 0.01 ± 0.02a |  | |  | | 7d.C | 0.003 ± 0.01a | 7d.N- | 0.00 ± 0.00a | C.N+ | 0.005 ± 0.01a |
|  | 14d | 0.005 ± 0.01a |  |  |  |  | 14d.C | 0.01 ± 0.01a | 14d.N- | 0.003 ± 0.005a | D.N+ | 0.005 ± 0.02a |
|  |  | |  |  |  |  | 0d.D | 0.002 ± 0.004a | 0d.N+ | 0.001 ± 0.003a |  | |
|  |  |  |  |  |  |  | 2d.D | 0.003 ± 0.01a | 2d.N+ | 0.004 ± 0.01a |  |  |
|  |  |  |  |  |  |  | 7d.D | 0.01 ± 0.03a | 7d.N+ | 0.01 ± 0.03a |  |  |
|  |  |  |  |  |  |  | 14d.D | 0.00 ± 0.00a | 14d.N+ | 0.01 ± 0.01a |  |  |
| 2-Undecanone | 0d | 0.00 ± 0.00a | C | 0.0004 ± 0.002a | N- | 0.00 ± 0.00a | 0d.C | 0.00 ± 0.00a | 0d.N- | 0.00 ± 0.00a | C.N- | 0.00 ± 0.00a |
|  | 2d | 0.001 ± 0.003a | D | 0.02 ± 0.01a | N+ | 0.0005 ± 0.003a | 2d.C | 0.001 ± 0.004a | 2d.N- | 0.00 ± 0.00a | D.N- | 0.00 ± 0.00a |
|  | 7d | 0.00 ± 0.00a |  | |  | | 7d.C | 0.00 ± 0.00a | 7d.N- | 0.00 ± 0.00a | C.N+ | 0.001 ± 0.003a |
|  | 14d | 0.001 ± 0.002a |  |  |  |  | 14d.C | 0.00 ± 0.00a | 14d.N- | 0.00 ± 0.00a | D.N+ | 0.0004 ± 0.002a |
|  |  | |  |  |  |  | 0d.D | 0.00 ± 0.00a | 0d.N+ | 0.00 ± 0.00a |  | |
|  |  |  |  |  |  |  | 2d.D | 0.00 ± 0.00a | 2d.N+ | 0.001 ± 0.004a |  |  |
|  |  |  |  |  |  |  | 7d.D | 0.00 ± 0.00a | 7d.N+ | 0.00 ± 0.00a |  |  |
|  |  |  |  |  |  |  | 14d.D | 0.001 ± 0.003a | 14d.N+ | 0.001 ± 0.003a |  |  |
| α-Longipinene | 0d | 0.04 ± 0.05a | C | 0.03 ± 0.05a | N- | 0.03 ± 0.03a | 0d.C | 0.03 ± 0.05a | 0d.N- | 0.04 ± 0.04a | C.N- | 0.02 ± 0.03a |
|  | 2d | 0.04 ± 0.05a | D | 0.04 ± 0.05a | N+ | 0.04 ± 0.05a | 2d.C | 0.03 ± 0.06a | 2d.N- | 0.02 ± 0.03a | D.N- | 0.03 ± 0.04a |
|  | 7d | 0.02 ± 0.03a |  | |  | | 7d.C | 0.01 ± 0.02a | 7d.N- | 0.02 ± 0.02a | C.N+ | 0.03 ± 0.05a |
|  | 14d | 0.02 ± 0.04a |  |  |  |  | 14d.C | 0.02 ± 0.04a | 14d.N- | 0.02 ± 0.03a | D.N+ | 0.05 ± 0.05a |
|  |  | |  |  |  |  | 0d.D | 0.05 ± 0.05a | 0d.N+ | 0.04 ± 0.06a |  | |
|  |  |  |  |  |  |  | 2d.D | 0.04 ± 0.05a | 2d.N+ | 0.05 ± 0.06a |  |  |
|  |  |  |  |  |  |  | 7d.D | 0.03 ± 0.04a | 7d.N+ | 0.02 ± 0.04a |  |  |
|  |  |  |  |  |  |  | 14d.D | 0.03 ± 0.04a | 14d.N+ | 0.03 ± 0.04a |  |  |
| α-Pinene | 0d | 0.01 ± 0.02a | C | 0.02 ± 0.07a | N- | 0.03 ± 0.08a | 0d.C | 0.01 ± 0.02a | 0d.N- | 0.01 ± 0.03a | C.N- | 0.03 ± 0.11a |
|  | 2d | 0.03 ± 0.09a | D | 0.03 ± 0.11a | N+ | 0.02 ± 0.10a | 2d.C | 0.04 ± 0.11a | 2d.N- | 0.06 ± 0.14a | D.N- | 0.02 ± 0.03a |
|  | 7d | 0.02 ± 0.03a |  | |  | | 7d.C | 0.005 ± 0.01a | 7d.N- | 0.01 ± 0.01a | C.N+ | 0.01 ± 0.01a |
|  | 14d | 0.05 ± 0.18a |  |  |  |  | 14d.C | 0.00 ± 0.00a | 14d.N- | 0.01 ± 0.01a | D.N+ | 0.05 ± 0.15a |
|  |  | |  |  |  |  | 0d.D | 0.01 ± 0.02a | 0d.N+ | 0.01 ± 0.01a |  | |
|  |  |  |  |  |  |  | 2d.D | 0.02 ± 0.04a | 2d.N+ | 0.01 ± 0.03a |  |  |
|  |  |  |  |  |  |  | 7d.D | 0.03 ± 0.04a | 7d.N+ | 0.02 ± 0.04a |  |  |
|  |  |  |  |  |  |  | 14d.D | 0.11 ± 0.26a | 14d.N+ | 0.08 ± 0.24a |  |  |
| Anisaldehyde | Not emitted | | | | | | | | | | | |
| Benzaldehyde | 0d | 0.02 ± 0.04a | C | 0.02 ± 0.04a | N- | 0.02 ± 0.03a | 0d.C | 0.03 ± 0.05a | 0d.N- | 0.01 ± 0.02a | C.N- | 0.02 ± 0.04a |
|  | 2d | 0.02 ± 0.04a | D | 0.03 ± 0.06a | N+ | 0.03 ± 0.06a | 2d.C | 0.02 ± 0.03a | 2d.N- | 0.03 ± 0.03a | D.N- | 0.01 ± 0.03a |
|  | 7d | 0.05 ± 0.08a |  | |  | | 7d.C | 0.04 ± 0.06a | 7d.N- | 0.02 ± 0.04a | C.N+ | 0.02 ± 0.04a |
|  | 14d | 0.03 ± 0.05a |  |  |  |  | 14d.C | 0.02 ± 0.02a | 14d.N- | 0.02 ± 0.04a | D.N+ | 0.05 ± 0.07a |
|  |  | |  |  |  |  | 0d.D | 0.01 ± 0.03a | 0d.N+ | 0.03 ± 0.05a |  | |
|  |  |  |  |  |  |  | 2d.D | 0.03 ± 0.04a | 2d.N+ | 0.02 ± 0.04a |  |  |
|  |  |  |  |  |  |  | 7d.D | 0.05 ± 0.10a | 7d.N+ | 0.07 ± 0.09a |  |  |
|  |  |  |  |  |  |  | 14d.D | 0.04 ± 0.06a | 14d.N+ | 0.04 ± 0.05a |  |  |
| Benzyl alcohol | 0d | 0.01 ± 0.01a | C | 0.01 ± 0.01a | N- | 0.01 ± 0.01a | 0d.C | 0.003 ± 0.004a | 0d.N- | 0.001 ± 0.003a | C.N- | 0.01 ± 0.01a |
|  | 2d | 0.01 ± 0.02a | D | 0.01 ± 0.02a | N+ | 0.01 ± 0.01a | 2d.C | 0.01 ± 0.02a | 2d.N- | 0.01 ± 0.02a | D.N- | 0.01 ± 0.02a |
|  | 7d | 0.01 ± 0.01a |  | |  | | 7d.C | 0.01 ± 0.01a | 7d.N- | 0.005 ± 0.01a | C.N+ | 0.01 ± 0.01a |
|  | 14d | 0.01 ± 0.02a |  |  |  |  | 14d.C | 0.01 ± 0.01a | 14d.N- | 0.02 ± 0.02a | D.N+ | 0.01 ± 0.02a |
|  |  | |  |  |  |  | 0d.D | 0.01 ± 0.02a | 0d.N+ | 0.01 ± 0.01a |  | |
|  |  |  |  |  |  |  | 2d.D | 0.01 ± 0.02a | 2d.N+ | 0.01 ± 0.02a |  |  |
|  |  |  |  |  |  |  | 7d.D | 0.01 ± 0.01a | 7d.N+ | 0.01 ± 0.01a |  |  |
|  |  |  |  |  |  |  | 14d.D | 0.02 ± 0.02a | 14d.N+ | 0.01 ± 0.02a |  |  |
| Caryophyllene | 0d | 0.04 ± 0.05a | C | 0.04 ± 0.05a | N- | 0.04 ± 0.04a | 0d.C | 0.04 ± 0.05a | 0d.N- | 0.04 ± 0.05a | C.N- | 0.04 ± 0.04a |
|  | 2d | 0.04 ± 0.04a | D | 0.04 ± 0.05a | N+ | 0.04 ± 0.05a | 2d.C | 0.04 ± 0.04a | 2d.N- | 0.04 ± 0.04a | D.N- | 0.04 ± 0.05a |
|  | 7d | 0.03 ± 0.04a |  | |  | | 7d.C | 0.05 ± 0.04a | 7d.N- | 0.02 ± 0.03a | C.N+ | 0.05 ± 0.05a |
|  | 14d | 0.05 ± 0.06a |  |  |  |  | 14d.C | 0.06 ± 0.06a | 14d.N- | 0.05 ± 0.06a | D.N+ | 0.03 ± 0.05a |
|  |  | |  |  |  |  | 0d.D | 0.05 ± 0.05a | 0d.N+ | 0.04 ± 0.06a |  | |
|  |  |  |  |  |  |  | 2d.D | 0.04 ± 0.05a | 2d.N+ | 0.04 ± 0.05a |  |  |
|  |  |  |  |  |  |  | 7d.D | 0.02 ± 0.03a | 7d.N+ | 0.04 ± 0.05a |  |  |
|  |  |  |  |  |  |  | 14d.D | 0.03 ± 0.06a | 14d.N+ | 0.05 ± 0.06a |  |  |
| Hexadecanoic acid | 0d | 0.03 ± 0.06a | C | 0.04 ± 0.05a | N- | 0.05 ± 0.08a | 0d.C | 0.03 ± 0.05a | 0d.N- | 0.02 ± 0.04a | C.N- | 0.02 ± 0.04a |
|  | 2d | 0.04 ± 0.07a | D | 0.07 ± 0.09a | N+ | 0.05 ± 0.06a | 2d.C | 0.03 ± 0.06a | 2d.N- | 0.02 ± 0.07a | D.N- | 0.09 ± 0.10b |
|  | 7d | 0.09 ± 0.07b |  | |  | | 7d.C | 0.06 ± 0.04ab | 7d.N- | 0.13 ± 0.07b | C.N+ | 0.05 ± 0.06ab |
|  | 14d | 0.06 ± 0.09ab |  |  |  |  | 14d.C | 0.04 ± 0.06ab | 14d.N- | 0.10 ± 0.12ab | D.N+ | 0.05 ± 0.07ab |
|  |  | |  |  |  |  | 0d.D | 0.04 ± 0.07ab | 0d.N+ | 0.04 ± 0.07ab |  | |
|  |  |  |  |  |  |  | 2d.D | 0.05 ± 0.09ab | 2d.N+ | 0.05 ± 0.08ab |  |  |
|  |  |  |  |  |  |  | 7d.D | 0.11 ± 0.08b | 7d.N+ | 0.05 ± 0.05ab |  |  |
|  |  |  |  |  |  |  | 14d.D | 0.09 ± 0.12ab | 14d.N+ | 0.04 ± 0.07ab |  |  |
| Limonene | 0d | 0.06 ± 0.04a | C | 0.04 ± 0.04a | N- | 0.05 ± 0.04a | 0d.C | 0.05 ± 0.04a | 0d.N- | 0.06 ± 0.04a | C.N- | 0.04 ± 0.03a |
|  | 2d | 0.06 ± 0.04a | D | 0.06 ± 0.04b | N+ | 0.05 ± 0.04a | 2d.C | 0.06 ± 0.04a | 2d.N- | 0.06 ± 0.04a | D.N- | 0.06 ± 0.05a |
|  | 7d | 0.04 ± 0.03a |  | |  | | 7d.C | 0.03 ± 0.03a | 7d.N- | 0.04 ± 0.03a | C.N+ | 0.04 ± 0.04a |
|  | 14d | 0.03 ± 0.04a |  |  |  |  | 14d.C | 0.02 ± 0.02a | 14d.N- | 0.04 ± 0.05a | D.N+ | 0.05 ± 0.03a |
|  |  | |  |  |  |  | 0d.D | 0.06 ± 0.04a | 0d.N+ | 0.05 ± 0.04a |  | |
|  |  |  |  |  |  |  | 2d.D | 0.05 ± 0.03a | 2d.N+ | 0.05 ± 0.03 |  |  |
|  |  |  |  |  |  |  | 7d.D | 0.05 ± 0.04a | 7d.N+ | 0.04 ± 0.04a |  |  |
|  |  |  |  |  |  |  | 14d.D | 0.05 ± 0.05a | 14d.N+ | 0.03 ± 0.03a |  |  |
| Linalool | 0d | 0.0002 ± 0.001a | C | 0.00 ± 0.00a | N- | 0.00 ± 0.00a | 0d.C | 0.00 ± 0.00a | 0d.N- | 0.00 ± 0.00a | C.N- | 0.00 ± 0.00a |
|  | 2d | 0.0002 ± 0.001a | D | 0.0003 ± 0.001a | N+ | 0.0003 ± 0.001a | 2d.C | 0.00 ± 0.00a | 2d.N- | 0.00 ± 0.00a | D.N- | 0.00 ± 0.00a |
|  | 7d | 0.00 ± 0.00a |  | |  | | 7d.C | 0.00 ± 0.00a | 7d.N- | 0.00 ± 0.00a | C.N+ | 0.00 ± 0.00a |
|  | 14d | 0.00 ± 0.00a |  |  |  |  | 14d.C | 0.00 ± 0.00a | 14d.N- | 0.00 ± 0.00a | D.N+ | 0.001 ± 0.002a |
|  |  | |  |  |  |  | 0d.D | 0.001 ± 0.002a | 0d.N+ | 0.0004 ± 0.002a |  | |
|  |  |  |  |  |  |  | 2d.D | 0.001 ± 0.002a | 2d.N+ | 0.0004 ± 0.002a |  |  |
|  |  |  |  |  |  |  | 7d.D | 0.00 ± 0.00a | 7d.N+ | 0.00 ± 0.00a |  |  |
|  |  |  |  |  |  |  | 14d.D | 0.00 ± 0.00a | 14d.N+ | 0.00 ± 0.00a |  |  |
| Linolenic acid | 0d | 0.03 ± 0.06a | C | 0.03 ± 0.06a | N- | 0.05 ± 0.10a | 0d.C | 0.03 ± 0.06a | 0d.N- | 0.004 ± 0.01a | C.N- | 0.01 ± 0.03ab |
|  | 2d | 0.04 ± 0.09a | D | 0.06 ± 0.11a | N+ | 0.04 ± 0.08a | 2d.C | 0.03 ± 0.07a | 2d.N- | 0.02 ± 0.07ab | D.N- | 0.09 ± 0.12b |
|  | 7d | 0.06 ± 0.09a |  | |  | | 7d.C | 0.03 ± 0.04a | 7d.N- | 0.13 ± 0.11b | C.N+ | 0.05 ± 0.07ab |
|  | 14d | 0.06 ± 0.11a |  |  |  |  | 14d.C | 0.05 ± 0.08a | 14d.N- | 0.11 ± 0.15ab | D.N+ | 0.03 ± 0.09a |
|  |  | |  |  |  |  | 0d.D | 0.02 ± 0.07a | 0d.N+ | 0.04 ± 0.08ab |  | |
|  |  |  |  |  |  |  | 2d.D | 0.06 ± 0.12a | 2d.N+ | 0.06 ± 0.11ab |  |  |
|  |  |  |  |  |  |  | 7d.D | 0.09 ± 0.12a | 7d.N+ | 0.01 ± 0.02ab |  |  |
|  |  |  |  |  |  |  | 14d.D | 0.09 ± 0.14a | 14d.N+ | 0.04 ± 0.08ab |  |  |
| Methyl salicylate | 0d | 0.002 ± 0.01a | C | 0.002 ± 0.01a | N- | 0.002 ± 0.01a | 0d.C | 0.002 ± 0.01a | 0d.N- | 0.003 ± 0.01a | C.N- | 0.004 ± 0.01a |
|  | 2d | 0.001 ± 0.01a | D | 0.0003 ± 0.002b | N+ | 0.0004 ± 0.002b | 2d.C | 0.002 ± 0.01a | 2d.N- | 0.002 ± 0.01a | D.N- | 0.001 ± 0.003ab |
|  | 7d | 0.00 ± 0.00a |  | |  | | 7d.C | 0.00 ± 0.00a | 7d.N- | 0.00 ± 0.00a | C.N+ | 0.001 ± 0.003ab |
|  | 14d | 0.002 ± 0.005a |  |  |  |  | 14d.C | 0.003 ± 0.01a | 14d.N- | 0.004 ± 0.01a | D.N+ | 0.00 ± 0.00b |
|  |  | |  |  |  |  | 0d.D | 0.001 ± 0.004a | 0d.N+ | 0.001 ± 0.003a |  | |
|  |  |  |  |  |  |  | 2d.D | 0.00 ± 0.00a | 2d.N+ | 0.001 ± 0.002a |  |  |
|  |  |  |  |  |  |  | 7d.D | 0.00 ± 0.00a | 7d.N+ | 0.00 ± 0.00a |  |  |
|  |  |  |  |  |  |  | 14d.D | 0.00 ± 0.00a | 14d.N+ | 0.00 ± 0.00a |  |  |
| Myrcene | 0d | 0.07 ± 0.03a | C | 0.05 ± 0.04a | N- | 0.06 ± 0.04a | 0d.C | 0.06 ± 0.03a | 0d.N- | 0.07 ± 0.03a | C.N- | 0.05 ± 0.04a |
|  | 2d | 0.06 ± 0.04ab | D | 0.06 ± 0.04a | N+ | 0.05 ± 0.04a | 2d.C | 0.07 ± 0.04a | 2d.N- | 0.06 ± 0.04a | D.N- | 0.06 ± 0.04a |
|  | 7d | 0.04 ± 0.03b |  | |  | | 7d.C | 0.04 ± 0.03a | 7d.N- | 0.04 ± 0.03a | C.N+ | 0.05 ± 0.04a |
|  | 14d | 0.04 ± 0.04a |  |  |  |  | 14d.C | 0.03 ± 0.02a | 14d.N- | 0.05 ± 0.06a | D.N+ | 0.05 ± 0.03a |
|  |  | |  |  |  |  | 0d.D | 0.07 ± 0.03a | 0d.N+ | 0.06 ± 0.03a |  | |
|  |  |  |  |  |  |  | 2d.D | 0.06 ± 0.03a | 2d.N+ | 0.06 ± 0.04a |  |  |
|  |  |  |  |  |  |  | 7d.D | 0.04 ± 0.03a | 7d.N+ | 0.04 ± 0.03a |  |  |
|  |  |  |  |  |  |  | 14d.D | 0.06 ± 0.05a | 14d.N+ | 0.03 ± 0.03a |  |  |
| (*E*3,*E*7)-4,8,12-Trimethyltrideca-1,3,7,11-tetraene | 0d | 0.002 ± 0.005a | C | 0.002 ± 0.004a | N- | 0.002 ± 0.01a | 0d.C | 0.002 ± 0.005a | 0d.N- | 0.003 ± 0.01a | C.N- | 0.002 ± 0.01a |
|  | 2d | 0.001 ± 0.004a | D | 0.001 ± 0.003a | N+ | 0.001 ± 0.002a | 2d.C | 0.002 ± 0.005a | 2d.N- | 0.002 ± 0.01a | D.N- | 0.001 ± 0.004a |
|  | 7d | 0.0003 ± 0.001a |  | |  | | 7d.C | 0.001 ± 0.002a | 7d.N- | 0.00 ± 0.00a | C.N+ | 0.001 ± 0.002a |
|  | 14d | 0.0003 ± 0.001a |  |  |  |  | 14d.C | 0.001 ± 0.001a | 14d.N- | 0.00 ± 0.00a | D.N+ | 0.0005 ± 0.002a |
|  |  | |  |  |  |  | 0d.D | 0.002 ± 0.01a | 0d.N+ | 0.002 ± 0.003a |  | |
|  |  |  |  |  |  |  | 2d.D | 0.00 ± 0.00a | 2d.N+ | 0.001 ± 0.002a |  |  |
|  |  |  |  |  |  |  | 7d.D | 0.00 ± 0.00a | 7d.N+ | 0.001 ± 0.002a |  |  |
|  |  |  |  |  |  |  | 14d.D | 0.00 ± 0.00a | 14d.N+ | 0.0005 ± 0.001a |  |  |
| Verbenone | 0d | 0.12 ± 0.05a | C | 0.10 ± 0.05a | N- | 0.10 ± 0.06a | 0d.C | 0.11 ± 0.04a | 0d.N- | 0.13 ± 0.05a | C.N- | 0.09 ± 0.06a |
|  | 2d | 0.09 ± 0.06a | D | 0.09 ± 0.06a | N+ | 0.09 ± 0.05a | 2d.C | 0.08 ± 0.06a | 2d.N- | 0.09 ± 0.07a | D.N- | 0.11 ± 0.07a |
|  | 7d | 0.09 ± 0.04a |  | |  | | 7d.C | 0.11 ± 0.05a | 7d.N- | 0.07 ± 0.04a | C.N+ | 0.10 ± 0.05a |
|  | 14d | 0.09 ± 0.05a |  |  |  |  | 14d.C | 0.10 ± 0.05a | 14d.N- | 0.09 ± 0.07a | D.N+ | 0.08 ± 0.04a |
|  |  | |  |  |  |  | 0d.D | 0.13 ± 0.05a | 0d.N+ | 0.11 ± 0.04a |  | |
|  |  |  |  |  |  |  | 2d.D | 0.09 ± 0.06a | 2d.N+ | 0.08 ± 0.05a |  |  |
|  |  |  |  |  |  |  | 7d.D | 0.07 ± 0.03a | 7d.N+ | 0.10 ± 0.04a |  |  |
|  |  |  |  |  |  |  | 14d.D | 0.08 ± 0.06a | 14d.N+ | 0.09 ± 0.05a |  |  |

Lowercase letters denote significant differences within factors

**Table S10** Results of pair-wise Post-hoc Tukey test of emission of compound classes between species (*Brassica napus, Sinapis alba and Sinapis arvensis*). Significant *P*-values are bold typed.

| **Species** | **Compound classes** | | | |  |
| --- | --- | --- | --- | --- | --- |
|  | aromatics | fatty acid-derived | monoterpenes | sesquiterpenes | homoterpenes |
|  | *P* | *P* | *P* | *P* | *P* |
| *B. napus* × *S. alba* | **<0.0001** | 1 | **<0.0001** | **0.015** | 0.770 |
| *B. napus* × *S. arvensis* | 0.651 | 0.113 | **<0.0001** | 0.203 | 0.104 |
| *S. alba* × *S. arvensis* | **<0.0001** | 0.141 | **<0.0001** | 0.497 | **0.031** |

**Table S11** Relative emission rate (mean ± SD) of each compound class for the three species

| **Species** | **Compound classes** | | | | |
| --- | --- | --- | --- | --- | --- |
|  | aromatics | fatty acid-derived | monoterpenes | sesquiterpenes | homoterpenes |
| *B. napus* | 0.10 ± 0.15 | 0.26 ± 0.27 | 0.43 ± 0.25 | 0.15 ± 0.16 | 0.04 ± 0.06 |
| *S. alba* | 0.40 ± 0.23 | 0.25 ± 0.25 | 0.21 ± 0.13 | 0.08 ± 0.09 | 0.04 ± 0.05 |
| *S. arvensis* | 0.07 ± 0.09 | 0.17 ± 0.18 | 0.62 ± 0.21 | 0.10 ± 0.09 | 0.01 ± 0.02 |

**Table S12** Results of linear models of the effects of time and treatments on scent emission of compound classes for *Brassica napus, Sinapis alba* and *Sinapis arvensis*. Significance was assessed using Analysis of Deviance Table (Type II tests). Significant *P*-values are bold typed.

| **Factor** | **aromatics** | | | | | | **fatty acid-derived** | | | | | | **monoterpenes** | | | | | | **sesquiterpenes** | | | | | | **homoterpenes** | | | | | |
| --- | --- | --- | --- | --- | --- | --- | --- | --- | --- | --- | --- | --- | --- | --- | --- | --- | --- | --- | --- | --- | --- | --- | --- | --- | --- | --- | --- | --- | --- | --- |
|  | *B.  napus* | | *S.  alba* | | *S.  arvensis* | | *B.  napus* | | *S.  alba* | | *S.  arvensis* | | *B.  napus* | | *S.  alba* | | *S.  arvensis* | | *B.  napus* | | *S.  alba* | | *S.  arvensis* | | *B.  napus* | | *S.  alba* | | *S.  arvensis* | |
|  | *F* | *P* | χ2 | *P* | χ2 | *P* | χ2 | *P* | χ2 | *P* | χ2 | *P* | *F* | *P* | χ2 | *P* | χ2 | *P* | χ2 | *P* | χ2 | *P* | χ2 | *P* | χ2 | *P* | χ2 | *P* | χ2 | *P* |
| Time | 0.67 | .567 | 32.09 | **<.0001** | 2.82 | .420 | 4.25 | .119 | 7.24 | .065 | 6.07 | .108 | 2.41 | 0.096 | 1.15 | .766 | 2.41 | .492 | 12.27 | **.002** | 9.14 | **.027** | 8.84 | **.031** | 6.55 | **.038** | 5.11 | .164 | 2.46 | .483 |
| Watering | 0.56 | .456 | 0.15 | .702 | 0.08 | .778 | 0.55 | .459 | 2.74 | .098 | 4.56 | **.033** | 1.75 | .190 | 3.96 | **.047** | 1.38 | .240 | 0.39 | .531 | 2.81 | .094 | 0.07 | .787 | 0.23 | .630 | 6.82 | **.009** | 1.46 | .228 |
| Nitrogen | 0.42 | .517 | 10.50 | **.001** | 0.09 | .769 | 0.37 | .546 | 0.81 | .369 | 1.00 | .318 | 1.87 | .175 | 0.87 | .352 | 1.00 | .318 | 0.03 | .872 | 3.18 | .075 | 0.003 | .960 | 7.54 | **.006** | 8.27 | **.004** | 0.68 | .409 |
| Time ×  Watering | 1.47 | .236 | 1.12 | .772 | 0.59 | .899 | 0.50 | .779 | 2.61 | .455 | 1.01 | .799 | 0.07 | .932 | 19.08 | **.0003** | 0.33 | .954 | 0.19 | .909 | 5.24 | .155 | 10.93 | **.012** | 2.22 | .329 | 8.73 | **.033** | 12.53 | **.006** |
| Time ×  Nitrogen | 0.37 | .689 | 6.42 | .093 | 3.30 | .348 | 2.57 | .277 | 4.43 | .218 | 9.51 | **.023** | 2.31 | .106 | 0.94 | .815 | 3.68 | .298 | 0.7051 | .775 | 2.35 | .503 | 3.50 | .321 | 0.53 | .768 | 4.13 | .248 | 0.68 | .877 |
| Watering ×  Nitrogen | 0.02 | .878 | 3.15 | .076 | 0.32 | .573 | 0.51 | .475 | 1.26 | .261 | 5.28 | **.022** | 0.76 | .385 | 0.38 | .538 | 0.88 | .350 | 17.45 | **.0001** | 2.48 | .115 | 0.14 | .711 | 0.24 | .622 | 2.94 | .086 | 0.40 | .529 |
